# A Research Project for Secondary Schools on Urban Plant Ecological Genetics

**DOI:** 10.1002/ece3.74482

**Published:** 2026-10-18

**Authors:** Bruno Andreas Walther, Leonard Jakob Klamann, Sarah Victoria Weirauch, Ghinwa Mashhady, Hans‐Jörg Mai, Petra Bauer

**Affiliations:** ^1^ Institut für Botanik, Heinrich Heine Universität Düsseldorf Düsseldorf Germany; ^2^ Cluster of Excellence on Plant Science (CEPLAS), Heinrich‐Heine‐University Düsseldorf Germany

**Keywords:** citizen science, ecological genetics, phenotyping, PUKI, school project, urban ecology

## Abstract

Participation in authentic research projects benefits both teachers and students by linking hands‐on field work with experimental biology and quantitative analysis. Plant ecological genetics is especially well suited for classroom integration, but accessible, classroom‐ready outlines are scarce. *Arabidopsis thaliana* —a wild species in urban environments with a well‐characterized genome and extensive educational resources—provides an ideal model. Here we present a comprehensive, easy‐to‐implement pipeline for secondary‐school–level research‐oriented projects in plant ecological genetics using *A. thaliana* . The report includes (i) a step‐by‐step experimental workflow for field sampling, phenotypic scoring, and PCR‐sequencing‐based genotyping assays for environment‐sensitive allele monitoring; (ii) classroom‐adapted protocols and quality‐control checks; (iii) background material and a list of concrete, testable research questions; and (iv) downloadable real datasets and analysis templates for guided interpretation. We demonstrate feasibility with pilot field‐survey data from urban school neighborhoods and report outcomes that validate the approach as a proof of concept. Students can learn species identification, formulate hypotheses about ecological adaptation, perform standardized data collection, and apply elementary genetic and statistical analyses—thereby gaining practical laboratory skills and improved scientific literacy. The methods are adaptable to different time frames and resource levels and can be scaled into broader citizen‐science initiatives, allowing classroom findings to contribute meaningfully to ecological genetics research. This project therefore provides a practical bridge between secondary education and place‐based research, fostering engagement with contemporary questions in ecology and evolution.

## Graphical abstract

This paper offers a ready‐to‐use school plant ecological genetics project outline based on a fieldwork citizen science research idea. We show how to collect phenotypic and genetic data of wild *Arabidopsis thaliana* plants growing in an urban environment. We outline how to analyze the data to examine the genetic and phenotypic diversity within the species in the vicinity of a school. The results can serve to discuss ecological genetics and plant environmental adaptation. Students learn basic ecology and genetics with real research methods.

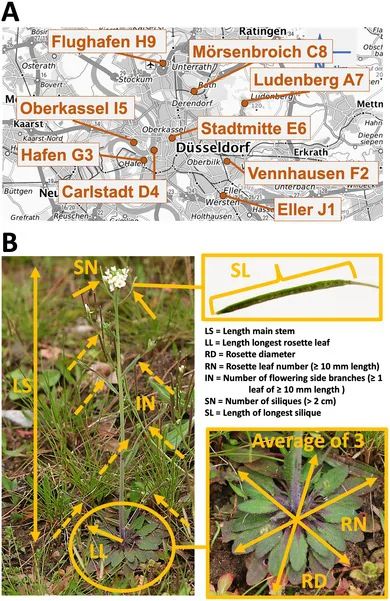

## 1 Introduction

To get science out of the so‐called “ivory tower,” two concepts have taken hold in many different fields of science over the last two decades: to engage people through *citizen science* projects, and to foster *knowledge transfer* which makes scientific knowledge accessible and entertaining to the citizen audience (ECSA 2015; Hecker et al. 2018; Tõnisson et al. 2021; Vohland et al. 2021; Cârstea et al. 2022). Citizen science is defined as scientific research conducted in partnership between scientists and institutions with people who are not professionally working as scientists in the respective scientific field. It involves public participation and collaboration in scientific research with the aim of increasing scientific knowledge. This participation can range from data collection to the interpretation and dissemination of scientific results and even design of research projects. Topics can cover almost every realm of science, and the degree of participation can also vary greatly (Shirk et al. 2012). Knowledge transfer (also called knowledge diffusion) can be seen as the transfer of facts or practical skills from one entity to another (Thompson et al. 2006; Nokes 2009). In this context, it refers to scientific knowledge transferred to citizens namely school students and their teachers through the efforts of scientists. Citizen science projects have become widespread in Germany in the last decade (Pettibone et al. 2017; Bonn 2020, 2022; Schleicher and Schmidt 2020), with several projects focusing on plant monitoring and conservation (e.g., Schneider et al. 2018; Mäder et al. 2021; Katal et al. 2023; Voigt‐Heucke et al. 2023). However, despite their widespread adoption, citizen science projects are still only rarely incorporated into educational initiatives for schools.

Importantly, research has demonstrated the great potential of citizen science to foster youth participation in current environmental issues, especially when thorough place‐based data collection and relevant socio‐ecological issues are taken into consideration (Ballard et al. 2017). The connection to authentic scientific questions helps young people discover the nature of scientific research and their personal role within this research (Barnard et al. 2011; Symes et al. 2015; Lüsse et al. 2022). Science education is relevant to pupils or students if it (1) addresses their personal curiosity and interests on an *individual* level, (2) prepares them to be responsible members of *society*, which includes an understanding of how science and society interact, and (3) provides them with *career* orientation and *professional* skills (Stuckey et al. 2013). Hence, it needs to be explored further how existing citizen science projects can be integrated in schools and universities.

Engaging with citizen science projects offers outstanding opportunities for teachers and their students: (1) Lessons tend to be more engaging and interesting if they have a real‐world connection; (2) citizen science projects can break up the monotony of classroom teaching; (3) teachers can introduce new perspectives, such as the workflow of scientific projects including hypothesis testing; (4) students experience how open and participatory science works as they are actively involved in the research process; and (5) and ideally, citizen science projects provide teaching materials and interactive teaching tools free of charge for teachers to use. The ideal results are (1) increased students' motivation through appropriate teaching, which is not teacher‐centered but mostly student‐centered (e.g., by letting students ask questions and develop ways to answer them); (2) more interactive two‐way teaching instead of the much more passive traditional one‐way teaching (i.e., teacher‐talk and textbooks); and (3) improved interest and knowledge among the students which may then be passed onto family members and friends and affect career choices (Flowers et al. 2016; van Eijck and Roth 2009).

To foster interactions between schools and citizen science, citizen science platforms like the German “mit:forschen!” (https://www.mitforschen.org/) provide guidelines for project initiators of citizen science projects working with schools (Kiessling et al. 2023). Other important guidelines and strategies were published in Dittmann et al. (2023) and Thiel et al. (2023). Several successful citizen science projects worked intensively or exclusively with school children (e.g., Kruse et al. 2020; Schneiderhan‐Opel and Bogner 2020; Kiessling et al. 2021; Sieg and Dreesmann 2021; Christ et al. 2022). Shah and Martinez (2016) criticized that citizen science projects often do not entail sufficient flexibility for their implementation in schools. Therefore, it is important that project coordinators and partners properly plan and prepare the project infrastructure, for example, by providing teachers with tested well‐prepared experimental pipelines and suitable materials (Lüsse et al. 2022). A recent survey summarizes the conditions and key factors for successful implementation of citizen science in schools and suggests that the design of citizen science projects and teacher prerequisites play major roles (Müller et al. 2025). However, until today, only few citizen science‐related school projects are documented and thus feasible in a school class setting.

Plant ecological genetics examines genetic and environmental influence and the interaction thereof on adaptation and ecological function. By linking genotype to phenotype and plant performance across heterogeneous landscapes, this field reveals the genetic basis of traits and trait plasticity mediating responses to abiotic stresses (e.g., drought, soil nutrient availability, temperature). These insights are essential for predicting plant responses to environmental change, informing conservation and restoration strategies, and improving plant resilience through knowledge of adaptive potential and constraint. *Arabidopsis thaliana* has become a premier model for plant ecological genetics due to its small genome, extensive genetic and genomic resources, short generation time, and wide geographic distribution encompassing diverse habitats including human‐made urban environments. A well‐annotated reference genome and a multitude of sequenced naturally occurring accessions enable studies of trait variation, allelic distribution and experimental tests of adaptive hypotheses (https://abrc.osu.edu). Studies in *A. thaliana* have elucidated molecular pathways underlying local adaptation, life‐history variation, stress tolerance, and genotype–environment interactions, providing generalizable principles that can be transferred to other species and applied to ecological and evolutionary questions.

Plant ecological genetics is a priority research area in the face of climate change, urbanization, and habitat degradation. Urban wild plants offer particularly rich opportunities for developing citizen‐science and school‐based projects that raise awareness on ecological performance traits and relevant genetic adaptation and provide authentic research experiences.

Here, we present and evaluate a citizen‐science‐adapted school project in plant ecological genetics. Although it is not easily possible in the frame of a school project to investigate plant plasticity and genetic adaptation, the project addresses genetic variation and ecological performance by asking students to survey *A. thaliana* individuals in urban areas near their schools. We report the pilot field‐survey results and provide a practical protocol, background rationale, original datasets and guidance for data interpretation. This report is intended to support both experimental implementation and the theoretical analysis of urban field surveys in classroom school settings to raise awareness on plant ecological genetics.

## 2 Results

### 2.1 Designing an Interactive Research Project on Plant Ecological Genetics Provides a Place‐Based Research Opportunity for Secondary Schools

Recently, a citizen science project has been initiated in Germany with the specific aim of conducting both scientific research as well as engaging in knowledge transfer, termed PUKI (which stands for Pflanze • Umwelt • Klima • Interaktion, in English Plants • Environment • Climate • Interaction, https://www.puki.hhu.de/). The specific scientific goal is to understand genetic and ecological adaptations of plants to their environment. This is relevant for elucidating how anthropogenic drivers, including climate change and urbanization, influence plant adaptation. School and university students can either participate in PUKI directly or use the methodology for own research. The latter is preferred in countries outside of Germany. There is a gap in that an experimental outline for a research‐oriented ecological genetics school project and its validation in a concrete test case are both lacking. To address this gap, we present here an experimental pipeline and field survey data as a proof of concept for a school project, together with background information and detailed methods for classroom implementation. Supplemental background knowledge and methods are specifically targeting secondary schools (Text S1). The school project addresses important scientific skills which secondary school students need to learn during science lessons (Table S1). Thus, the projects generates opportunities to work on important concepts, terms and techniques required to understand genetic research (Table S2). The general outline of the proposed Ecological Genetics‐based school project and the data from a two‐year test field survey are summarized (Figure 1A): Students identify individuals of the target species, namely the thale cress *Arabidopsis thaliana* , which can be found flowering in spring in various urban habitats in Middle Europe, primarily pavement, walls and moss beds, and urban meadows or gardens (Figure 1B). *A. thaliana* is a genetic plant reference model, and numerous excellent teaching and scientific resources are available, including materials and genetic lines for schools (https://abrc.osu.edu/educators). Students can identify the plant species for example, with a plant identification app like *Flora Incognita* (https://floraincognita.com/) (Mäder et al. 2021), take phenotyping data about plant individuals (such as morphology and habitat; Dataset S1) and collect leaf samples. They can then continue with laboratory analyses such as genomic DNA extraction, amplification of environment‐relevant genes, sequencing of the PCR‐fragments and comparison of sequences for allele analysis (Dataset S2). Specifically, we propose investigating the genetic variation of an environment‐sensitive flowering time gene called *FRIGIDA* (*FRI*). There are functionally relevant polymorphisms of the *FRI* gene which affect flowering time in environment‐relevant contexts like a North‐South temperature gradient in Europe (Stinchcombe et al. 2004; Shindo et al. 2005; Lovell et al. 2013); in other words, individual plants flower early or late depending on their genetic makeup (Text S2). To illustrate the field methods, we present a test case of ecological field work on *A. thaliana* in the German city of Düsseldorf. In the school laboratory, students can analyze those data, for example, through plant phenotyping, genotyping, and graphical and mathematical‐statistical analysis. We deliver teaching topics and tools to potential participants. We give examples about conclusions and suggest follow‐up studies.

**FIGURE 1:**
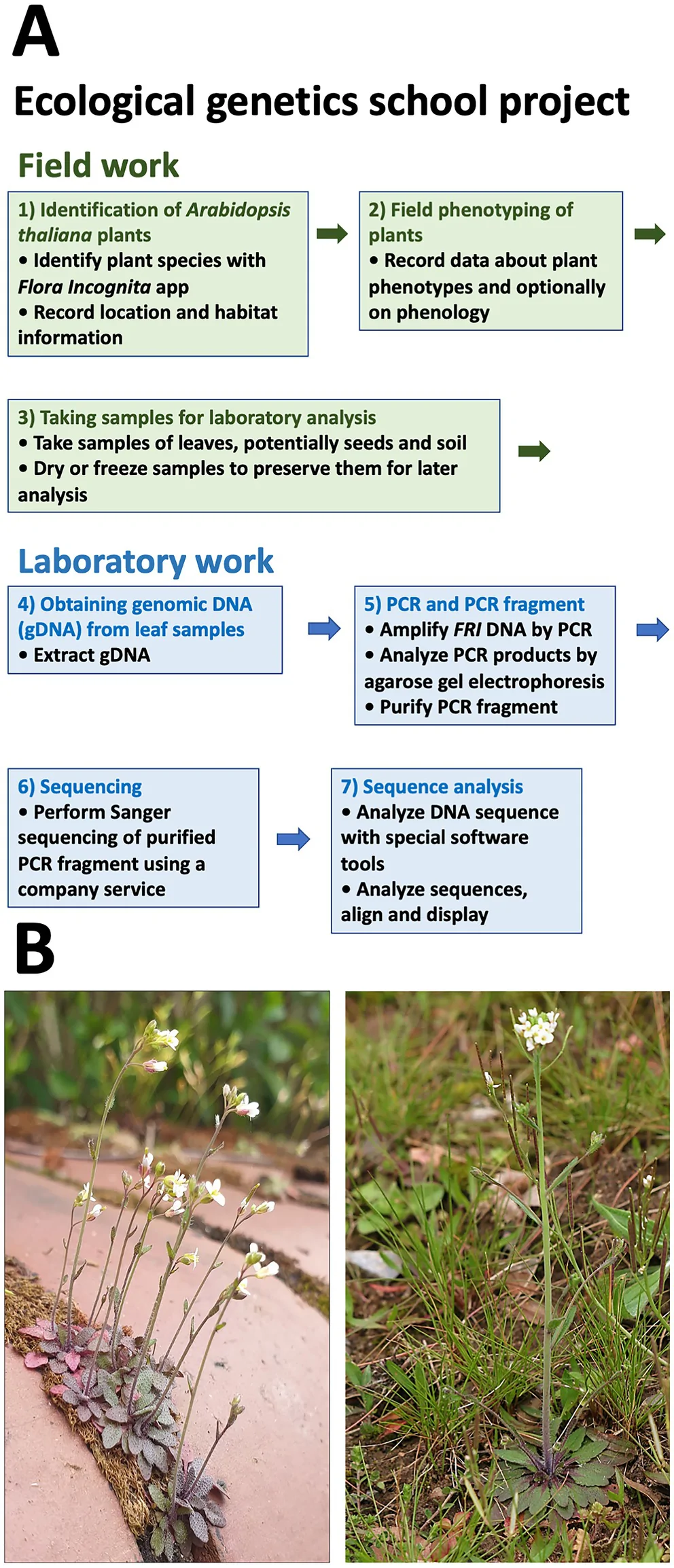
Flowchart and target species of the Plant Ecological Genetics school project. (A) Flowchart of project work from the identification and measurement of the plant to the analysis of the DNA sequence. (B) Target plants in urban environments. The thale cress *A. thaliana* grows in various habitats, including, from left to right, a roadside and a meadow. Photos by Bruno Walther.

With these methodologies and materials, teachers can plan their lessons theoretically and practically and flexibly arrange their school projects which would introduce ecological, genetic, and statistical methods into classroom teaching and encourage interdisciplinary projects. If they wish, they can participate as volunteers in the corresponding PUKI citizen science project; however, this is not a prerequisite for conducting the school project.

Taken together, the here‐proposed school project is to study ecological processes with botanical, mathematical, and genetic tools and explicitly addresses a specific local geographic and ecological context, which qualifies it as place‐based research.

### 2.2 Field Phenotyping Identified Correlations Between Morphological Variables and Growth Parameters Within the City Area

We set out to test a pipeline of sampling, phenotyping and genotyping wild *A. thaliana* plants in a city environment to demonstrate feasibility of an ecological genetics school project. The data were analyzed and are presented here as a proof of concept. *A. thaliana* is known to be a frequent inhabitant in Europe. We sampled *A. thaliana* plants in various habitats such as pavement and flower bed (Figure 1B) at nine locations in the city of Düsseldorf, Germany, in the spring of 2022 and 2023 (Figure 2A, Figure S1, details in Table S3). The plants were phenotyped for morphological characteristics as shown in Figure 2B. During 2022, 45 individual *A. thaliana* plants were phenotyped and sampled once at the nine locations in Düsseldorf. Individuals varied considerably in the number of siliques, rosette diameter, and silique length (Table 1, 2022 data; Table S4, Dataset S1, 2022 data). During 2023, 155 individual *A. thaliana* plants were phenotyped and sampled at the same locations in Düsseldorf over a seven‐week period, this time including more parameters. However, at locations 4 and 6, no *A. thaliana* individuals were found in 2023. Individuals again varied considerably in lengths of the main stem, longest leaf and siliques, rosette diameter, numbers of branches, rosette leaves, inflorescences and siliques at the main stem (Table 1, 2023 data; Table S4; Dataset S1, 2023 data). The individual growth data over time were used to illustrate the plants' growth curves (Figure S2).

**FIGURE 2:**
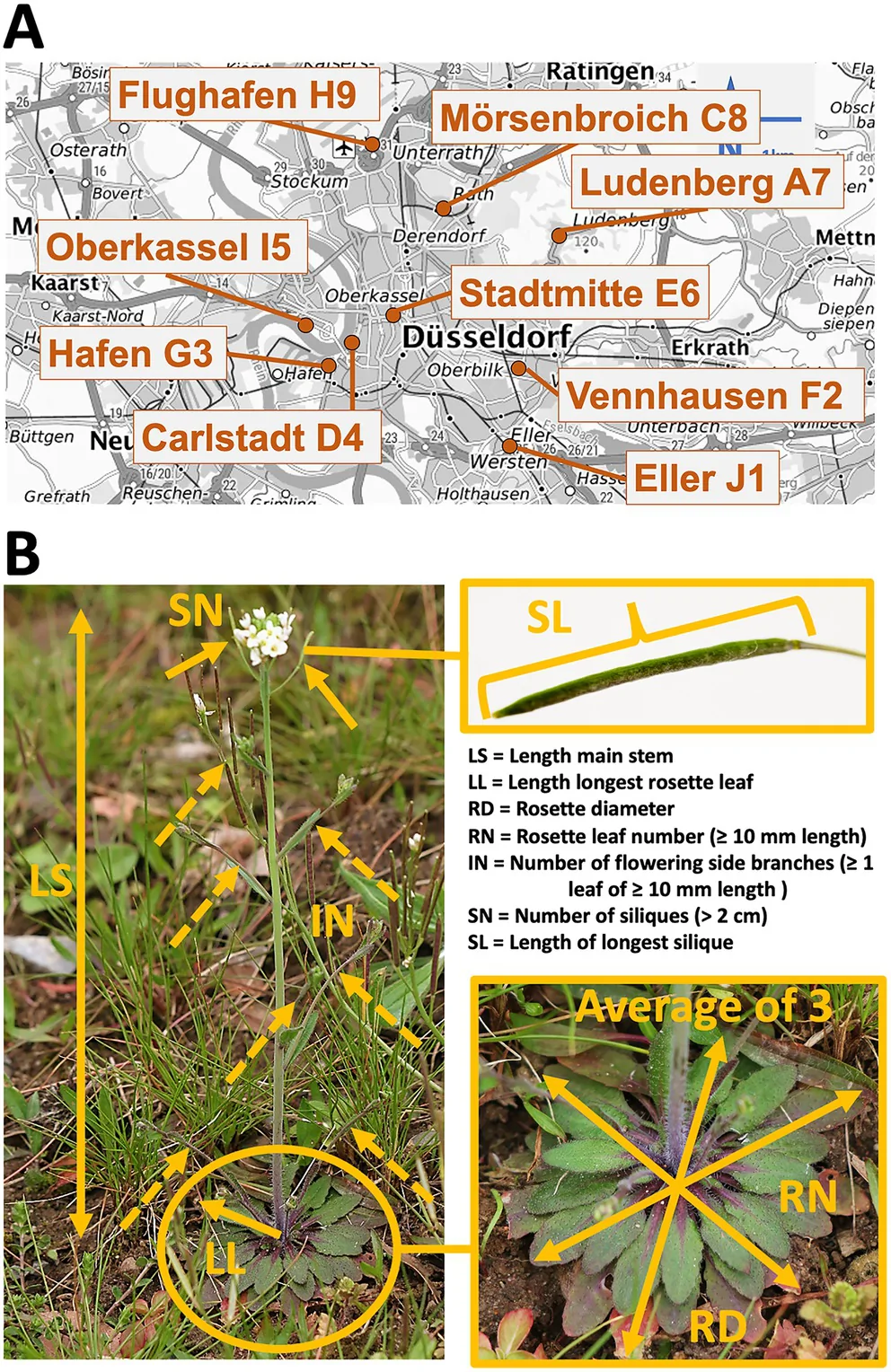
Field work for a proof of concept of the Plant Ecological Genetics school project. (A) Nine locations for phenotyping and sampling of Arabidopsis thaliana individuals in Düsseldorf; geographic coordinates for red dot‐marked locations in Table S3. The map is public access material supplied by the Geoportal NRW (https://www.geoportal.nrw/, data license Version 2.0, https://www.govdata.de/dl‐de/by‐2‐0, copyright Quellenvermerk: Kartendarstellung und Präsentationsgraphiken: © Bundesamt für Kartographie und Geodäsie <2024>, Datenquellen: https://sg.geodatenzentrum.de/web). (B) Parameters for field phenotyping of various traits, easy to score in classroom settings. Two to three leaves can be collected for genotyping. Abbreviations are explained in the figure. Photos by Bruno Walther.

**TABLE 1:** Summary of growth phenotyping data of *A. thaliana* plants.

Variable	*N*	Median	Mean	Std. dev.	Minimum	Maximum
2022						
Number of siliques	45	3.00	7.91	12.19	0.0	70.0
Rosette diameter [mm]	40	32.70	34.00	14.30	10.00	66.00
Silique length [mm]	28	14.00	13.14	3.34	0.0	18.0
2023						
Stem length [mm]	155	228.00	218.65	98.12	40.0	440.0
Number of branches	155	3.00	3.54	2.75	1.0	12.0
Number of stem leaves	155	5.00	5.01	4.82	0.0	31.0
Number of rosette leaves	124	4.50	5.56	5.47	0.0	20.0
Number of inflorescences	155	3.00	3.41	2.58	0.0	13.0
Number of siliques	155	8.00	15.36	21.05	0.0	105.0
Rosette diameter [mm]	67	48.00	46.77	18.40	16.3	95.0
Silique length [mm]	112	13.00	12.40	2.63	5.0	18.0

*Note:* The numbers represent medians, means, standard deviations (Std. dev.) and range (minimum‐maximum) of nine morphological characteristics of *A. thaliana* individuals measured at seven locations in Düsseldorf in 2022 and 2023. *N* = sample size.

Interestingly, most of the morphological variables were highly correlated, but some were not (Table 2; Table S4; Dataset S1). First, main stem length, number of branches, number of stem leaves, number of inflorescences, number of siliques at main stem, rosette diameter, and silique length mostly correlated positively and significantly with each other, with the exception of (1) the number of stem leaves with silique length and (2) the rosette diameter with silique length, which did not correlate significantly. Finally, the number of rosette leaves never correlated positively and significantly with any variables, but correlated negatively and significantly with stem length, number of siliques, and silique length. These findings show that flowering‐associated parameters mostly are positively correlated with each other.

**TABLE 2:** Correlations between plant growth traits.

Variables	Nb	Nsl	Nrl	Ni	Nsi	Rd	Sl
Stem length	**0.39**	**0.28**	***0.16***	**0.33**	**0.51**	**0.23**	**0.26**
Number branches (Nb)		**0.33**	*0.01*	**0.67**	**0.19**	**0.34**	**0.12**
Number stem leaves (Nsl)			0.02	**0.38**	**0.03**	**0.35**	0.03
Number rosette leaves (Nrl)				0.003	***0.48***	0.0003	***0.13***
Number inflorescences (Ni)					**0.11**	**0.30**	**0.06**
Number of siliques (Nsi)						**0.08**	**0.39**
Rosette diameter (Rd)							0.05

*Note:* *R* ^2^‐values showing positive (non‐italic) and negative (italic letters) and significant (bold letters) and non‐significant (regular letters) correlations among variables using the linear regression model. Corresponding simple linear regression analyses of morphological characteristics (or variables) of *A. thaliana* individuals measured at seven locations in Düsseldorf in 2023 (Dataset S1, 2023 data) are provided in Table S4. Each data point is one individual plant measured at one time point for each of its morphological characteristics. The variable names from rows 2–7 are abbreviated in columns 2–7, and column 8 represents the silique length (Sl).

Thus, sampling *A. thaliana* in a middle European city is a successful endeavor and analysis of phenotypic data yields positive and negative correlations within the data set, suited to be discussed with students in a classroom.

### 2.3 *FRI* Allele Sequencing Demonstrated Genetic Variation of Sampled Plants Within the City Area

In 2023, two to three plants were sampled from the seven locations at which *A. thaliana* plants were found in that year to conduct genetic analysis in the laboratory as outlined in Figure 1A and Figure 3A. For the genotyping, we investigated the genetic variation at the flowering control *FRIGIDA* (*FRI*) locus. *FRI* affects the flowering time of *A. thaliana* , and allelic variation is an environment‐related adaptation, whereby, in warm climatic situations, plants flower earlier and more rapidly with fewer rosette leaves produced than in cold climatic regions (Text S2). In cold regions, plants like *A. thaliana* require vernalization, an extended growth at or below a temperature of 4°C. There is an underlying regulatory control mechanism involving *FRI* to control whether plants flower early or late. In particular, it has been described that *A. thaliana* plants can carry nonfunctional or weak *FRI* alleles that do not strongly activate a flowering repressor (flowering locus C protein, FLC). In this case, the plants may flower earlier without the requirement for a prolonged exposure to cold temperature. Hence, analysis of *FRI* can be interesting as natural variation may exist even within small regions, as shown earlier (Johanson et al. 2000; Corre 2005), and the described *FRI* allele variation is detectable with the experimental pipeline described in Figure 3A. Genomic DNA of the sampled plants was prepared and used for amplification of *FRI* gene‐specific DNA fragments (Figure 3B), sequenced, and the sequences were then analyzed (Figure 4A). The obtained *FRI* PCR bands represented five different *FRI* alleles (Figure 4A), which occurred at five out of the seven sampled locations (Figure 4B; sequences of the identified Düsseldorf alleles are provided in Dataset S2). Interestingly, polymorphisms had already been described in previous ecological *FRI* studies (Johanson et al. 2000; Corre 2005). All polymorphisms resulted in amino acid changes in exon 1 (see mutations in Figure 4A). Four out of five alleles had a 16‐bp deletion between exon 1 and intron 1 as the one found in the Col‐0 reference accession, as compared to allele V which had a 16‐bp insertion. Overall, we classified the samples into allele types I–V. This shows that even plants collected within a small urban area like Düsseldorf can exhibit substantial molecular diversity at the studied *FRI* locus. The spatial distribution of these alleles also varied across the sampling sites: Some alleles occurred in only one location, while others appeared in several neighborhoods.

**FIGURE 3:**
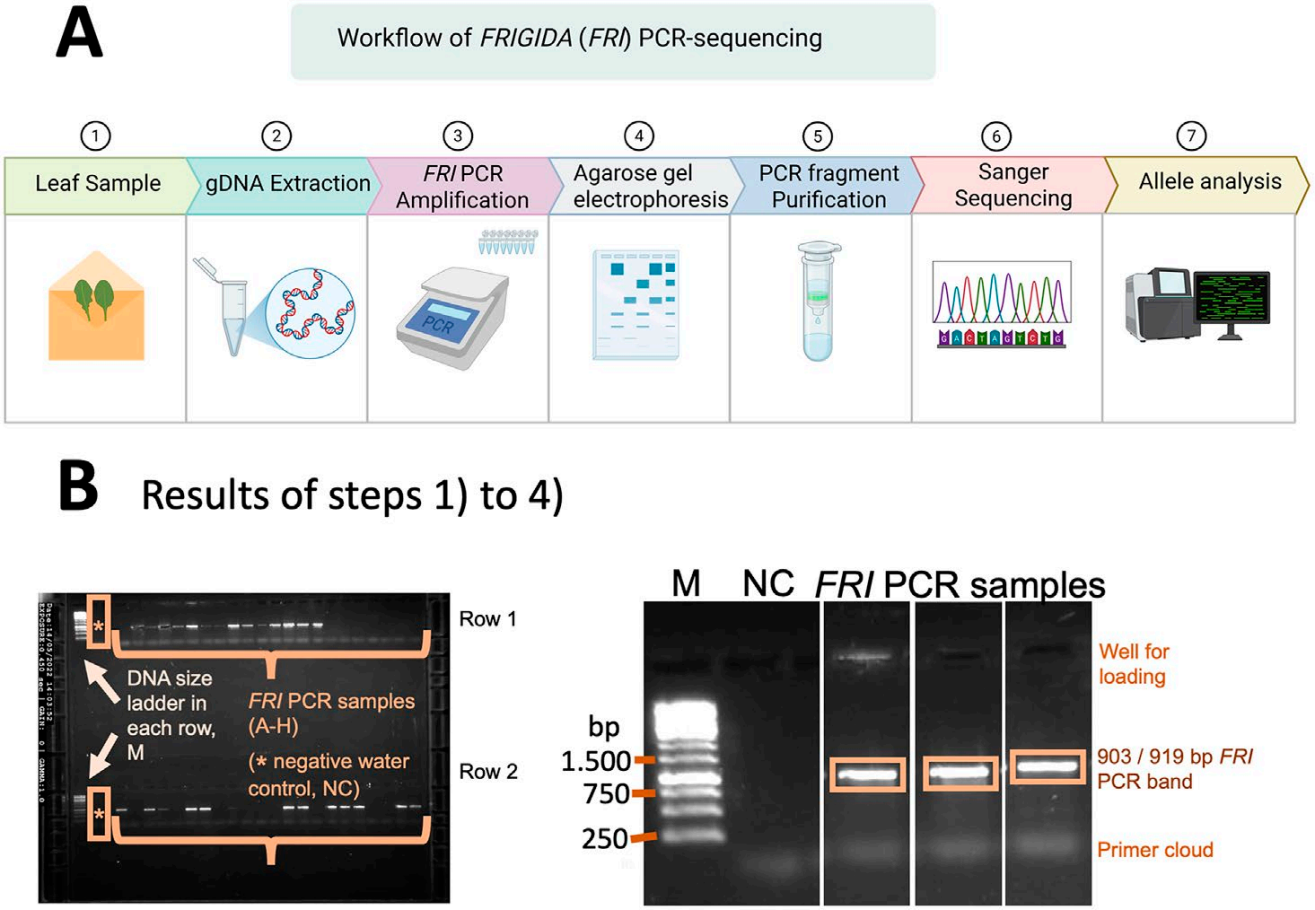
Laboratory workflow and results of steps 1–4 of *FRIGIDA* genotyping by PCR‐sequencing. (A) Workflow of laboratory work. Created with Biorender. (B) Results of steps (1) to (4) from the workflow in A. Left, Whole image of agarose gel after electrophoresis; two rows with wells for loading; from left to right loading of DNA ladder, negative water control and FRI PCR samples from the various sampling sites. (B) Closeup of certain areas of the left side, with DNA ladder (M, indicated are three fragment sizes in basepairs, bp), negative water control (NC), lanes with PCR fragments corresponding to FRI fragments of three locations. PCR fragments were cut out, purified and sequenced. Indicated are the positions of wells for loading and the primer cloud. In left and right images, stained DNA fragments appear as white bands; 1.5% agarose gel; FRI PCR fragment amplified with Forward and Reverse FRI primer pair called G/E site_F and 16‐bp_R. Photos by Sarah Weirauch.

**FIGURE 4:**
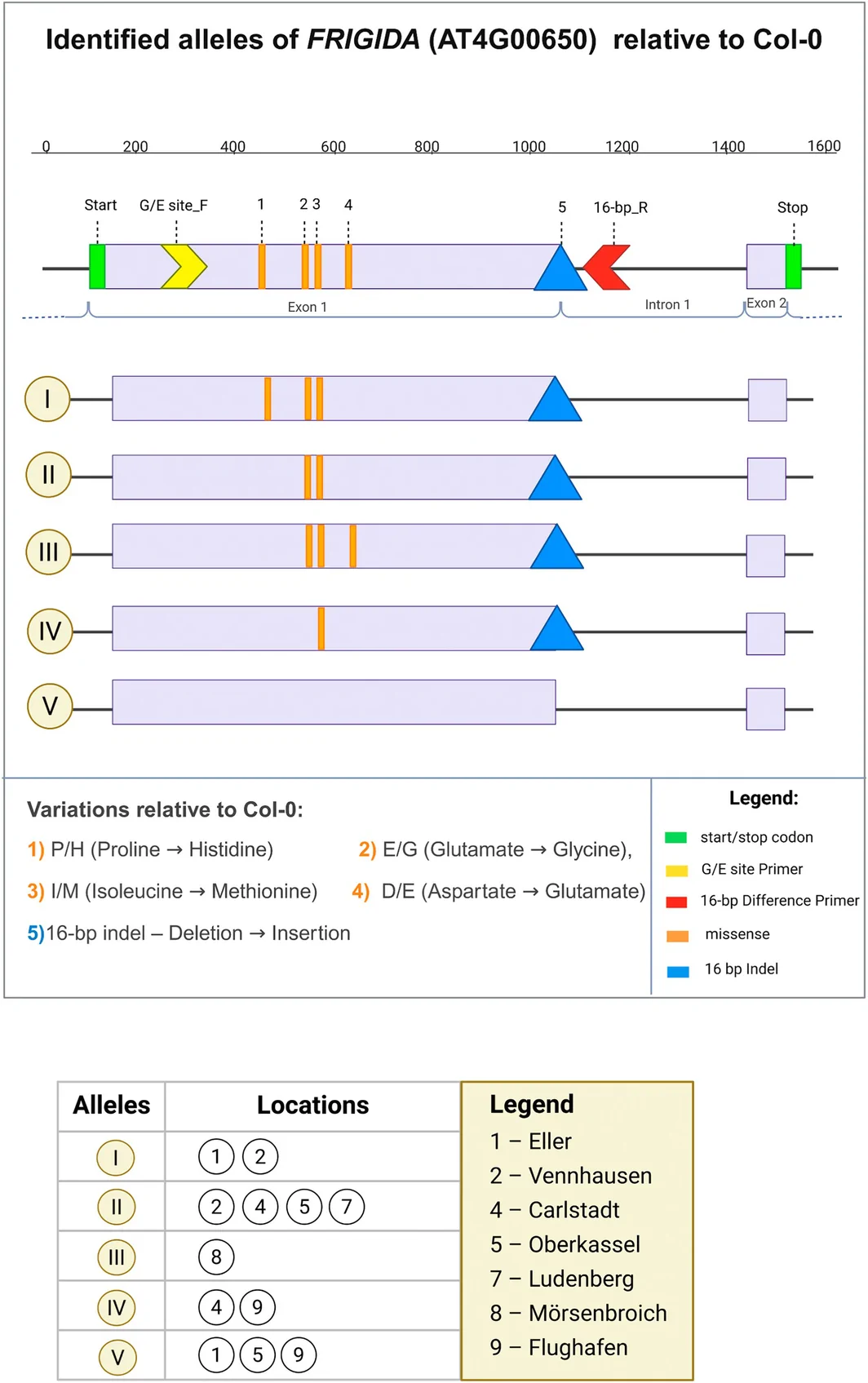
Final results of laboratory work steps 1–7 of *FRIGIDA* genotyping by PCR‐sequencing with identification of five different alleles in Düsseldorf. (Upper part) Allele structures I‐V were found in Düsseldorf *A. thaliana* samples by PCR‐sequencing. DNA sequences were determined from PCR fragments obtained using the primers G/E site_F and 16‐bp_R. Top, gene structure of FRIGIDA locus in Col‐0; the numbers 0–1600 provide the size of the genomic region of Col‐0 in bp. The coloured features are indicated in the legend in the figure. Thee polymorphisms are indicated in the five different alleles identified. The types of allele variation are explained below. (Lower part) Summary of Düsseldorf locations 1‐9 where the respective alleles were found. For information of the locations see Figure 2A and Table S3. Figures A and B created with Biorender.

Hence, genotyping the *FRI* locus in a classroom project can yield interesting observations.

### 2.4 Developing Study Materials Allows Implementing the Plant Ecological Genetics Research Projects in Secondary School Projects

With the supplementary study materials, we present relevant teaching topics and teaching tools. The specific learning outcomes from our proposed school projects should be knowledge of the biology, morphology, and ecology of the target plant species, specifically *A. thaliana* (Text S2), and improved understanding and skill of laboratory and statistical techniques essential for analyses of morphology, ecology, and genetics (Texts S3–S6).

The general learning outcomes should be that the school projects effectively connect students' prior scientific knowledge with new and interesting scientific inquiries. They should foster scientific curiosity, environmental awareness, and essential skills such as exploration and communication. Cognitive processes should be supported as students integrate new information with existing concepts and adjust their understanding based on new insights.

The Texts S1–S6 address the target audience of students and teachers from secondary schools and were improved based on feedback by small groups of students (see last section of Methods). However, we did not conduct a formal and systematic evaluation of learning outcomes and educational effectiveness, which are beyond the scope of this study. Such evaluations may become the subject of a future investigation.

Integrating the school projects across subjects promotes interdisciplinary learning. For example, statistical analyses implemented in Rstudio can be introduced in computer science classes, data collection or reporting in English classes, calculations related to sunlight or environmental factors in physics, and measurements of soil pH in chemistry lessons. It encourages students to ask meaningful research questions, such as: What urban factors influence temperature? Can heat or water stress alter gene expression? Such questions open avenues for further inquiry and cross‐disciplinary application. Follow‐up experiments could examine genetic adaptations under varied environmental conditions, reinforcing the value of student‐led investigation in science education.

Therefore, our practical school projects should encourage curiosity, creativity, and deeper understanding, which are all important learning outcomes. On the other hand, formal tests of these learning outcomes should be the subject of future research.

## 3 Discussion

We present an experimental pipeline with tested protocols and real field survey data that can be regarded as a proof of concept for a secondary‐school student project, guided by teachers. This project has been inspired by a citizen‐science initiative and can be conducted in this context. We emphasize on student‐led research that shows scientific rigor, includes quality checks and is reliable because of simple procedures and insightful instructional materials. The presented approach enhances student engagement with authentic research, builds practical skills (sampling, documenting, basic field, lab and data analysis) and fosters understanding of scientific limitations and contextual interpretation. We state that the project outline, although tested and improved with the help of school students and teachers, has not been scientifically evaluated for pedagogical output.

### 3.1 Phenotypic Variation of *A. thaliana* Can Be Assessed in the Field in an Urban School Environment

The results which summarize the nine morphological characteristics of *A. thaliana* individuals are as expected, meaning the values match closely to those of previous studies (Hegi 1986; Meinke 1994; Meyerowitz and Somerville 1994; Nick 2019). The key point is that these characteristics are easy to measure, even for secondary school students. Indeed, students of the advanced biology course at the Melanchthon‐Schule (see Methods) sampled some target plants and collected data on plant and habitat phenotypes without any problems. Clearly, precise and easily understandable instructions will allow the standardization of the measurement procedures which, in turn, produce accurate and reliable data, even with secondary school students. Exercises in measurements and repeated measurements can lead to interesting discussions about the concepts of bias, precision, accuracy, reproducibility of data, and the possible effects on statistical assessment and interpretation (Walther and Moore 2005; Schoenfeld and Ioannidis 2013).

The results on correlations between the nine morphological characteristics (or variables) of *A. thaliana* individuals are as expected. As an individual plant develops, so elongates its stem, including the number of stem leaves, increases the number of branches and inflorescences, and the number of siliques and silique length; therefore, the positive correlations which we observed were expected for a developing plant. Finally, we know that the rosette grows first in *A. thaliana* but then recedes and eventually senesces as the remainder of the plant undergoes fruit and seed maturation (Hegi 1986; Meyerowitz and Somerville 1994; Nick 2019; Knopf and Bauer 2025). Consequently, we may expect differing correlations between rosette diameter and some of the other morphological characters, as observed. The important point is again that these correlations are easy to calculate and understand in a classroom setting and can lead to further discussions about how to do science and how to formulate questions and answer them (more details for such discussion in classroom settings in Text S3).

Another important point which can be used for further discussions is what might explain the variation in the data. The results showed that data fitting could explain some of the variation in the morphological data. The remaining variation in the data is likely explained by other, unknown factors, such as the genotype, soil, climate, etc. What could be the most important factors? How would one go about finding out? Such questions can lead to further discussions, for example, about the multi‐dimensional ecological niche or controlled, multi‐factorial experiments.

### 3.2 Genetic Variation Can Be Illustrated in the Classroom by Studying the Natural Variation of *A. thaliana* in the Environment

By sampling at seven locations, five different polymorphic alleles of the *FRI* locus were detected which all resulted in altered amino acid sequences of the FRI protein, potentially leading to different functionality. This is interesting for a school project because there is the chance that genetic variation can be detected within populations sampled within the surrounding city. In the unlikely case that a uniform genetic disposition is detected, such a finding can be compared with our data presented here and be stated and discussed in the classroom. Furthermore, if repeated studies across many years are possible in a school, a long‐term research question can be raised on the occurrences and changes of allele states in urban environments over time.

Previous studies have shown that the commonly used Col‐0 reference accession carries a 16‐bp deletion in *FRI*, which results in a loss‐of‐function allele (Johanson et al. 2000). The allele V identified in this study harbors a similar deletion and is therefore likely to represent a non‐functional variant. In contrast, alleles I–IV do not contain this deletion and retain an intact open reading frame without premature stop codons. This suggests that these alleles may encode partially or fully functional FRI proteins. However, sequence‐based analyses alone cannot resolve quantitative differences in protein activity.

One interesting result from previous studies was that certain *FRI* variants were more preponderant in Spain than in Scandinavia, while mixtures of variants were found in middle Europe (Johanson et al. 2000). Schmitz et al. (2024) also found genetic variation between different *A. thaliana* populations in Cologne, which is close to Düsseldorf. Interestingly, these genetic variants were also not randomly distributed but were associated with differences in ecology, for example, the soil nutrient content and the timing of disturbance (mainly mowing). Our data show that the *FRI* allelic diversity which we found in 2022–2023 matched partly that which was found 20 or more years earlier for middle Europe in a study by Stinchcombe et al. (2004), Johanson et al. 2000 and Corre 2005 which described variation at the *FRI* locus along a north‐south gradient as an adaptation to temperature. Therefore, genetic variation of *A. thaliana* was found in both Cologne and Düsseldorf and it can be expected, although this needs to be tested, that it is the case in other German or other country's cities as well. In any case, whether or not, genetic variation is detected in the vicinity of a school, the outcome is suitable and can be discussed. Although we demonstrated genetic diversity using the *FRI* locus as an example, other genetic loci may be equally well suited. Ever since the entire genome of *A. thaliana* was sequenced (Arabidopsis Genome Initiative 2000), thousands of studies have investigated how different genes regulate the development and reproduction of *A. thaliana* under different environmental conditions (e.g., Corre 2005; Hancock et al. 2011; Exposito‐Alonso et al. 2019). Since so much is known about the genetics of *A. thaliana* already, many examples of how genetics and adaptation are at work in nature can be taught using this species as an example. Students can be encouraged to investigate these topics further by collecting observations on plant morphology and local environmental conditions, identifying potential adaptations, and searching the scientific literature for evidence of underlying genetic adaptations. They can also be invited to explore the origins and history of genetic variation and to discuss evolutionary processes such as mutation, natural selection, adaptation, environmental change, and the migration of individuals and populations.

### 3.3 Possible Integration of the Ecological Genetics School Projects

A possible extension of the presented school projects into more multidisciplinary school projects is to work with citizen scientists. For example, some participants of citizen science projects like PUKI may be willing to give talks in schools or take students out on field trips (Figure 5). There are many botanical amateur groups and environmental non‐governmental organizations in Germany and elsewhere whose members would likely be willing to work with school students if asked. The German citizen science “mit:forschen!” (https://www.mitforschen.org) website lists further plant‐related citizen science projects which could be asked for participation and feedback.

**FIGURE 5:**
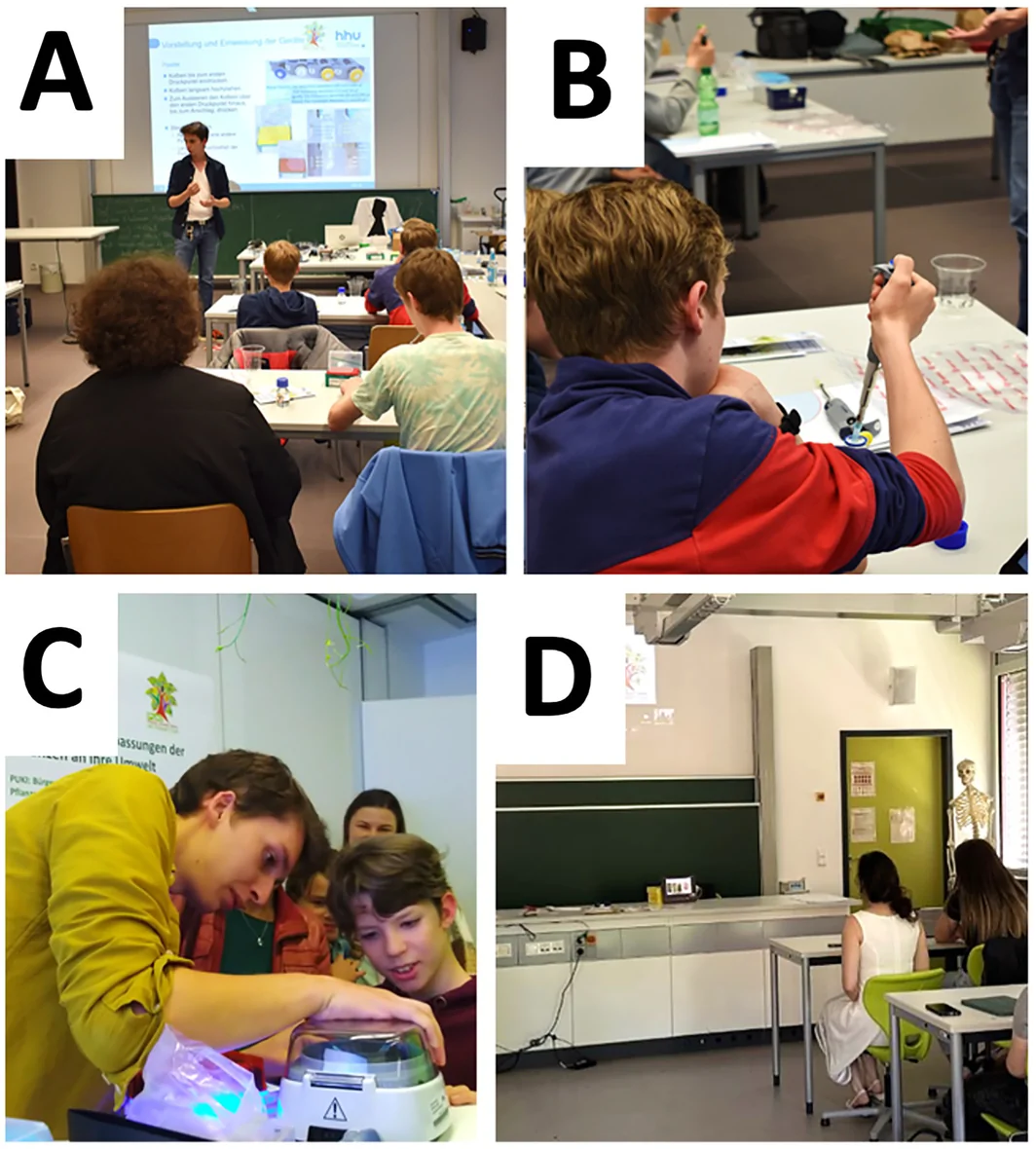
Proof of concept demonstration of the Plant Ecological Genetics school project in classroom settings and at public events. (A) The second author (LJK) demonstrates various laboratory techniques to participants of a workshop organized by the KölnerKinderUniversität (KKU), Cologne, Germany, on 20 June 2024. Eight participants from grades 9‐12 took part, asked questions and were motivated to take part in the PUKI project (photo taken by Bruno A. Walther). (B) The participants of the KKU workshop undertake genotyping techniques, for example, grinding, pipetting and centrifuging samples, and performing PCR and gel electrophoresis (photo taken by Bruno A. Walther). (C) Parts of the project works were also presented at the ‘Night of Science’ public event organized by the Heinrich Heine Universität Düsseldorf, Germany, on 13 September 2024. Here, the second author (L.J.K.) demonstrates the function of a centrifuge to several children and adults (photo taken by Bruno A. Walther). (D) The PUKI project was presented online to the students of the advanced biology course at the Melanchthon‐Schule Steinatal, Germany, on 13 June 2025. Subsequently, the students, motivated by the lead teacher, sampled some target plants and entered the relevant data into the PUKI mobile phone application (photo taken by Leandra Schulze).

As we emphasized in the Introduction, the engagement with citizen science projects offers several unique opportunities for teachers and students. In turn, citizen science projects such as PUKI also gain greatly by working with schools because schools are effective multipliers for spreading knowledge and engagement. Our school projects presented here are an attempt to foster such mutually beneficial interactions in the future.

## 4 Conclusion, Limitations, and Perspectives

*A. thaliana* occurs in most regions of Germany and in many other countries in the Northern hemisphere. Therefore, the proposed school project has a broad applicability in many countries. Furthermore, the model plant individuals can be easily grown and reproduced in school settings. One of the limitations when studying this plant species in Northern country like Germany is that *A. thaliana* flowers during a few months in spring. Yet, there are solutions to overcome this limitation. The first one is to grow the plants in the school, which would also teach the students how to grow and maintain plants. If seeds cannot be obtained from wild plants, seeds can be obtained from either NASC (Nottingham Arabidopsis Stock Centre) based at the University of Nottingham or ABRC (Arabidopsis Biological Resource Center) based at Ohio State University. The second solution is to have different groups or school classes conduct different parts of the research: for example, one group searches for sites, one group then collects morphological plant data and plant material, one group conducts molecular analyses, and another group conducts data analyses. Alternatively, only parts of the here presented possible experimental field and laboratory work could be performed. Not only the instructions but also our data are available for download; hence, practical and theoretical data analyses can be performed independently.

The presented school projects were developed in collaboration with scientists, biology students, teachers, and citizen scientists during 2022–2024. They were presented to children and adults at public science events. Positive interactions and feedback were received. In the future, the activities and feedback can be used to evaluate and continuously improve the projects and study materials.

## 5 Methods

### 5.1 Study Sites

All sampling of *A. thaliana* individuals took place during spring in two consecutive years in 2022 and 2023 within the city limits of Düsseldorf (217 km^2^), which is a medium‐sized city with approximately 630,000 inhabitants and the capital of the German state of North Rhine‐Westphalia. The nine sampling locations were chosen to represent different habitats and areas of the city (map in Figure 2A, geographic locations in Table S3, photos of sampling sites in Figure S1). Each location was visited and sampled between three to six times and data on up to five *A. thaliana* individuals obtained at each location.

### 5.2 Field Sampling

At each site, the five tallest *A. thaliana* individuals were assessed and the phenotypical data collected from each individual plant according to Figure 2B. The rationale for choosing the tallest individuals (not smallest, average or median) is as follows: The tallest plants may represent the highest fitness level. Due to their advanced development, there are more chances for reproductive parameter assessment. There may be less signal‐to‐noise ratio when consistently using the tallest at various sites. Especially in a school project, it is much easier for students to select the tallest rather than the smallest individual or calculate and search for medium‐ and median‐sized plants. During 2022 and 2023, these were the number of siliques (> 2 cm), length of the longest silique, and the diameter of the rosette (calculated from the mean of three measurements displaced by 60° each time; see Figure 2B). During 2023, the following additional phenological data were collected: length of the main stem (stretched out manually), number of flowering side branches (stems branching off the main stem with at least one leaf ≥ 10 mm in length), and number of rosette leaves (≥ 10 mm length).

Individual plants which had developed at least one silique were used for plotting plant growth over time (common starting point).

Fresh plant material (usually leaves or flowers) from every individual was placed into labeled 1.5 mL plastic tubes for later laboratory analyses.

### 5.3 Plant Genotyping by PCR‐Sequencing

The plant samples were stored at −20°C until further use. The plant genotyping pipeline is explained in detail in the accompanying script (Texts S4 and S5 and summarized in a workflow, Figure 3A). Briefly, plant gDNA was prepared. Genomic *FRIGIDA* (*FRI*)‐specific PCR‐fragments were obtained using modified procedures described by Johanson et al. 2000 and Corre 2005 with primers G/E site_F and 16‐bp_R, sequenced and genetic variation assessed following sequence comparisons using standard DNA sequence analysis software tools available through the National Center for Biotechnology Information, NCBI (Sayers et al. 2025), and the molecular biology cloud platform Benchling (Benchling 2025). The *Arabidopsis* gene ID of *FRI* is At4g00650. For comparison, the *A. thaliana* Col‐0 standard reference sequence was used (Berardini et al. 2015).

### 5.4 Statistics

For the statistical analysis, the programs R (R Core Team 2025) and IBM SPSS Statistics (IBM Corp 2020) were used. Descriptive statistics involved the usual calculation of arithmetic mean ±1 standard deviation, median, and range. We used R and SPSS for all statistical analyses. First, we used the parametric linear regression model to test for a correlation between an independent continuous variable and a dependent continuous variable. Since many of these variables were right‐skewed, we used the Box‐Cox transformation (Krebs 1999; Wessa 2016) when needed which caused all the transformed variables to exhibit a normal distribution (tested by the Shapiro‐Wilk test, all variables had *p* > 0.05 after transformation). To show alternative models to the (1) linear regression model, we also used the (2) second‐degree polynomial model, (3) the exponential fit model and (4) the sigmoidal fit model for illustrative purposes (Figure S2). The best model fit was evaluated by the calculation of the AICc score for each model (Burnham and Anderson 2002). In some cases, we used the nonparametric Spearman‐rank correlation to test for a correlation between two variables. Finally, we used the nonparametric Mann‐Whitney *U* test to test for differences between two unpaired groups. All *p*‐values are two‐tailed, and a level of *p* < 0.05 was considered statistically significant.

### 5.5 Adaption of PUKI Methodology for Secondary School Projects

From the beginning of the PUKI project, a concerted effort was made to adapt the methodology developed for the participation of citizen scientists and school classes by taking into account feedback from teachers and instructors of secondary schools (Texts S1–S6). The results of these efforts are presented in the form of a school laboratory manual (Texts S4 and S5). This manual was tested during local outreach science communication events for children, such as KölnerKinderUniversität (KKU, https://kinderuni.uni‐koeln.de/), Nacht der Wissenschaft (“Night of Science,” https://www.nachtderwissenschaft‐duesseldorf.de/) and an online lecture (Melanchthon‐Schule, participants aged 14–18) (Figure 5). The resulting pipeline for the obtention and analysis of plant phenotypic and genetic data mirrors real laboratory work and has been adapted for school purposes, including study materials such as exemplary datasets and outcomes of the here‐presented study (Datasets S1 and S2, Texts S1–S6).

## Author Contributions

**Bruno Andreas Walther:** conceptualization (equal), data curation (equal), formal analysis (equal), investigation (equal), methodology (equal), supervision (equal), visualization (equal), writing – original draft (equal). **Leonard Jakob Klamann:** formal analysis (equal), investigation (equal), methodology (equal), writing – review and editing (equal). **Sarah Victoria Weirauch:** formal analysis (equal), investigation (equal), methodology (equal), writing – review and editing (equal). **Ghinwa Mashhady:** formal analysis (equal), funding acquisition (equal), investigation (equal), methodology (equal), visualization (equal), writing – original draft (equal), writing – review and editing (equal). **Hans‐Jörg Mai:** formal analysis (supporting), visualization (supporting). **Petra Bauer:** conceptualization (equal), data curation (equal), funding acquisition (lead), supervision (equal), writing – review and editing (equal).

## Funding

This work was supported by Avicenna Studienwerk, Fellowship (G.M.), Deutsche Forschungsgemeinschaft, Project‐ID 456082119—TRR 341 “Plant Ecological Genetics” project Z5 (P.B.).

## Disclosure

During the preparation of this work the authors used [Chat GPT, version 5.1] to improve the language and readability of the manuscript. After using this tool, the authors reviewed and edited the content as needed and take full responsibility for the content of the publication. The authors used this tool to draft and restructure parts of the Introduction and Discussion. All AI‐generated text was checked, revised by the authors, and verified for accuracy; the authors take full responsibility for the content of the publication. No AI tool was used to generate or interpret data.

Accession Number: *FRIGIDA*, *FRI*, At4g00650.

## Conflicts of Interest

The authors declare no conflicts of interest.

## Supporting information


**Dataset S1:** Plant phenotyping data from 2022 and 2023.

**Dataset S2:** *FRI* sequences for analysis, FASTA format.

**Figure S1:** Photos of the 9 locations where *A. thaliana* individuals were sampled in Düsseldorf in 2022 and 2023. For additional information on sampling sites see Figure 2A; Table S3 for geographic coordinates and details. Location 1. Small patch of grass next to roadside tree surrounded by road and pedestrian walkway. In 2022, ≤ 5 *A. thaliana* individuals grew here (photo by L. Klamann). Location 2. Along both roadsides of a small, paved street going through allotment gardens. In 2022, > 30 *A. thaliana* individuals grew here (photo by B. A. Walther). Location 3. Cobbled pavement near a house façade with overhanging roof. In 2022, ≤ 5 *A. thaliana* individuals grew here (photo by L. Klamann). Location 4. Tree bed on public walkway made of gravel. In 2022, 11–30 *A. thaliana* individuals grew here, but none in 2023 (photo by S. Weirauch). Location 5. Sloped meadow stretching along the Rhine River. In 2022, 12 *A. thaliana* individuals grew here (photo by L. Klamann). Location 6. Small patch of grass next to the small inner‐city street. In 2022, 6–10 *A. thaliana* individuals grew here, but none in 2023 (photo by B. A. Walther). Location 7. Pavement stones of this emergency access road. In 2022, 11–30 *A. thaliana* individuals grew here (photo by B. A. Walther).

**Figure S2:** Best‐fitting models of growth curves based on shoot height. Four different models (A–D) fit to the same data gathered from 133 plant individuals in 2023. The horizontal axis shows the time in weeks, whereby the first week of growth corresponds to the stage with at least one silique (see Methods for definition). The vertical axis shows the length of the individual measured in millimeters (mm); the median data value is indicated by an orange dot. The four models all fit the data reasonably well and are all statistically significant. However, the best explanation of the data is given by the sigmoidal fit (also called the S‐shaped plant growth curve) because it is associated with the lowest AICc. AICc, Akaike information criterion corrected for small sample sizes; RSS, residual sum of squares.

**Table S1:** Important scientific skills which secondary school students need to learn during science lessons.

**Table S2:** Important concepts, terms and techniques required to understand genetic research.

**Table S3:** Nine locations where *A. thaliana* individuals were sampled in Düsseldorf.

**Table S4:** Simple linear regression analyses of morphological characteristics (or variables) of *A. thaliana* individuals measured at seven locations in Düsseldorf in 2023.

**Text S1:** Why choose biology and ecology for a school project?
**Text S2:** Necessary pre‐knowledge about science and plant ecological genetics.
Prerequisite 1: Skills of scientific work.
Prerequisite 2: Principles of genetics in an ecological context including evolution and selection of genetic variants in the environment.
Prerequisite 3: Principles of botany applicable to understanding plant growth and its requirements.
Background Module S1: Background knowledge about the biology, ecology and genetics of the thale cress.
Background Module S2: Why do plants scientists work with the thale cress?
Background Module S3: How does the thale cress deal with environmental challenges in ecological settings?
**Text S3:** Three examples of how to analyze and discuss statistical outcomes.
Background Module S4: Understanding and exploring correlations.
Background Module S5: Comparing two groups.
Background Module S6: What model explains my data best?
**Text S4:** German text for the school project about laboratory techniques and analyses for genotyping by student classes in secondary schools.
**Text S5:** English text for the school project about laboratory techniques and analyses for genotyping by student classes in secondary schools.
**Text S6:** Examples of illustrating texts and videos useful for teaching the basic genetic concepts and techniques used in this school project as well as free statistical resources.

## Data Availability

All original data (collection sites, phenotyping data, *FRI* gene sequencing data) are provided in the form of tables, graphs, and in supplemental files.
