# Supplementary material for: A Research Project for Secondary Schools on Urban Plant Ecological Genetics
Source: Ecol Evol. 2026 Oct 18;16(10):e74482. doi: 10.1002/ece3.74482 (PMC13678418; doi:10.1002/ece3.74482)
Supplement: Supplementary file 1 [file ECE3-16-e74482-s001.pdf]

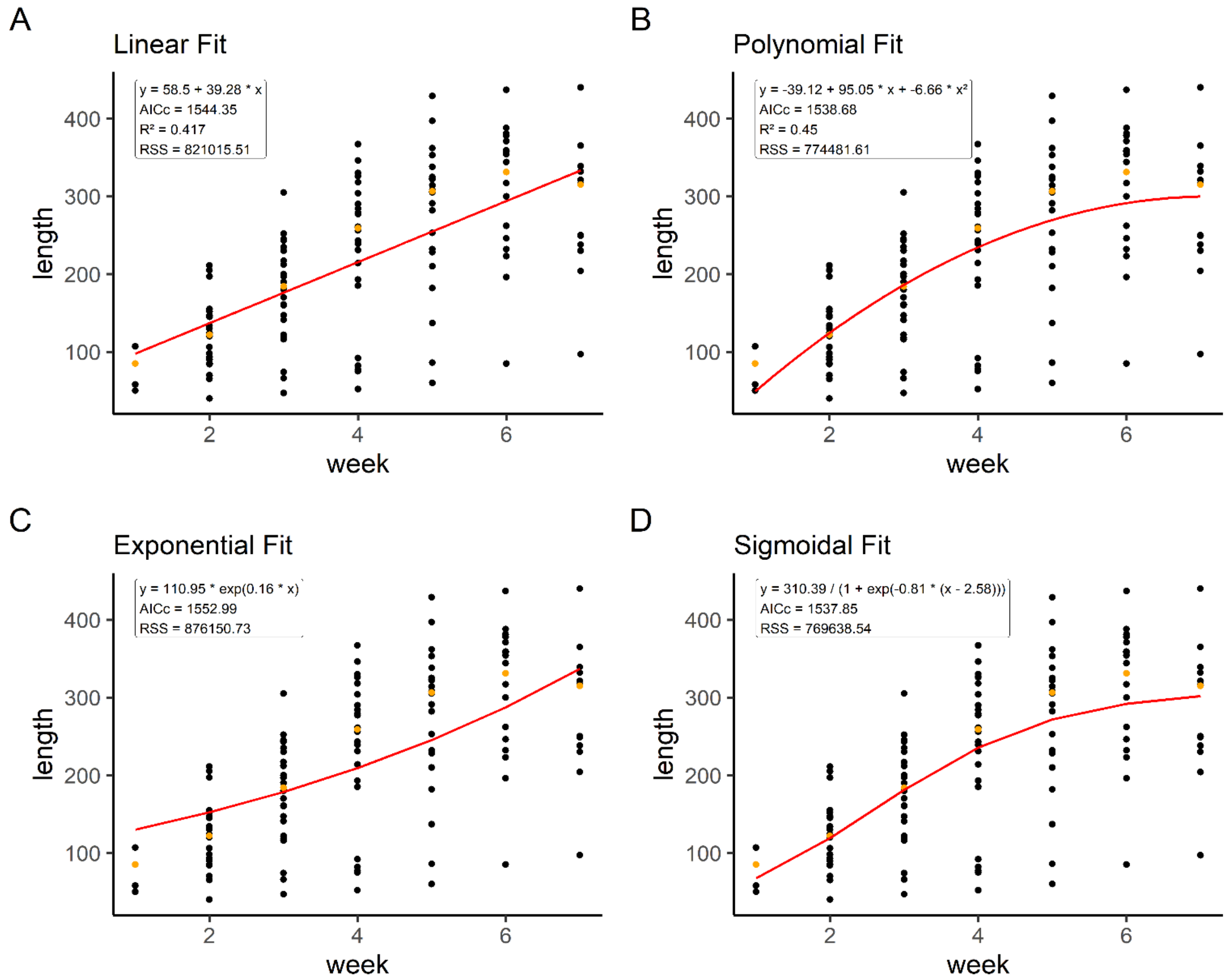

Figure S2. Best-fitting models of growth curves based on shoot height. Four different models (A-D) fit to the same data gathered from 133 plant individuals in 2023. The horizontal axis shows the time in weeks, whereby the first week of growth corresponds to the stage with at least one silique (see Methods for definition). The vertical axis shows the length of the individual measured in millimetres (mm); the median data value is indicated by an orange dot. The four models all fit the data reasonably well and are all statistically significant. However, the best explanation of the data is given by the sigmoidal fit (also called the S-shaped plant growth curve) because it is associated with the lowest AICc. AICc = Akaike information criterion corrected for small sample sizes; RSS = residual sum of squares.
