# Supplementary material for: A Research Project for Secondary Schools on Urban Plant Ecological Genetics
Source: Ecol Evol. 2026 Oct 18;16(10):e74482. doi: 10.1002/ece3.74482 (PMC13678418; doi:10.1002/ece3.74482)
Supplement: Supplementary file 2 [file ECE3-16-e74482-s002.pdf]

Table S2. Important concepts, terms and techniques required to understand genetic research at the level of the working projects presented here.

Concepts and techniques	Definitions and some examples
Concepts and terms	
Genetic code	The genetic code is the biological language written with sequences of four chemical "letters" (A, T, C, and G); the sequence of letters on the DNA provides the instructions to build proteins, the essential building blocks of life.
Deoxyribonucleic acid (DNA)	DNA is the very large molecule that carries the genetic instructions for most living organisms; it is structured as a double helix containing the sequence of letters A, T, C, and G, which make up the blueprints for building and maintaining the body.
Ribonucleic acid (RNA)	RNA is a molecule that reads the genetic information of the DNA and then guides the synthesis of proteins, acting as a messenger and sometimes a builder.
Amino acids	Amino acids are the building blocks of proteins, linked together in specific sequences to form the diverse array of proteins necessary for life.
Base pairs	Base pairs (bp) are the fundamental building blocks of DNA and RNA, forming the rungs of the double-helix structure in DNA or the complementary strands of RNA. They are specific pairs of nitrogenous bases held together by hydrogen bonds, which ensure the stable yet flexible structure of the genetic material. In DNA, each base pair consists of either (1) adenine (abbreviated as letter A) and thymine (T) or cytosine (C) and guanine (G), thus forming the base pairs A-T or C-G. In RNA, thymine (T) is replaced by uracil (U) so that the base pairs become A-U and C-G. Base pairs are measured in units of base pairs (bp) for length, such as kilobases (kb, 1000 bp) or megabases (Mb, 1000000 bp), to describe the size of genomes or DNA fragments.
Proteins	Proteins are complex molecules that perform a wide range of functions in the body, from building tissues to catalyzing biochemical reactions, all crafted from chains of amino acids.
Transcription and translation	Transcription is the process where a segment of DNA is copied into RNA, while translation is when the RNA sequence is used to build proteins, together forming the pathway from genetic code to functional molecules.
Chromosomes	Chromosomes are long, organized structures of DNA and proteins found in cells, containing many genes and ensuring the accurate distribution of genetic material during cell division. E.g., humans have 46 chromosomes, arranged in 23 pairs, but pea plants have only 14 chromosomes, arranged in 7 pairs.
Cells	Cells are the basic structural and functional units of all living organisms, each enclosed by a membrane and containing the machinery needed for life processes, including the entire DNA.
Mutations and heredity	Mutations are changes in the DNA sequence that can be passed from one generation to the next, influencing traits and contributing to genetic diversity (e.g., variation in the ability to deal with cold or hot climates).
Gene regulation	Gene regulation is the process by which cells control which genes are turned on or off, ensuring that the right proteins are made at the right time and place; e.g., different genes turn

	on during cold and hot periods.
Genotype	The genotype is the genetic makeup of an organism, consisting of the specific sequences of DNA that code for traits. The genotype interacts with environmental conditions to create the phenotype.
Phenotype	The phenotype is the observable physical and functional traits of an organism, such as height, flower or fruit colour, and enzyme activity, shaped by both genetic and environmental factors.
Environmental conditions	Environmental conditions include all the external conditions and influences that affect the growth, development, and survival of organisms. Typical conditions important for plants are light, weather and climate, soil, and exposure to chemicals, herbivores, and diseases.
Plasticity	Plasticity refers to the ability of an organism to change and adapt its phenotype in response to environmental conditions, showcasing the flexibility of biological systems; e.g., the same plant genotype might result in a small plant where little water and nutrients are in the soil, but in a large plant where lots of water and nutrients are in the soil.
Techniques	
Centrifugation	Centrifugation is a technique used to separate substances in a mixture by spinning them at high speeds, causing components to separate based on their density; used to extract, e.g., DNA from plant leaf samples.
Pipetting	Pipetting is a common laboratory technique used to measure and transfer small volumes of liquid accurately using a specialized tool called a pipette.
Genotyping	Genotyping is the process of determining the genetic makeup of an individual by examining its DNA sequence, often used to identify genetic variations linked to specific traits or diseases.
Polymerase chain reaction	PCR is a powerful technique used to amplify small segments of DNA, generating billions of copies for analysis in research, forensic science, and medical diagnostics.
Gel electrophoresis	Gel electrophoresis is a method for separating DNA, RNA, or proteins based on their size and charge by applying an electric current to a gel matrix. Larger molecules travel slower through the gel than smaller molecules.
Sequencing	Sequencing is the process of determining the exact order of nucleotides in a DNA or RNA molecule, providing detailed information about genetic information and variations. In essence, it is reading the letters A, T, C, and G of the genetic code one by one.
