# Supplementary material for: A Research Project for Secondary Schools on Urban Plant Ecological Genetics
Source: Ecol Evol. 2026 Oct 18;16(10):e74482. doi: 10.1002/ece3.74482 (PMC13678418; doi:10.1002/ece3.74482)
Supplement: Supplementary file 3 [file ECE3-16-e74482-s003.pdf]

```
>Col-0 FRIGIDA
GAGTCAATCGAAAACGCAATTGATTCCAAACTCGAGAGTAACGGCGTTGTCCTCGCCGCGCGGAACAA
TAATTTCCATCAGCCGATGTTATCGCCTCCGCGGAACAATGTATCTGTAGAAACCACCGTCACTGTGA
GCCAACCGTCTCAGGAGATTGTACCGGAGACGTCGAATAAACCGGAGGGGGAACGTATATGTGAGTTG
ATGTGTAGCAAAGGTCTGCGTAAATACATATACGCGAATATCTCTGATCAAGCTAAGTTAATGGAAGA
GATTCCTTCAGCTTTGAAATTGGCCAAGGAGCCAGCGAAGTTTGTATTGGATTGTATTGGCAAGTTTT
ACTTACAAGGGCGTAGAGCATTTACTAAAGAGTCGCCTATGAGCTCTGCGAGACAAGTTTCGCTTCTT
ATACTGGAGTCTTTTCTTCTAATGCCTGATCGTGGTAAAGGGAAGGTGAAGATTGAGAGTTGGATTAA
AGATGAGGCGGAGACGGCTGCTGTTGCTTGGAGGAAAAGGTTGATGACTGAAGGAGGATTAGCTGCGG
CTGAGAAAATGGATGCAAGGGGTTTGCTTTTACTAGTTGCTTGTTTTGGTGTTCCTTCAAACTTTAGG
AGTACAGATTTGCTGGATTTGATAAGGATGAGTGGTTCGAATGAGATTGCCGGTGCTTTGAAGCGGTC
ACAGTT----------------TCAGGTACCATATTCTGTTCTCACTCGGTG
>Allele I
GAGTCAATCGAAAACGCAATTGATTCCAAACTCGAGAGTAACGGCGTTGTCCTCGCCGCGCGGAACAA
TAATTTCCATCAGCCGATGTTATCGCATCCGCGGAACAATGTATCTGTAGAAACCACCGTCACTGTGA
GCCAACCGTCTCAGGAGATTGTACCGGAGACGTCGAATAAACCGGAGGGGGGACGTATGTGTGAGTTG
ATGTGTAGCAAAGGTCTGCGTAAATACATATACGCGAATATCTCTGATCAAGCTAAGTTAATGGAAGA
GATTCCTTCAGCTTTGAAATTGGCCAAGGAGCCAGCGAAGTTTGTATTGGATTGTATTGGCAAGTTTT
ACTTACAAGGGCGTAGAGCATTTACTAAAGAGTCGCCTATGAGCTCTGCGAGACAAGTTTCGCTTCTT
ATACTGGAGTCTTTTCTTCTAATGCCTGATCGTGGTAAAGGGAAGGTGAAGATTGAGAGTTGGATTAA
AGATGAGGCGGAGACGGCTGCTGTTGCTTGGAGGAAAAGGTTGATGACTGAAGGAGGATTAGCTGCGG
CTGAGAAAATGGATGCAAGGGGTTTGCTTTTACTAGTTGCTTGTTTTGGTGTTCCTTCAAACTTTAGG
AGTACAGATTTGCTGGATTTGATAAGGATGAGTGGTTCGAATGAGATTGCCGGTGCTTTGAAGCGGTC
ACAGTTTCTTGTCCCTATGGTCTCAGGTACCATATTCTGTTCTCACTCGGTG
>Allele II
GAGTCAATCGAAAACGCAATTGATTCCAAACTCGAGAGTAACGGCGTTGTCCTCGCCGCGCGGAACAA
TAATTTCCATCAGCCGATGTTATCGCCTCCGCGGAACAATGTATCTGTAGAAACCACCGTCACTGTGA
GCCAACCGTCTCAGGAGATTGTACCGGAGACGTCGAATAAACCGGAGGGGGGACGTATGTGTGAGTTG
ATGTGTAGCAAAGGTCTGCGTAAATACATATACGCGAATATCTCTGATCAAGCTAAGTTAATGGAAGA
GATTCCTTCAGCTTTGAAATTGGCCAAGGAGCCAGCGAAGTTTGTATTGGATTGTATTGGCAAGTTTT
ACTTACAAGGGCGTAGAGCATTTACTAAAGAGTCGCCTATGAGCTCTGCGAGACAAGTTTCGCTTCTT
ATACTGGAGTCTTTTCTTCTAATGCCTGATCGTGGTAAAGGGAAGGTGAAGATTGAGAGTTGGATTAA
AGATGAGGCGGAGACGGCTGCTGTTGCTTGGAGGAAAAGGTTGATGACTGAAGGAGGATTAGCTGCGG
CTGAGAAAATGGATGCAAGGGGTTTGCTTTTACTAGTTGCTTGTTTTGGTGTTCCTTCAAACTTTAGG
AGTACAGATTTGCTGGATTTGATAAGGATGAGTGGTTCGAATGAGATTGCCGGTGCTTTGAAGCGGTC
ACAGTTTCTTGTCCCTATGGTCTCAGGTACCATATTCTGTTCTCACTCGGTG
>Allele III
GAGTCAATCGAAAACGCAATTGATTCCAAACTCGAGAGTAACGGCGTTGTCCTCGCCGCGCGGAACAA
TAATTTCCATCAGCCGATGTTATCGCCTCCGCGGAACAATGTATCTGTAGAAACCACCGTCACTGTGA
GCCAACCGTCTCAGGAGATTGTACCGGAGACGTCGAATAAACCGGAGGGGGGACGTATGTGTGAGTTG
ATGTGTAGCAAAGGTCTGCGTAAATACATATACGCGAATATCTCTGAACAAGCTAAGTTAATGGAAGA
GATTCCTTCAGCTTTGAAATTGGCCAAGGAGCCAGCGAAGTTTGTATTGGATTGTATTGGCAAGTTTT
ACTTACAAGGGCGTAGAGCATTTACTAAAGAGTCGCCTATGAGCTCTGCGAGACAAGTTTCGCTTCTT
ATACTGGAGTCTTTTCTTCTAATGCCTGATCGTGGTAAAGGGAAGGTGAAGATTGAGAGTTGGATTAA
AGATGAGGCGGAGACGGCTGCTGTTGCTTGGAGGAAAAGGTTGATGACTGAAGGAGGATTAGCTGCGG
CTGAGAAAATGGATGCAAGGGGTTTGCTTTTACTAGTTGCTTGTTTTGGTGTTCCTTCAAACTTTAGG
AGTACAGATTTGCTGGATTTGATAAGGATGAGTGGTTCGAATGAGATTGCCGGTGCTTTGAAGCGGTC
ACAGTTTCTTGTCCCTATGGTCTCAGGTACCATATTCTGTTCTCACTCGGTG
>Allele  IV
GAGTCAATCGAAAACGCAATTGATTCCAAACTCGAGAGTAACGGCGTTGTCCTCGCCGCGCGGAACAA
TAATTTCCATCAGCCGATGTTATCGCCTCCGCGGAACAATGTATCTGTAGAAACCACCGTCACTGTGA
GCCAACCGTCTCAGGAGATTGTACCGGAGACGTCGAATAAACCGGAGGGGGAACGTATGTGTGAGTTG
ATGTGTAGCAAAGGTCTGCGTAAATACATATACGCGAATATCTCTGATCAAGCTAAGTTAATGGAAGA
GATTCCTTCAGCTTTGAAATTGGCCAAGGAGCCAGCGAAGTTTGTATTGGATTGTATTGGCAAGTTTT
```

ACTTACAAGGGCGTAGAGCATTTACTAAAGAGTCGCCTATGAGCTCTGCGAGACAAGTTTCGCTTCTT
ATACTGGAGTCTTTTCTTCTAATGCCTGATCGTGGTAAAGGGAAGGTGAAGATTGAGAGTTGGATTAA
AGATGAGGCGGAGACGGCTGCTGTTGCTTGGAGGAAAAGGTTGATGACTGAAGGAGGATTAGCTGCGG
CTGAGAAAATGGATGCAAGGGGTTTGCTTTTACTAGTTGCTTGTTTTGGTGTTCCTTCAAACTTTAGG
AGTACAGATTTGCTGGATTTGATAAGGATGAGTGGTTCGAATGAGATTGCCGGTGCTTTGAAGCGGTC
ACAGTTTCTTGTCCCTATGGTCTCAGGTACCATATTCTGTTCTCACTCGGTG
>Allele V
GAGTCAATCGAAAACGCAATTGATTCCAAACTCGAGAGTAACGGCGTTGTCCTCGCCGCGCGGAACAA
TAATTTCCATCAGCCGATGTTATCGCCTCCGCGGAACAATGTATCTGTAGAAACCACCGTCACTGTGA
GCCAACCGTCTCAGGAGATTGTACCGGAGACGTCGAATAAACCGGAGGGGGAACGTATATGTGAGTTG
ATGTGTAGCAAAGGTCTGCGTAAATACATATACGCGAATATCTCTGATCAAGCTAAGTTAATGGAAGA
GATTCCTTCAGCTTTGAAATTGGCCAAGGAGCCAGCGAAGTTTGTATTGGATTGTATTGGCAAGTTTT
ACTTACAAGGGCGTAGAGCATTTACTAAAGAGTCGCCTATGAGCTCTGCGAGACAAGTTTCGCTTCTT
ATACTGGAGTCTTTTCTTCTAATGCCTGATCGTGGTAAAGGGAAGGTGAAGATTGAGAGTTGGATTAA
AGATGAGGCGGAGACGGCTGCTGTTGCTTGGAGGAAAAGGTTGATGACTGAAGGAGGATTAGCTGCGG
CTGAGAAAATGGATGCAAGGGGTTTGCTTTTACTAGTTGCTTGTTTTGGTGTTCCTTCAAACTTTAGG
AGTACAGATTTGCTGGATTTGATAAGGATGAGTGGTTCGAATGAGATTGCCGGTGCTTTGAAGCGGTC
ACAGTT----------------TCAGGTACCATATTCTGTTCTCACTCGGTG

Protein translations of FRIGIDA (Col-0) and alleles I–V (same reading frame, rf1)

>rf 1 Col-0 FRIGIDA
ESIENAIDSKLESNGVVLAARNNNFHQPMLSPPRNNVSVETTVTVSQPSQEIVPETSNKP
EGERICELMCSKGLRKYIYANISDQAKLMEEIPSALKLAKEPAKFVLDCIGKFYLQGRRA
FTKESPMSSARQVSLLILESFLLMPDRGKGKVKIESWIKDEAETAAVAWRKRLMTEGGLA
AAEKMDARGLLLLVACFGVPSNFRSTDLLDLIRMSGSNEIAGALKRSQFQVPYSVLTR

>rf 1 Allele I
ESIENAIDSKLESNGVVLAARNNNFHQPMLSHPRNNVSVETTVTVSQPSQEIVPETSNKP
EGGRMCELMCSKGLRKYIYANISDQAKLMEEIPSALKLAKEPAKFVLDCIGKFYLQGRRA
FTKESPMSSARQVSLLILESFLLMPDRGKGKVKIESWIKDEAETAAVAWRKRLMTEGGLA
AAEKMDARGLLLLVACFGVPSNFRSTDLLDLIRMSGSNEIAGALKRSQFLVPMVSGTIFC
SHSV

>rf 1 Allele II
ESIENAIDSKLESNGVVLAARNNNFHQPMLSPPRNNVSVETTVTVSQPSQEIVPETSNKP
EGGRMCELMCSKGLRKYIYANISDQAKLMEEIPSALKLAKEPAKFVLDCIGKFYLQGRRA
FTKESPMSSARQVSLLILESFLLMPDRGKGKVKIESWIKDEAETAAVAWRKRLMTEGGLA
AAEKMDARGLLLLVACFGVPSNFRSTDLLDLIRMSGSNEIAGALKRSQFLVPMVSGTIFC
SHSV

>rf 1 Allele III
ESIENAIDSKLESNGVVLAARNNNFHQPMLSPPRNNVSVETTVTVSQPSQEIVPETSNKP
EGGRMCELMCSKGLRKYIYANISEQAKLMEEIPSALKLAKEPAKFVLDCIGKFYLQGRRA
FTKESPMSSARQVSLLILESFLLMPDRGKGKVKIESWIKDEAETAAVAWRKRLMTEGGLA
AAEKMDARGLLLLVACFGVPSNFRSTDLLDLIRMSGSNEIAGALKRSQFLVPMVSGTIFC
SHSV

>rf 1 Allele IV
ESIENAIDSKLESNGVVLAARNNNFHQPMLSPPRNNVSVETTVTVSQPSQEIVPETSNKP
EGERMCELMCSKGLRKYIYANISDQAKLMEEIPSALKLAKEPAKFVLDCIGKFYLQGRRA
FTKESPMSSARQVSLLILESFLLMPDRGKGKVKIESWIKDEAETAAVAWRKRLMTEGGLA
AAEKMDARGLLLLVACFGVPSNFRSTDLLDLIRMSGSNEIAGALKRSQFLVPMVSGTIFC
SHSV

>rf 1 Allele V
ESIENAIDSKLESNGVVLAARNNNFHQPMLSPPRNNVSVETTVTVSQPSQEIVPETSNKP
EGERICELMCSKGLRKYIYANISDQAKLMEEIPSALKLAKEPAKFVLDCIGKFYLQGRRA
FTKESPMSSARQVSLLILESFLLMPDRGKGKVKIESWIKDEAETAAVAWRKRLMTEGGLA
AAEKMDARGLLLLVACFGVPSNFRSTDLLDLIRMSGSNEIAGALKRSQFQVPYSVLTR
