# Supplementary material for: A Research Project for Secondary Schools on Urban Plant Ecological Genetics
Source: Ecol Evol. 2026 Oct 18;16(10):e74482. doi: 10.1002/ece3.74482 (PMC13678418; doi:10.1002/ece3.74482)
Supplement: Supplementary file 4 [file ECE3-16-e74482-s004.pdf]

Supplemental file

to

A research project for secondary schools on urban plant ecological genetics

Bruno Andreas Walther[1,*], Leonard Jakob Klamann[1], Sarah Victoria Weirauch[1], Ghinwa Mashhady[1], Hans-Jörg Mai[1], Petra Bauer[1,2,*]

[1]Institut für Botanik, Heinrich Heine Universität Düsseldorf, Universitätsstraße 1, D-40225 Düsseldorf, Germany

[2]Cluster of Excellence on Plant Science (CEPLAS), Heinrich-Heine-University, 40225 Düsseldorf, Germany

*Corresponding authors: Bruno.Walther@hhu.de, Petra.Bauer@hhu.de

Contents of supplemental file:

Text S1: Why choose biology and ecology for a school project?

Text S2: Necessary pre-knowledge about science and plant ecological genetics

Prerequisite 1: Skills of scientific work

Prerequisite 2: Principles of genetics in an ecological context including evolution and selection of genetic variants in the environment

Prerequisite 3: Principles of botany applicable to understanding plant growth and its requirements

Background Module S1: Background knowledge about the biology, ecology and genetics of the thale cress.

Background Module S2: Why do plants scientists work with the thale cress?

Background Module S3: How does the thale cress deal with environmental challenges in ecological settings?

Text S3: Three examples of how to analyse and discuss statistical outcomes

Background Module S4: Understanding and exploring correlations.

Background Module S5: Comparing two groups.

Background Module S6: What model explains my data best?

Text S4. German text for the school project about laboratory techniques and analyses for genotyping by student classes in secondary schools.

Text S5. English text for the school project about laboratory techniques and analyses for genotyping by student classes in secondary schools.

Text S6. Examples of illustrating texts and videos useful for teaching the basic genetic concepts and techniques used in this school project as well as free statistical resources.

Supplement References

Text S1: Why choose biology and ecology for a school project?

Naturally, school projects and citizen science projects can cover various disciplines. Projects with a biological focus have, however, some advantages:

1. Biology projects lend themselves to *being outside* and *experiencing nature*. While biology can also happen in the classroom and laboratory, the possibility of taking field trips into nature is an exciting alternative for most students who usually spend much time sitting in the classroom. Being in nature was typically described by Finnish adolescents as improving their psychological well-being benefits, especially increasing calmness, and improving mood or positive feelings (Puhakka and Hakoköngäs, 2024). Finnish students also valued nature for its material, aesthetic, and recreational values, as well as its diversity and future value (Sjöblom and Wolff, 2017).

2. Many biology projects allow for *hands-on experiments or observations*, like growing plants, studying insects, or observing cells under a microscope. Biology projects can also implement place-based-research where the relevance comes from the direct connection to the surrounding environment. Projects can further tackle *real-world problems*, such as climate change, conservation, medicine, and agriculture. This makes it very interactive and engaging.

3. Biology helps us understand the processes of life, such as how plants grow, how our bodies function, and how diseases affect us. Therefore, it connects directly to health, food, the environment, and even how our own bodies work. Since everyday *relevance* is an essential element of student motivation, asking questions in this context is particularly motivating (Graf, 2016; Ballard et al. 2017). The 'dreaming up' of scientific questions helps young people to discover the nature of scientific research (Barnard et al. 2011; Symes et al. 2015; Lüsse et al. 2022). Especially in nature, observations which then lead to questions can be readily made by chance in a haphazard way or through more organized and systematic sampling protocols simply because nature is available almost everywhere, even within or near the school property (Schuttler et al. 2019; Soanes et al. 2020; Sieg and Dreesmann, 2021), in the middle of a city (Walther and Yang, 2024), or on the side of the road (Englefield et al. 2020; Hsu and Lin, 2023).

4. Further relevance can be added if projects help with *career* exploration. Biology is foundational to careers in medicine, environmental science, genetics, and bioengineering. By choosing a biology project, students can explore interests in these fields and get a taste of what a future career might look like (Flowers et al. 2016; van Eijck & Roth 2009).

5. Biology projects can be designed to *interface* with other natural sciences and even the social sciences and the arts. If we take plants as an example, an interdisciplinary research project could look like this: the plants can be examined in biology lessons, the soil in chemistry lessons, and the

climatic conditions in geography lessons. The idea and principles of statistics can be taught in mathematics. The environmental importance of plants in maintaining healthy ecosystems and human well-being is also of socio-economic importance (Millennium Ecosystem Assessment, 2005; Walther et al. 2016), and drawing and illustrating plants could be part of the artistic curriculum (Quillin and Thomas, 2015).

Overall, a well-designed biology project provides a unique mix of practical skills, real-world relevance, and natural curiosity that makes it an ideal subject for a school project. Before we present our school projects, which introduce some of the basics of plant genetics, we will focus on some necessary pre-knowledge which is essential to understand and appreciate the contents of our project.

Text S2: Necessary pre-knowledge about science and plant ecological genetics

The Plant Ecological Genetics school project developed by PUKI requires basic pre-existing knowledge on genetic principles, evolution, ecological constraints especially in urban environments, botanical insight on plant morphology and physiology and data science. Therefore, we here identify the necessary prerequisites for the successful understanding and realisation of the school projects.

Prerequisite 1: Skills of scientific work

In Table S1, we present a list of basic scientific skills which secondary school students need to learn during prior science lessons to successfully realize our school projects. A good summary of ten important pedagogical principles is also given by García-Carmona and Acevedo-Díaz (2018). These are: the principle of formulating questions, the principle of creativity and imagination, the principle of experimentation, the principle of procedural diversity, the principle of errors as opportunity, the principle of modelling, the principle of cooperation and teamwork, the principle of argumentation and discussion, the principle of communication, and the principle of evaluation. These scientific principles and skills should be incorporated into biology lessons whenever possible, especially by motivating students to come up with their own questions and ideas. It should of course be noted that (1) the quality and characteristics of these skills differ in each grade and (2) the work steps and timing of the planned experiments must therefore be adjusted. If students are introduced to scientific work at an early age, their interest in continuing to participate in research questions in the future or even in doing appropriate professional training in the natural sciences could be further increased. School projects, such as the one presented here, are especially helpful in learning to set up and test scientific hypotheses. We expect that most secondary school students have a good or at least sufficient understanding of these principles, although there will of course be large variations between different students, classes, and schools.

Prerequisite 2: Principles of genetics in an ecological context including evolution and selection of genetic variants in the environment

To understand and implement the school projects presented below, students should be familiar with basic genetic principles and be able to apply them to plant ecology. In particular, they should understand that an organism's genetic constitution, or genotype, is heritable and comprises all of its genes, and that this genotype contains the instructions that determine the organism's traits and characteristics. The phenotype, in turn, comprises the observable and measurable morphological and physiological characteristics of the organism. Ideally, students know that the genetic material is DNA and that the specific information of DNA in genes serves to build proteins important for the expression of phenotypes. Importantly, students should have an understanding that genetic information is transmitted from generation to generation, and that it is not static, but that naturally mutations and genetic changes occur. If these genetic changes are advantageous to organisms in their environments, genetic variants may provide better chances for individuals to survive, spread and establish new populations of organisms that display specific adaptations to the environments. Ideally, the students can connect DNA with genetic variation. In this case, they understand the basis of genetic variation, namely that differences in DNA sequences of specific genes in different populations of organisms can result in different functionalities of the encoded proteins, which may allow the different populations of organisms to thrive in their environment and reproduce with distinct characteristics. The students recognize that the processes leading to genetic changes and the resulting phenotypic outcomes are part of the process called evolution, which can occur over both short-time and long-time scales (Futuyma, 2014; Futuyma and Kirkpatrick, 2022). Furthermore, the students should master important skills to study DNA, such as the handling of buffers, detergents and pipetting. Table S2 summarizes various concepts, terms and techniques that students become familiar with when applying an ecological genetics research project.

Prerequisite 3: Principles of botany applicable to understanding plant growth and its requirements

Another prerequisite is to understand the basic biology of the organism, which in our case are the growth features of our model plant species of the Brassicaceae family, namely the thale cress *Arabidopsis thaliana*. First, we explain the background on the morphology and growth of *A. thaliana* (Supplemental File / Text S2/ Background Module S1, e.g., germination, leaves, vegetative versus reproductive growth, flowering and fruit setting; requirements for plant growth such as temperature, light, water and minerals). Thereafter, we describe the advantages of *A. thaliana* as genetic model plant species (Supplemental File / Text S2/ Background Module S2, e.g., accessibility of genome sequences and understanding of gene functions for growth and physiology) and provide three examples for environment adaptations well studied in *A. thaliana*, including flowering time (Supplemental File / Text S2/ Background Module S3). The students can either read the information (Supplemental File / Text S2/ Background Modules S1-S3) themselves, but preferably, teachers would decide on how to utilize them (in presentations, discussions, experiments, calculations, etc.). Students should be able to master basic skills to assess phenotypes, such as handling a ruler, dealing with quantitative data and calculations.

Once students sufficiently master the three prerequisites and are able to fulfil the tasks as required by citizens in the PUKI project, they can focus on two scientific questions related to the PUKI project: 1) Is there phenotypic variation in plant growth in the urban neighbourhood, and is there a correlation between phenotypes and environmental factors? 2) Is there genetic variation, and do genotypes correlate with specific environments?

Thus, students will study ecological genetics as a subfield of genetics and will be able to describe genetic variation and explain its role in the adaptation and evolution of organisms in natural environments. Students could examine how environmental factors, such as climate, habitat, and ecological interactions influence genetic composition and phenotypic traits within populations. An important learning outcome that students should be aware of is that phenotypes result not merely from a genotype, but from the interactions of a genotype with environmental factors. This means that traits such as morphology, development, and biochemical properties, are influenced by both genetic and environmental conditions, resulting in different phenotypes depending on the environment despite the individual plants having the same genetic makeup, which is a phenomenon known as plasticity. For example, two plants with identical genetic makeup (essentially, like human identical twins) growing up in very different environments, e.g., one growing at the optimal temperature and another at a suboptimal temperature, would of course end up growing at different rates. On the other

hand, two plants with different genes growing up in the exactly the same environment would also grow up differently.

Through such examples and actual experimentation, students should realize that the combination of genetic makeup and phenotypic plasticity determines plant survival and health in any given environment.

Background Module S1: Background knowledge about the biology, ecology and genetics of the thale cress

The thale cress (*Arabidopsis thaliana*) is a small flowering plant that has become one of the most important laboratory model organisms in plant research in the world. It is used to study the molecular physiology of plant development, metabolism, nutrition and photosynthesis as well as stress responses. It originates from Southern Europe (Koornneef and Meinke, 2010; Krämer, 2015), but today grows naturally in Europe, North America, Asia and North Africa, due to migration after the last ice age. *A. thaliana* is often found in urban environments. The thale cress belongs to the plant family Brassicaceae (also called the crucifers) which includes many important food plants such as various cabbage types, mustard and rapeseed.

Biology

The thale cress is small and inconspicuous, with a usual height of 10-25 cm (rarely 60 cm), and its white flower petals are only 2-4 mm in size (Figure 2B, Figure M1-1). The four white petals are arranged in a cross-like pattern. Accordingly, an alternative name for Brassicaceae is crucifers. Due to its delicate architecture and early spring flowering, it flowers and multiplies mostly unnoticed by people. It is also treated as weed, and many people will pull it out without a second thought. *A. thaliana* grows in isolation of other plants e.g., in sandy pavement joints and in between cobble stones, as well as in plant communities e.g., on rich meadows with grasses or together with mosses and other plants below bushes and trees.

Germinating plants will first grow a rosette of basal leaves (Figure 2B, Figure M1-1). A leaf rosette consists of whorls with individual leaves that are separated by very short internodes, so that the rosette has stunted appearance and grows close to the ground. The first rosette leaves are small, while the later formed rosette leaves are long. Each leaf has a proximal petiole and a distal blade. There is a high degree of plasticity that allows the rosette to have any size from roughly 1-10 cm in diameter. Later in the life cycle, once reproduction is progressing, an inflorescence stem emerges from the rosette (Figure 2B, Figure M1-1). The inflorescence stem forms narrow cauline leaves, separated by long internodes. The inflorescence will sequentially form flowers, the oldest at the bottom, the youngest on top, each sitting on a flower stem (Figure 2B, Figure M1-1). The inflorescence stem can remain a single stem, usually in poor growth conditions, but mostly produces flowering side branches at the nodes of cauline leaves. The flowers self-pollinate usually. After fertilization, the ovary develops and transforms into a fruit, while seeds develop inside. The fruits of Brassicaceae are called siliques, consisting of two carpels fused on either side. *A. thaliana* siliques

are long and very narrow and typically hold 20 up to 60 tiny seeds (only 0.2-0.6 mm). Each plant can produce thousands of seeds. Once the siliques and seeds are ripe and dried, siliques spring open and release seeds. *A. thaliana* is an annual plant species. It flowers and fruits once in its lifetime, then turns senescent (yellow-brown colour) and dies.

Ecology

The thale cress grows well from temperate to semi-arid climates and in disturbed habitats with diverse soil types (especially rocky and sandy). In addition, the thale cress exhibits a remarkable ability to cope with abiotic stresses, such as drought and cold temperatures. Therefore, it is found in diverse ecosystems, including loose rough grasslands and meadows, rocky terrains, walls, and often disturbed sites such as railway lines, waste grounds, parking lots and roadsides.

Thale cress is a pioneer species on open soil with differing composition and full sun light, which the rosette leaves can tolerate. The production of high seed numbers helps in spreading in such areas. Despite of that, it is also found in meadows in communities with grasses or mosses. However, it is not a typical species when the cover is dense (e.g., a dense grass cover) or in full shade (e.g., inside a forest).

Genetics

In the year 2000, the thale cress was the first plant species for which the entire genome was fully sequenced (Arabidopsis Genome Initiative, 2000). This scientific milestone has revolutionized plant science. Its genome consists of five chromosomes, around 27.000 genes, and approximately 135 million base pairs (or 135 Mb). This small genome size together with its short life cycle and easy cultivation make the thale cress particularly accessible and convenient for genetic studies in most labs. Many tools of genetic research have since been developed to study the functions of single genes or several genes in combination. These tools have facilitated breakthroughs in understanding developmental and physiological processes, plant mutation, regulation and signalling, as well as adaptation to the environment.

Furthermore, there is documented natural variation of the thale cress which allows studying adaptation to local environments (Supplemental File / Text S2/ Background Module S3). To capture this natural variation, plants are collected from one place at one time. Such a collection, e.g., of seeds or plants, is called an accession, and it thus captures the genotype of the species in that location and at that time point.

For the thale cress alone, several thousand accessions have already been collected (Provart et al., 2016) representing the genetic diversity in this species (e.g., Johanson et al., 2000; Shindo et al.,

2005; Schmitz et al., 2013). Phenotypic traits of the thale cress, such as flowering time, disease resistance, and tolerance against drought can differ considerably between populations with different genetic makeups (Kover and Schaal, 2002; Thieme et al., 2022), and the reasons for the genetic disparities can encompass phenomena like single-gene mutations, genomic rearrangements, migration and mixtures of different populations.

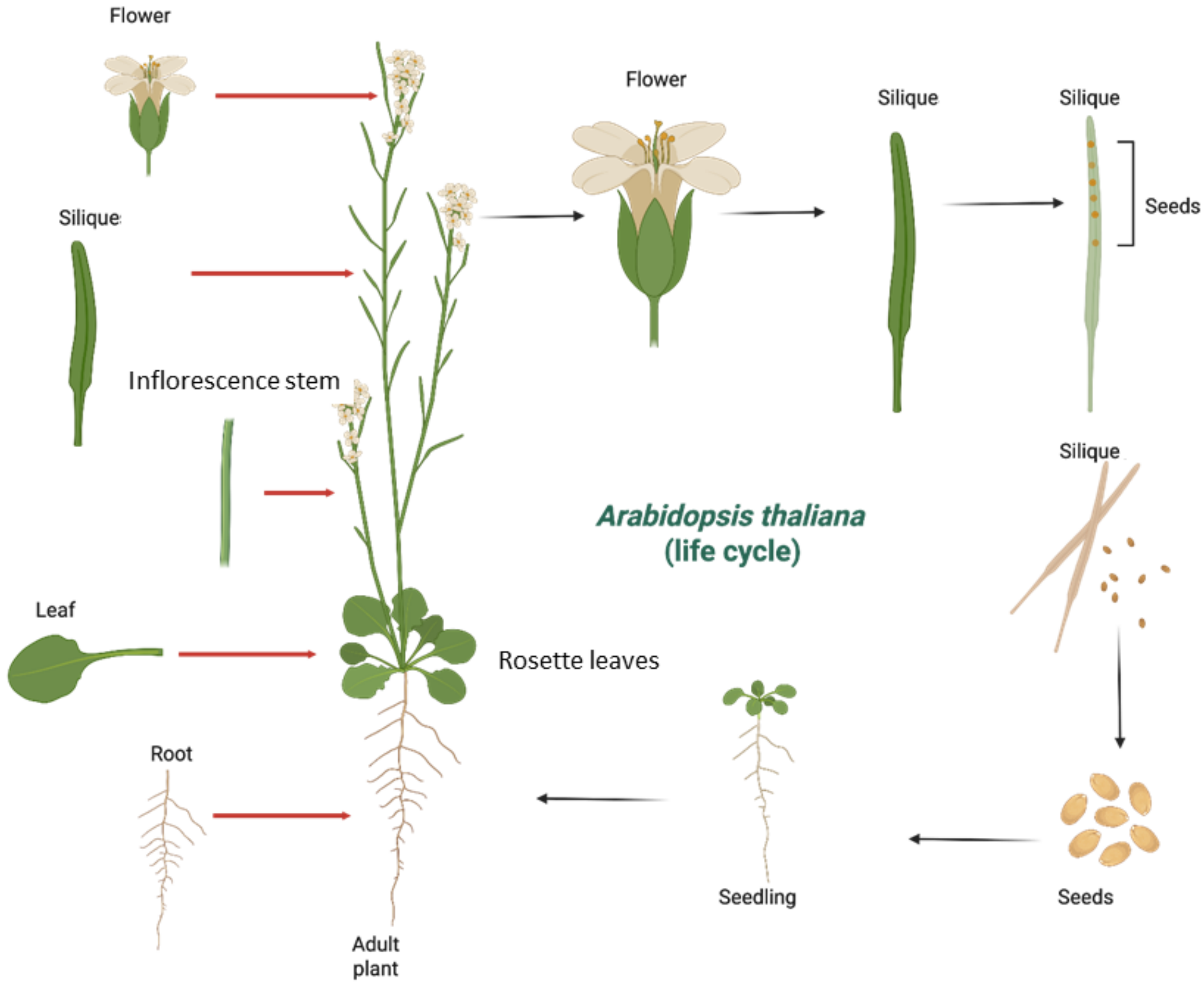

Figure M1-1. Morphological development of the thale cress *A. thaliana*. Leaf colour, length and shape, stem length, and number of stems, flowers and siliques can vary considerably based on growth conditions. Created with Biorender.

Background Module S2: Why do plants scientists work with the thale cress?

The thale cress grows in many parts of Europe, Asia and Africa. Its scientific name is *Arabidopsis thaliana*, and it is a model plant used by many plant scientists around the world. Many people consider it just a small weed, as it usually grows only to about 20-30 cm tall and is found in roadsides, railway lines, waste grounds, and other disturbed habitats (Supplemental File / Text S2/ Background Module S1).

So why is the thale cress so very popular with scientists all around the world? Why do the scientists use this plant species as a model organism for their scientific studies and to teach students about plant ecology, plant physiology and plant genetics?

Here are some reasons why scientists love to study this small plant:

1. The thale cress is diploid. The genetics of diploid species can be handled at ease applying the simple Mendelian genetics rules. Plant genetics provides many tools and techniques to understand the functional relevance of plant genes.
2. The thale cress grows quickly. Scientists multiply their plant materials on their own for controlled studies. *A. thaliana* grows seed to seed in just three months. This means scientists can obtain seeds for experiments and do plant experiments in reasonable time.
3. The thale cress requires little space to grow: Thale cress can be cultivated in small spaces even lighted cellar shelves and growth cabinets, which can be installed in many laboratories. There is no need for gardeners, greenhouses or field access.
4. The thale cress produces large quantities of seeds. Many seeds are required for physiological experiments to obtain reliable and reproducible data. Since a single *A. thaliana* plant can produce thousands of seeds, the availability of seeds is not a limitation.
5. The thale cress is a self-pollinating species. First, it requires no hand work to multiply a single plant, and second, an inbred line with little genetic variation can be obtained with a single plant. Such inbred lines allow obtaining reliable data on plant growth and physiology with little variation.
6. Importantly, among plants, the thale cress has a tiny genome with little genomic DNA (Supplemental File / Text S2/ Background Module S1). This made it possible to sequence the genome early and identify gene sequences.
7. Today, there are numerous tools and knowledge to conduct detailed molecular studies in the thale cress. Many of the tools have been generated with public funding under the permission to share them with the scientific community. These tools include databases on *A. thaliana* resources with world-wide access to genome sequence, genome annotation and interlinked

information on individual genes and proteins for easy access to structural, functional and literature data, e.g. provided by The Arabidopsis Information Resource (TAIR) at www.arabidopsis.org. A large collection of plant mutants, transgenic lines and accessions is available, and seeds are centrally provided by a stock centre. The literature on basic science about fundamental aspects of *A. thaliana* development and physiology is vast, which allows scientists to study processes in molecular detail by digging deep to uncover molecular mechanisms for cell signalling and connecting molecular cell physiology with plant growth and ecology. This helped greatly to generate new knowledge on the inner workings of plants at unprecedented scales, suited for high-quality publications.

Hence, the thale cress has become a reference to other plants. The basic principles of the molecular mechanisms determining plant development and physiology are conserved across flowering plant species. From *A. thaliana*, we can largely infer how other plant species, including crops, function genetically in different environments. For example, the molecular processes that enable the thale cress to absorb nutrients from the soil and develop flowers are similar to those found in many other plant species, including cultivated food and feed crops that are more difficult to study, such as wheat, rice, soybean, and maize, as well as medicinal plants.

In short, the thale cress is like the “lab rat” of the plant world—small, easy to care for, and very useful for scientific experiments. Working with this plant has revolutionized our understanding of the genetic, cellular, and molecular biology of plants. For these reasons, it is a favourite among plant scientists.

Background Module S3: How does the thale cress deal with environmental challenges in ecological settings?

Science has shown that the thale cress perceives many different signals in its environment and switches on appropriate responses to deal with environmental factors and adjust its growth behaviour and physiology as needed. Scientists often study the effects of single genes in the perception and responses to the environment with the help of induced mutations, meaning they produce, select and study genetic variants, e.g. with non-functional or high-performance versions of a given gene. Scientists also study the natural genetic diversity associated with a given gene in nature, e.g., by making use of the collection of accessions with sequenced genomes. Already, the genome information of several hundred *A. thaliana* accessions has been determined. By relating phenotypic variation with genomic DNA sequence variation, it is possible to associate phenotypic traits with genomic loci and genetic variation. This helps understanding the importance of genes and their variants for survival of plants in ecological settings. The genetic makeup of an organism is also called genotype (Table S2). In the following section, one example of an environmental response situation illustrates the genotype-phenotype relationship.

Example: When is the best time to flower?

FLOWERING LOCUS C (*FLC*) and FRIGIDA (*FRI*) are two proteins that affect the time to flowering (Johanson et al. 2000). It was found that the *FRI* protein activates the *FLC* gene, and the *FLC* protein suppresses flowering. Genotypes with functional genes (*FLC*, *FRI*) or their mutants can be studied to ask questions like: What is the effect of *FLC*-gene and *FRI*-gene variation on plant growth and reproduction in different environments?

Plants with a functional *FLC* remain for a long time in the vegetative stage to produce green leaves but no flowers. *flc* knockout plants, instead, do not have this suppressing function by *FLC*, and consequently produce few leaves and flower rapidly (Mattioli et al., 2022). Now, it can be asked in which environments, flowering early or late provides an advantage. In the laboratory, it is obviously an advantage if plants flower early. Indeed, scientists are more likely to multiply and study plants that reproduce rapidly. Hence, scientists have selected early-flowering plant variants in their labs, e.g. those that lost the function of *FLC*. In the wild, it is an advantage to flower in favourable growth seasons, but not in cold winters or in dry very hot summers. The *FLC* suppressor gene gets inactivated by a prolonged cold period of several weeks. After the inactivation, plants will then flower rapidly once the cold period is finished. Natural variation studies have shown that in northern Europe, intact *FRI* and *FLC* variants are preponderant in the populations, so that *FLC* protein

suppresses flowering to better tolerate freezing in winter. Flowering then occurs early in spring. In contrast, plants in southern Europe may lack the *FRI* and *FLC* functions because the winter temperatures are mild and it is not harmful but rather beneficial for plants to flower (Caicedo et al., 2004).

This example shows well that evolution can work by selection of genotypes in an ecological setting: There may not be a best general solution, just a best adapted solution for a particular place and time.

The knowledge on the genetic make-up of the thale cress serves to improve crops, such as maize or wheat. *A. thaliana* has greatly facilitated discovery of genes, which are relevant in agronomic contexts as well, such as stress resilience and pathogen resistance. Continuously improving crops is necessary to produce sufficient food in a changing environment.

Text S3: Three examples of how to analyse and discuss statistical outcomes

In the Supplemental File / Text S3/ Background Modules S4-S6, we explore three important but easy-to-understand concepts in statistics, specifically:

1. Understanding and exploring correlations between two (or more) variables.
2. Comparing two groups of data to each other to see if they differ or not.
3. Exploring the question what model explains the data best (as there are infinite models, but not all models are equally good).

We believe that these concepts can be more easily understood if they are explained with actual data, preferably data collected by the school students themselves. The emphasis should always be on how to use the statistical analysis to answer an actual question about the data (e.g., do plants become larger with age?). However, other example data (some given in our examples) can also be used. There are many examples and data in the literature and on the internet which can be used. We suggest that teachers work out their own examples or work together with local scientists.

We emphasise that we collected examples of illustrating texts and videos useful for teaching the basic genetic concepts and techniques used in this school project as well as free statistical resources in Supplemental File / Text S6. Many helpful examples can be found in these resources, and of course many more can easily be found on the internet these days.

Background Module S4: Understanding and exploring correlations ("correlation is not causation").

One of the most basic statistical concepts is the correlation between the data from two variables (Miles and Shevlin, 2000; Shipley, 2000; Baker, 2018a; Baker, 2018b; Thrane, 2024; Ravid, 2025). Most often, we want to know something like: when this one variable increases, does the other variable increase, decrease, fluctuate, or not change at all? Everybody knows examples for this: over the time of the day (time, first variable), temperature (temperature, second variable) increases until noon, then decreases again, but may also fluctuate for various reasons. Or with age (age, first variable), a tree usually becomes taller (size, second variable), but the likelihood of survival decreases (survival rate, another second variable). As in Figure S2, we can plot the two variables and calculate some statistical measure for the strength and direction of the relationship (e.g., a linear regression; Graphpad, 2025). In our example in Figure S2, we get a highly significant result, which tells us that, statistically speaking, the two variables are very much related to each other in that, when age increases, so does height. Thus, we say, these two variables are *statistically* related.

However, a statistical relationship may or may not be a causal relationship. Therefore, the common warning among scientists is: Correlation is *not* causation (Baker, 2018b)! And, of course, what we really want to understand is whether these two variables are *causally* related. In other words, if one variable changes, then does the other variable also change *because* of the change in the first variable, and why?

And we all know examples when this is true and when this is false. For example, one could show in a graph that the more poison a plant is exposed to, the likelier this plant is to die, up to a point where every exposed plant would die (100% mortality). We could then use the data to calculate a *statistical* correlation and would most certainly get a very high, statistically significant P-value. And because we understand how the toxin interrupts the functions of cells, we can conclude that it is indeed the poison which causes the sickness or death. That is then the *causal* relationship between the variable *amount of poison* and the variable *likelihood of death*.

Similarly, we understand the biological mechanisms by which a tree grows every year using nutrients, sunlight and photosynthesis, so we also see a causal relationship between the age of a tree and its height. Likewise, we all know examples of false correlations. For example, if we assume that our example tree began growing in 1960 and was last measured in 2015, we could look up the global gross national product (GNP) and notice a remarkable and *statistically* significant correlation between the growth of the global economy and the height of our tree. However, it would be very hard to come up with any possible *causal* relationship between the growth of the global economy and the growth of this tree (and, indeed, millions of other trees). Because we kind of know how the world

works, we would dismiss the possibility that the growth of the global economy in some way or another *caused* the growth of the tree. Such a relationship which can be seen statistically but is not real is also called a *spurious* relationship.

Now these are very clear-cut and obvious examples of true and false correlations. Science becomes interesting and challenging when we begin to explore correlations and then attempt to proof causal relationships. Such challenges can be illustrated to students in the classroom: Letting students measure several morphological and environmental variables of *A. thaliana* individuals (or of any other available plant species) is a great way of teaching the concept of correlation. Looking at a correlation graph such as Figure S2, for example, could be a great way to start a discussion with the students. Why is there so much variation between different plants? Why are some plants still so small even in week 7? What variables may explain this? The light conditions? The soil nutrients? The genetic makeup? Or were the small plants infested by a pathogen or injured? Some of these variables are difficult to measure, but some can easily be measured even by school students. For example, dividing light exposure conditions into light and shady categories is very simple, and soil pH can be easily measured.

The one or two variables of interest identified through discussion with the students can then be experimentally tested in a plant growth experiment, e.g., on the window of the classroom (for analysis, see Supplemental File / Text S3/ Background Module 5).

Background Module S5: Comparing two groups.

The second one of the most basic statistical concepts is the comparison between two groups (which can then be extended to several groups) (Watt, 1993; Watson and Moritz, 1998; McDonald, 2008; Ruxton and Stafford, 2015; Ravid, 2025). Oftentimes, binary variables are to be compared. Binary means that an individual can be in one of two states, e.g., either it belongs to species A or species B. For example, are there size differences between individuals of two different species? Do the vitamin C contents vary between two types of fruits? Do plants with green leaves grow faster than plants with red leaves?

As suggested in Supplemental File / Text S3/ Background Module S4, a very simple growth experiment can test the influence of a binary variable; e.g., is the rosette of thale cress plants larger or are plants taller in light or shady environments (testing the effect of the binary variable light versus shade)?

There are several kinds of statistical tests for two-group comparisons (see Supplemental File / Text S6), one of which is the Mann-Whitney U test (Siegel, 1956; Siegel and Castellan, 1988). These are simple to calculate with free internet tools (e.g., Social Science Statistics, 2025b). The Mann-Whitney U test can detect a statistically significant difference in the measured data (e.g., height, weight, number of flowers, or any continuous variable) of light-exposed plants versus shade-exposed plants (Table M5-1). The data generated from such a simple experiment can then serve as a starting point for introducing statistical analyses and helping students understand how scientific results are interpreted. For example:

1. First, we analyze made-up data presented in Table M5-1. The mean height of the plants grown in light conditions is 21.8 cm, while the mean height of the plants grown in shady conditions is 20.2 cm, and this difference is statistically significant. However, if just one data point is changed by 3 cm, this statistical difference can either disappear or become much larger, even though the means are only slightly different. From this simple example, the following statistical topics can be discussed: First, when sample sizes are small, statistically significant differences are very sensitive to even slight changes in data measurements. Therefore, our confidence in these results is not particularly high, even if we have a statistically significant difference at the $P < 0.05$ level. Second, the only way to increase confidence that this *statistical* difference also represents a *real-world* difference is to increase the sample size. Lesson number one: the larger the sample size, the greater our confidence in the statistical result.

2. In Supplemental File / Text S3/ Background Module S4, we learned that a statistical relationship could mean that there is a causal relationship – or not. Likewise, a *statistical* difference

can mean that the binary variable is the *cause* of this difference – or not. Since we know from many different lines of evidence that light is essential for plant growth, we could be rather confident that the difference in light conditions *causes* the difference in growth. However, we could also be looking at a *spurious* difference if, for example, another variable was also different for the two treatments. Maybe the shady light conditions were also cooler or more humid than the sunny ones, and perhaps not light but temperature or humidity were the relevant effects. Lesson number two: the better we control and keep constant the other possibly important variables (such as temperature, humidity, etc.), the greater our confidence in the result.

3. Even if we can argue that the *statistical* difference is also a *causal* difference because (1) we controlled for all the other possibly important variables, and (2) our sample size was large, we can still discuss if the observed difference represents an *important* difference in the real world. After all, the difference in mean height is only 1.6 cm in our example! If the goal is to increase yield in food production, and a treatment results in a statistically significant increase, it is still worth asking whether the use of the treatment is actually justified. The treatment may involve something as simple as light versus shade, but it could also include daily watering versus weekly watering, or even the use of an expensive chemical fertilizer versus a cheaper biological alternative. We might then actually find a *statistically* significant difference, and because we know that light, water and nutrient uptake are important for plant growth, we may also believe that the treatment is the *causal* factor for significantly better growth. However, if the increased water treatment or the chemical fertilizer treatment increases the overall growth of plants by only a few percentage points compared to the plants growing in the control conditions, then the treatment might work but may nevertheless not be worth it. Therefore, lesson number three is: even great statistical confidence in the result is not equal to significant implications in the real world. Hence, the results and applications need to be discussed in their ecological, social, economic or environmental context!

Our point is that even a very simple experiment with a very simple plant and a relatively small sample size can be used as a starting point to have many interesting lessons and discussions about how science works, how we need to design reliable experiments, and what type of valid conclusions are meaningful.

Table M5-1. Hypothetical example of the height of *A. thaliana* plants (in cm) having grown in light and shady conditions (meaning the data presented here were made up for illustrative purposes). The height should be measured once the plants have fully grown. Here, the mean height of the plants grown in light conditions is 21.8 cm, and the mean height of the plants grown in shady conditions is 20.2 cm. One typical statistical test for these kinds of data is the Mann-Whitney U test, which would yield a statistically significant difference between the two treatments groups (n = 10 for each treatment, U = 22.0, P = 0.03), but with a P-value only a little bit below the significance level of $P < 0.05$. Because of the small sample size, the statistical difference could disappear if, for example, only one data point* was changed from 20 to 23 (U = 28.5, P = 0.10). Or the statistical difference would increase considerably if, for example, another data point$ was changed from 20 to 23 (U = 16.5, P = 0.01).

	Individual plants and their measurements (in cm)									
Treatment	1	2	3	4	5	6	7	8	9	10
light	22	23	21	20$	20	21	21	22	24	24
shady	22	18	19	20	21	20	19	22	21	20*

Background Module S6: What model explains my data best?

The third one of the most basic statistical concepts is the making of a model to explain or predict how something works (Miles and Shevlin, 2000; Burnham and Anderson, 2002; Johnson and Omland, 2004). How we build models and how we interpret them goes to the core of how we do and use science: “models are a cornerstone of every science discipline” (see Figure 1 in Passmore et al., 2009). Oftentimes, model predictions and resulting actions are controversially discussed, e.g., the models predicting future climate and climate change events such as floods, or model predictions on the spread of the coronavirus with or without measures such as lockdowns, mask-wearing and vaccinations. Much of those controversies revolve around the building, use, and interpretation of scientific models.

There is a large amount of literature about model building (e.g., Guy-Gaytán et al., 2019, Supplemental File / Text S6). Here, we give a very simple example about model building using the study plant *A. thaliana*. To illustrate how models are used to interpret data, we used four different models to fit our data (Figure S2).

First, we can simply draw a straight line through our data, and, in our example, this line will indicate *on average* what the length of a plant will be given it has grown for a given (or x) number of weeks (Figure S2A). The mathematical form of the model is then

length (y) = a + b * x * weeks

where x is the number of weeks, b is a constant number which determines how steep the slope of the line is (in this case, the length increase per time unit), and a is another constant number which determines where the line crosses the horizontal x-axis (see also Figure 4 in Bryce et al., 2016). This type of model is called the linear model and has been used in thousands of studies in just about every scientific discipline. That is because it is easy to calculate and understand. And we do have many examples of such linear relationships: e.g., if you collect fresh biomass, dry it sufficiently and then measure the resulting dry biomass, you get an almost perfect linear relationship if you measure many different samples.

However, how do we place the line in the right place? An infinite number of different lines could be placed across the data. Each one would represent another attempt at a model, but clearly some linear models would be better than others (e.g., any decreasing line would be a bad model for this example). This is where model building becomes trickier and more mathematical. Using certain assumptions, a good fitting line is found with mathematical algorithms which calculate the best fitting linear regression, and the resulting line is shown in Figure S2A.

However, even though this line is a good model of the data, it is and can never be the *true* model of the data, but only a (more or less) good explanation of the data. For example, the linear model in Figure S2A suggests that plants would keep growing at a constant rate indefinitely, eventually reaching unrealistic sizes. While the linear model may therefore be okay for the first eight weeks of growth, it is obviously not a true model for the entire life cycle of the plant.

Second, we can draw a curve through our data, as in Figure S2B. The so-called polynomial model could be a better explanation for the pattern in our data, as plants will stop growing in height at some point. Polynomial models allow for various 'curvy' lines to be drawn through the data. For example, a polynomial model explains the growth of the leaf area over time quite well (y = a+bt+ct2+dt3, where t is time and a, b, c and d are constants). Therefore, polynomial models have also been used in thousands of studies. Again, we could draw an infinite number of polynomial curves through the data, and again, an algorithm helps to determine a 'best' curve to fit the data.

Third, we could use an exponential model to draw a curve through our data (Figure S2C). Exponential models have also been used in thousands of studies: typical examples include the exponential increase in nuclear reactions in nuclear bombs, or the initial, early growth of seedling biomass in *A. thaliana*. Even though this model also achieves high statistical significance, the resulting prediction of exponential growth is obviously wrong, as the rapid initial growth must slow down and stop eventually in every living being. So here is another lesson: statistical significance does not equate to biological significance! We always need to check whether our statistical results make sense in the real world.

Actually, the three models presented in Figures S1A-C are all somewhat faulty, because we already know through much previous research that the best model to use for plant growth is actually the so-called S-model (also called sigmoidal model; Figure S2D; see also a very nice illustration in Figure 1 in Tomiolo et al., 2022 and Figures in Knopf and Bauer, 2025). This model means that, in the beginning, growth is initially slow (during establishment), then becomes rapid (increases exponentially) but then slows down asymptotically when plants become mature. All living beings and all living systems must abide by the asymptotic growth model. So here is a fun question for a lot of follow-up discussion: "There is a reason why, in nature, things grow in size only to a certain point. So why do most economists and politicians think that the economy can grow forever and ever and ever?" (Undercurrents Media, 2010).

Since this is the growth pattern which we observe in most plants, the S-model is most likely the most appropriate for our data, too. This is statistically corroborated by the fact that the S-model has the lowest AICc (yes, in this case, lowest is best!), a statistical measure which evaluates the fit of the model to the data.

The main points here are as follows.

1. In science, models are being built and revised constantly to explain patterns and processes (for a framework for use in schools, see Passmore et al., 2009). Some models explain the patterns and data better. In a video about climate models, Schmidt (2014) said: “Models are not right or wrong. They are always wrong, they’re always approximations. The question we have to ask is whether a model tells you more information than you would have had otherwise [without the model].” Therefore, even a very good model can be improved upon – scientific inquiry never ends in some absolute truth. However, this does not mean that we can be very certain about some things, e.g., gravity, electromagnetism, or the role of DNA. In science, we basically have different levels of certainty: some things are very certain and will not be debated (e.g., that the earth is round, unless some extraordinary new evidence turns up), others are rather certain but may still be debated, some things are uncertain and are therefore hotly debated, and some ideas are almost certainly wrong (e.g., the flat earth being an oft-cited example) and are only debated by conspiracy theorists.

2. In the end, models *always* need to be tested against data, and best against new data. Therefore, scientists constantly take more data and think of better, more accurate ways to get new data. If the model agrees with the new data, great. If not, probably a better model needs to be built or an existing model needs to be revised (see point 1 above). This insight can be used to encourage students to repeatedly collect data, every time with the intention of testing their model and then, if needed, to improve it.

3. Another important point to remember is that *different* models can and should be used for different data, *different* questions, and different uses (see, e.g., Figures 1-2 in Bryce et al., 2016). To quote Svoboda and Passmore (2013): “The implication is that there is not a single type of model or modelling that can address all biological problems equally well; depending on the question at hand, a biologist will want to choose the model that is the best tool for the job.” A model for plant cell development is obviously different to a model for plant population dynamics. Sometimes simple models are the best (e.g., the relationship between gravity and acceleration), and sometimes complex models are the best (e.g., modelling the climate of the entire Earth). Teachers can emphasize the diversity of ways in which modelling supports scientific inquiry so that, together with their class, they can make decisions about how exactly to incorporate models into their teaching.

What we want to show here is that a teacher can begin deep discussion about how science works even by looking at some simple morphological growth data of a plant, which is easily accessible for measurements by students. Then, the discussion can continue to emphasize the role of models and to teach the students to ask questions, recognize data patterns, construct explanations for data, and provide criteria for judging knowledge claims (Passmore et al., 2009).

Text S4. German text for the school project about laboratory techniques and analyses for genotyping by student classes in secondary schools. Copyright for all photos by Bruno Andreas Walther, unless another name or link is given. An English translation of this text is given below as Supplemental File / Text S5.

PUKI-Projekt für die Sekundarstufe

Vorwort

Der Zweck dieser Anleitung besteht darin, die Zusammenarbeit zwischen Lehrkräften, Schülern und Wissenschaftlern durch ein genetisches Ökologieprojekt zu fördern. Da viele Schulen möglicherweise nicht über die notwendige Ausrüstung und Materialien verfügen, ist es entscheidend, dass Wissenschaftler entweder die Schule besuchen oder die Schule die Wissenschaftler aufsucht, um bei der Durchführung der Schritte zu assistieren. In diesem kollaborativen Umfeld können Lehrer und Schüler gemeinsam mit Wissenschaftlern erörtern, ob und welche Schritte im Klassenzimmer oder Schullabor demonstriert werden können.

Die Anleitung ist bewusst flexibel gestaltet, sodass jeder Schritt bis zur vollständigen Sequenzierung im Klassenzimmer übersprungen und den Wissenschaftlern überlassen werden kann. Beispielsweise könnte die Präparation der genomischen DNA einfach in der Schule erfolgen, während die restlichen Schritte im Labor durchgeführt werden. Die Endergebnisse der Sequenzierung könnten dann im Unterricht präsentiert und erklärt werden.

Unser Ziel ist es, einen anpassbaren Vorschlag zu unterbreiten, der auf die spezifischen Bedingungen und Bedürfnisse vor Ort abgestimmt werden kann. Ähnlich wie in einem professionellen Labor, in dem viele Aufgaben auf verschiedene Weise erledigt werden können, sollte auch die Umsetzung dieser Anleitung flexibel gehandhabt werden. Wir hoffen, dass diese Anleitung dazu inspiriert, moderne genetische Methoden praktisch im Unterricht zu demonstrieren. Alternativ ist es auch möglich, die Ergebnisse unserer Studie zu verwenden, um sie im Unterricht zu analysieren, vergleichen und zu besprechen.

Das Flussschema zur DNA-Genotypisierung (Abbildung 3A) bietet einen Überblick über die durchzuführenden experimentellen Schritte und die Analyse der genetischen Daten des *FRI*-Locus von wild wachsenden *A. thaliana* Pflanzen mittels PCR-Sequenzierung. Grundlegende Labortechniken sind aufgelistet, und wir verweisen auf informative Lehrvideos, Lehrbücher und Bildungswebseiten als weiterführende Ressourcen (Supplemental File / Text S6). Da die Arbeitsschritte von den vorhandenen Geräten und den örtlichen Gegebenheiten abhängen, verzichten wir auf eine allgemeingültige Anweisung. Dennoch ist es möglich, gängige Labormethoden mit fachlichem Wissen problemlos auf die Schulsituation zu übertragen. Falls dies in der Schule nicht im Alleingang möglich ist, sollten die experimentellen Schritte in Zusammenarbeit mit Forschungseinrichtungen und mobilen Schülerlaboren durchgeführt werden. Unabdingbar ist dabei, dass die Schüler die Laborsicherheitsvorschriften im Umgang mit Geräten und Chemikalien verstanden haben.

Allgemeine Labortechniken

Kenntnisse über verschiedene Basistechniken des molekularbiologischen Labors sind nötig, die hier nicht beschrieben werden. Vielmehr ist eine erfahrene Lehrkraft nötig, welche die Schüler anleitet und Laborsicherheit gewährleistet. Diese Basistechniken sind im Folgenden aufgelistet:

- Herstellen von Lösungen und Verdünnen, Volumen und Konzentration.
- Utensilien im molekularbiologischen Labor. Abbildung 3. Akkurates Pipettieren mit der
- Umgang mit der Mikropipette.
- Mischen (Vortex) und Zentrifugieren.

Spezifische Labortechniken

Hintergrund: Genotypisierung des *FRIGIDA* (*FRI*) Gens

Durch Genotypisierung wird im Labor überprüft, welcher genetische Bauplan für ein bestimmtes Gen (oder mehrere Gene) in einem Lebewesen vorliegt. Jedes Gen existiert in etwas verschiedenen Versionen (also verschiedene DNA-Buchstabenabfolgen oder Sequenzen), und diese Versionen oder Varianten heißen Allele. Als ein Beispiel für die Bestimmung von Allelen eines Gens wollen wir hier einen aussagekräftigen Ausschnitt der genomischen DNA von dem sogenannten *FRIGIDA* (*FRI*) Gen untersuchen. Dazu werden zuerst Blattproben von *A. thaliana* Pflanzen gesammelt (Schritt 1).

Um den Genotyp der Pflanzen gemäß des Flussschemas zur DNA-Genotypisierung zu ermitteln, muss zunächst die genetische Information, also die DNA, aus den Pflanzen gewonnen werden. Dieser Schritt heißt DNA-Extraktion (Schritt 2). Dabei wird die Pflanzenprobe zerkleinert und aufgebrochen, damit die Zellwände und Zellmembranen zerfallen. Die freigesetzte genomische DNA kann gereinigt und gefällt werden. Schließlich wird die DNA in einer gepufferten Lösung gelöst und gelagert. Nachdem die DNA in Lösung ist, wird in einem weiteren Schritt ein bestimmter Abschnitt des Gens vervielfältigt. Das geschieht mit einer Methode namens PCR (Polymerase-Kettenreaktion) (Schritt 3). Diese Technik sorgt dafür, dass genau ein bestimmter DNA-Abschnitt des *FRI*-Gens millionenfach kopiert wird. Die Vervielfältigung dieses PCR-Fragments ist wichtig, um ausreichend Material für die weitere DNA-Analyse zu haben.

Das Ergebnis der PCR muss noch überprüft und dafür sichtbar gemacht werden. Hierzu wird die DNA nach der PCR in einer Gelelektrophorese aufgetrennt (Schritt 4). Da die DNA negativ geladen ist, werden DNA-Fragmente in einer Gelmatrix in einem elektrischen Feld gemäß ihrer Größe aufgetrennt. Kleine DNA-Teile bewegen sich schneller durch das Gel, große langsamer. Das Gel enthält Farbstoffe, die leuchten, wenn sie sich an die DNA binden. So sieht man unter UV-Licht die sogenannten Banden, die die

jeweiligen DNA-Fragmente repräsentieren. Anhand der Größe und Anzahl der Banden kann man bereits Rückschlüsse auf das Vorhandensein des gewünschten Gens und eventuelle Varianten ziehen.

Weitere und viel detailliertere Informationen über die Sequenzen erhält man durch das Sequenzieren der PCR-Produkte. Hierfür werden die PCR DNA-Fragmente aus dem Gel ausgeschnitten und aufgereinigt (Schritt 5). Anschließend werden die Sequenzen, d.h. die Basenabfolgen A, C, G, T (d.h., die vier Buchstaben der DNA-Sequenzen) der aufgereinigten DNA-Fragmente mithilfe der Sanger-Sequenzierung bestimmt (Schritt 6). Die erhaltenen DNA-Sequenzen werden untersucht und schließlich die *FRI*-Sequenzen der verschiedenen gesammelten Pflanzenproben untereinander und mit der genomischen DNA-Sequenz der Referenzlinie Col-0 verglichen (Schritt 7).

Das Flussschema (Abbildung 3A des Haupttextes) stellt die einzelnen Arbeitsschritte grafisch dar.

Schritt 1: Sammeln der Blattproben

Materialien:

- 1,5 ml Plastik Reaktionsgefäße
- Pinzette, Messer

Blattmaterial (in der Größe etwa einer 1-Cent Münze) wird in ein 1,5 ml Plastik Reaktionsgefäß überführt. Pro Pflanze wird ein Gefäß benötigt. Die Gefäße müssen ordentlich beschriftet werden, z.B. mit der Pflanzennummer aus der Liste mit Pflanzenstandorten und Individuen. Das Blattmaterial sollte möglichst gesund und grün sein. Alternativ können auch andere Pflanzenteile verwendet werden. Die Pflanzenproben können im Kühl- oder Gefrierschrank gelagert werden.

Schritt 2: Extraktion der genomischen DNA

Materialien:

- 10 ml DNA Exktraktionspuffer
- Plastikhandmörser
- Reinstwasser, steril
- Mikropipetten und Pipettenspitzen (200 µl, 1 ml)
- Ständer für Reaktionsgefäße
- Mikrozentrifuge für 1,5 ml Gefäße

Um die gDNA der Pflanzenproben zu erhalten, werden die Pflanzenteile zunächst zerkleinert und die Zellen der zerkleinerten Pflanzenteile aufgelöst, so dass die DNA aus ihnen heraustreten kann (Abbildung 6). 150 µl Exktraktionspuffer werden dazu in die Reaktionsgefäße mit Blattproben pipettiert; der „Magic“-

Puffer enthält Saccharose, Salz und TRIS (Tris-(hydroxymethyl)-aminomethanhydrochlorid). Mit einem Plastikhandmörser wird die Pflanzenprobe zerrieben, bis die Lösung grünlich und trüb ist. Zur Probe wird nun zusätzlich 1350 µl Reinstwasser hinzugegeben und vorsichtig gemischt. Die Proben werden zentrifugiert (1 Minute bei 5000 RPM), bis sich die Pflanzenreste unten als ein Pellet abgesetzt haben. Aus dem Überstand wird die Probe für die PCR-Reaktion entnommen.

Die Probe kann bei -20°C gefroren gelagert werden - bei erneuter Verwendung auftauen lassen und erneut zentrifugieren.

Schritt 3: Vervielfältigung von *FRI*-Genfragmenten durch Polymerase-Kettenreaktion (PCR)

Materialien:

- 2X PCR-Mix, z.B. RedTaq Merck
- Primer, z.B. 100 µM
- Reinstwasser, steril
- PCR-Reaktionsgefäße
- ggf. 1,5 ml Reaktionsgefäß
- Mikropipetten und Pipettenspitzen für PCR (200 µl, 20 µl)
- Ständer für Reaktionsgefäße
- Eisbehälter und Eis zum Kühlen
- PCR-Maschine
- Vortex (optional)
- Zentrifuge für PCR-Reaktionsgefäße (optional)

Bestimmte Abschnitte des *FRI*-Gens sollen nun mittels Polymerase-Kettenreaktion (PCR) milliardenfach vervielfältigt werden. Das Prinzip der PCR ist in Biologie Lehrbüchern ausführlich beschrieben, und es gibt auch zahlreiche Lehrvideos (Supplemental File / Text S6). PCR-Reaktionsgefäße werden vorbereitet und beschriftet. Ein PCR-Reaktionsgemisch wird eingefüllt, welches Taq-Polymerase bzw. hitzestabile DNA-Polymerase, Puffer und Salze, Nukleotide (ATP, CTP, GTP, TTP), zwei Primer (F/forward/5‘ und R/reverse/3‘) und Wasser beinhaltet. Genaue Informationen zu Konzentrationen, etc. im PCR-Mix werden in der Herstelleranleitung gegeben. Sollen drei oder mehr PCR-Reaktionen derselben Art angesetzt werden, bietet es sich an, einen Mehrfachmix, den sogenannten **Master-Mix (MMX) in einem 1.5 ml Reaktionsgefäß vorab vorzubereiten**. Diese Mischung enthält alle Bestandteile für die PCR außer gDNA. Der Mastermix sollte gut vermischt und möglichst kurz abzentrifugiert werden. Dann werden 24 µl Mastermix in PCR-Reaktionsgefäße gegeben. Am Ende wird je 1 µl gDNA zugesetzt. Nach Vermischen (optional durch Vortex) und anschließende Zentrifugation (optional) werden die Reaktionsgefäße in das PCR-Gerät gesetzt.

Achtung: Die Pipettenspitzen sollten nach jeder Verwendung entsorgt werden, um Kreuzkontaminationen mit gDNA zu vermeiden!

**Tabelle**: Ansetzen der PCR-Reaktionen inkl. zweier Beispiel-Mastermixe			
Komponente	für die Anzahl der Proben (n)		
	1 (n = 1 Probe)	n + 3 $ (n = 17 Proben)	n + 5 $ (n = 45 Proben)
2X PCR-Mix*	12.5 µl	250 µl	625 µl
Primer 1**	2.5 µl	50 µl	125 µl
Primer 2**	2.5 µl	50 µl	125 µl
Reinstwasser	5.5 µl	110 µl	275 µl
gDNA***	2 µl	2 µl	2 µl
Gesamtvolumen pro Reaktion 25 µl			

* enthält Puffer, Salze, Nukleotide, Enzym

** 10 µM

*** eine Wasserkontrolle ohne gDNA, sondern mit zusätzlich 1 µl Reinstwasser ist ebenfalls anzusetzen

$ Beispielansätze für Mastermixe; die erste Zahl gibt die Anzahl der reellen Proben an; die zweite die Anzahl der zusätzlich kalkulierten Proben, um nach dem Verteilen ausreichend Flüssigkeit für alle reellen Proben zu haben; 24 µl des Mastermixes werden pro PCR Reaktion pipettiert

Eingesetzte Primer-Kombination:

PCR *FRI*

G/E site_F 5‘ CCATAGACGAATTAGCTGC 3‘

16bp-Difference_R 5‘ AGTACTCACAAGTCACAAC 3‘

PCR product = 903/919 bp (903 bp with the *A. thaliana* Col-0 reference accession)

Schritt 4: Auftrennung der PCR DNA-Fragmente durch Gelelektrophorese

Materialien:

- Nitrilhandschuhe und Laborkittel
- TBE Elektrophoresepuffer, ca. 2 l
- MIDORI Green TBE Tablette

- DANN-Größenmarker
- Agarosegelapparatur mit Gussform und Kamm
- Glaserlenmeyerkolben
- Messzylinder für 25 ml
- Mikropipette (200 µl) und Pipettenspitzen
- Mikrowelle
- Fotokamera, Handy für Fotos

Die Gelelektrophorese ist eine analytische Methode, um elektrisch geladene DNA-Fragmente voneinander zu trennen. In diesem Fall werden die PCR-Fragmente von den Primern getrennt. Durch die Midori DNA Färbemethode werden die DNA-Fragmente sichtbar gemacht. Mittels eines Größenstandards wird die erfolgreiche Vervielfältigung der PCR-Fragments festgestellt und die Größe der PCR DNA-Fragments überprüft.

Während der Gelelektrophorese sollten immer **Nitrilhandschuhe und Laborkittel** getragen werden!

25 ml destilliertes Wasser (auf Raumtemperatur) werden mit einer MIDORI Green TBE Tablette in einem Glaskolben versetzt. Diese Tablette enthält den für die Reaktion benötigten Puffer und die nötige Agarose-Konzentration für ein 1-prozentiges Agarose-Gel. Innerhalb von 3 bis 5 Minuten sollte sich die Tablette auflösen. Die Lösung wird dann erhitzt, zum Beispiel mittels einer Mikrowelle (**welche sonst auf keinen Fall für die Zubereitung von Lebensmitteln verwendet werden darf**). Es muss darauf geachtet werden, dass das Gel gelegentlich vorsichtig umgeschüttelt wird und bei der Erhitzung nicht überkocht. Nachdem in der Lösung keine Partikel mehr zu sehen sind, sollte sie 3-4 Minuten abkühlen und dann in eine Elektrophoresegussform mit Kämmen der entsprechenden Größe gefüllt werden. Bis zur vollständigen Erstarrung des Gels vergehen um die 20-30 Minuten. Nachdem dem Erkalten werden die Kämme entfernt und das Gel wird mitsamt der Gussform in die Elektrophoresekammer gelegt. Die Elektrophoresekammer wird daraufhin mit einem Elektrophoresepuffer (dem sogenannten TBE-Puffer) gefüllt, bis das Gel durch ungefähr 0,5 cm TBE-Puffer bedeckt ist und die Gel-Taschen im Gel komplett mit Puffer gefüllt sind.

Nun werden 25 µl PCR-Produkt in die Gel-Taschen pipettiert (für jede Probe eine **neue Plastikspitze** benutzen!). Hier sollte darauf geachtet werden, dass

1. eine Gel-Tasche freibleibt (hier wird später der DNA-Größenmarker hinzugefügt);
2. die Proben sehr langsam und vorsichtig pipettiert werden, da die Gel-Taschen sonst überlaufen, was zu Fehlergebnissen führen würde;
3. nicht zu langsam gearbeitet wird, da die Proben mit der Zeit im Gel verlaufen, auch ohne, dass die elektrische Spannung der Elektrophoresekammer eingeschaltet wird. Das würde dazu führen, dass die Endergebnisse uneindeutig werden. Sehr wichtig ist natürlich, sich vorher **schriftlich einen Plan zu machen**, welche Gel-Tasche mit welcher Probe gefüllt werden wird.

Zuletzt werden dann 3 µl des DNA-Größenmarkers (namens 1 kb plus) in die noch leere Tasche gegeben. Der DNA-Größenmarker ist ein Gemisch von DNA-Strängen von bekannter aber unterschiedlicher Länge. Er dient sozusagen als Kontrolle, denn wenn die DNA-Größenmarker am Ende der Gelelektrophorese nicht am richtigen Ort angekommen sind, ist etwas falsch gelaufen.

Nachdem DNA-Leiter und alle Proben in die Gel-Taschen gefüllt wurden, wird das Gel in die Elektrophoresekammer gesetzt, dann wird der Deckel der Elektrophoresekammer aufgesetzt, und die Kammer wird eingeschaltet und laufen gelassen, bis eine klare Aufspaltung des Farbmarkers durch den Deckel ersichtlich ist. Dies sollte ca. 35 Minuten beanspruchen. Um die Ergebnisse festzuhalten, kann ein Foto mit einer beliebigen Kamera gemacht werden; für optimale Bilder sollte ein schwarzer, faltbarer Aufsatz auf die Kammer platziert werden, um das Foto durch ein oberes Loch aufzunehmen.

Eine eindeutige DNA-Einzelbande der ca. erwarteten Größe sollte bei einer erfolgreichen PCR-Reaktion erkennbar sein.

PCR-Programm, welches in die Elektrophoresekammer eingegeben wird:

1. Initiale Denaturierung: 2 Minuten, 96°C
2. 35 Zyklen der folgenden Schritte:
- Denaturierung: 30 Sekunden, 96°C
- Annealing der Primer: 35 Sekunden, 55°C
- Extension: 45 Sekunden, 72°C
3. Finale Extension: 60 Sekunden, 72°C

Schritt 5: Aufreinigung der PCR-Fragmente

Materialien:
- Gelaufreinigungs-Kit
- 1,5 ml Reaktionsgefäße
- Mikropipette (200 µl) und Pipettenspitzen
- Feinwaage (optional)
- Zentrifuge für 1,5 ml Reaktionsgefäße
- Wärmebad

DNA-Banden werden direkt aus dem Gel geschnitten, wobei man ein passendes Schneidewerkzeug benutzt (z. B. ein Skalpel). Es sollten nur die klar sichtbaren Banden ausgeschnitten werden, und Schleier über oder unter den leuchtenden Banden sollten ausgenommen werden. Die möglichst eng ausgeschnittenen Gelstücke werden jeweils in **leere und beschriftete 1,5 ml Reaktionsgefäße** platziert. Die **Masse des**

ausgeschnittenen Gelstückes kann nach Abzug des Leergewichtes des Reaktionsgefäßes bestimmt (optional) oder abgeschätzt werden. Dann erfolgt die Gelaufreinigung gemäß der Angaben des Kit-Herstellers. In der Regel wird das Gelstück mit einem Puffer aufgelöst, dann die DNA-Fragmente in einer Säule gebunden, gewaschen und schließlich mit 10 µl Elutionspuffer eluiert. Dies resultiert in einer finalen Konzentration von ca. 3 ng PCR DNA-Fragmente/µl.

Schritt 6: DNA-Sequenzierung nach Sanger

Materialien:

- 1,5 ml Reaktionsgefäße
- Mikropipette (200 µl) und Pipettenspitzen
- Primer
- Reinstwasser
- Labels oder Strichcodes (von einer DNA Sequenzierfirma für Sanger DNA-Sequenzierung für Einzelproben anzufordern)

Bei der DNA-Sequenzierung nach Sanger wird die Basenabfolge auf einem DNA-Strang mit Hilfe eines sequenzspezifischen Primers für den DNA-Strang ermittelt. Diese Reaktion wird heutzutage von Firmen als Dienstleistung durchgeführt. Das Prinzip der Sanger DNA-Sequenzierung ist in Lehrbüchern, Lehrwebseiten und Lehrvideos ausführlich beschrieben (Supplemental File / Text S6). Für jedes PCR DNA-Fragment werden zwei Sequenzierreaktionen angesetzt, um die Sequenzen von beiden Enden des Fragmentes aus einem mit dem F und einmal dem R Primer zu ermitteln. Dafür werden zwei Reaktionsgefäße vorbereitet und markiert. Gemäß der Angaben der Sequenzierfirma werden z.B. 10 pmol F bzw. R Primer (10 µM Ausgangslösung), bis zu 200 ng PCR-Produkt und Reinstwasser in einem bestimmten von der Sequenzierfirma vorgegebenen Volumen versetzt. Die Reaktionsgefäße werden z.B. mit Strichcodes oder Labels der Sequenzierfirma beschriftet und eingeschickt zur Sanger-Sequenzierung. Später werden die Ergebnisse unter Angabe der entsprechenden Strichcode Nummern elektronisch von der Firma zur Verfügung gestellt.

Schritt 7: Sequenzanalyse der *FRI*-spezifischen DNA-Fragmente:

Nach Erhalt der Sequenzierungsdaten werden die Vorwärts- und Rückwärtssequenzen untersucht und daraus jeweils eine qualitativ nützliche DNA-Sequenz pro Probe zur weiteren Analyse im FASTA-Format erzeugt. Sequenzbereiche niedriger Qualität, etwa an den Enden der Sequenzläufe, werden entfernt. Die Bearbeitung und Zusammenführung der Sequenzdaten kann mithilfe der frei verfügbaren Plattform Benchling erfolgen

(Benchling [Biology Software]. (2025). Retrieved from https://benchling.com.), welche die Visualisierung, Editierung und den Vergleich von DNA-Sequenzen ermöglicht.

Für die vergleichende Analyse wird die Referenzsequenz des *FRIGIDA* (*FRI*)-Gens der *A. thaliana* Referenzlinie Col-0 von The Arabidopsis Information Resource (TAIR, www.arabidopsis.org, AT4G00650) bezogen. Die erstellten Konsensus-Sequenzen der einzelnen Pflanzenproben werden anschließend mithilfe eins Alignment Viewer and Editor Programms, etwa AliView (Larsson et al., 2014), gegen die Col-0-Referenzsequenz ausgerichtet. Das Multiple-Sequence-Alignment dient der Identifikation von Sequenzunterschieden zwischen den untersuchten Proben, die auf Polymorphismen hindeuten können.

Zur Unterstützung der Interpretation und Darstellung der Ergebnisse wird auf Basis der Alignments eine vereinfachte grafische Darstellung der Allelunterschiede erstellt. Dieses Vorgehen ermöglicht eine übersichtliche Visualisierung genetischer Variation und eignet sich insbesondere für den Einsatz im schulischen Kontext.

Text S5. English text for the school project about laboratory techniques and analyses for genotyping by student classes in secondary schools. Copyright for all photos by Bruno Andreas Walther, unless another name or link is given. A German translation of this text is given below as Supplemental File / Text S4.

PUKI project

for secondary school students

Preface

The purpose of this guide is to promote collaboration between teachers, students, and scientists through a genetic ecology project. As many schools may not have access to the necessary equipment and materials, it is essential that scientists either visit the school or that the school visits the scientists in order to assist with the implementation of the individual steps. Within this collaborative setting, teachers and students can discuss together with scientists whether and which steps can be demonstrated in the classroom or school laboratory.

The guide is deliberately designed to be flexible, so that any step up to complete sequencing can be omitted in the classroom and instead carried out by scientists. For example, the preparation of genomic DNA could be performed at school, while the remaining steps are carried out in the laboratory. The final sequencing results could then be presented and explained during class.

Our aim is to propose an adaptable framework that can be tailored to specific local conditions and needs. Similar to a professional laboratory, where many tasks can be performed in different ways, the implementation of this guide should also be handled flexibly. We hope that this guide inspires the practical demonstration of modern genetic methods in classroom teaching. Alternatively, it is also possible to use the results of our study for analysis, comparison, and discussion in class.

The workflow for DNA genotyping (Figure 3A) provides an overview of the experimental steps to be carried out and the analysis of genetic data of the FRI locus from wild-growing Arabidopsis thaliana plants using PCR sequencing. Basic laboratory techniques are listed, and we refer to informative instructional videos, textbooks, and educational websites as additional resources (Supplemental File / Text S6). As the individual steps depend on the available equipment and local conditions, we refrain from providing a universally applicable protocol. Nevertheless, common laboratory methods can be readily transferred to the school context with appropriate expertise. If this cannot be accomplished independently at school, the experimental steps should be carried out in cooperation with research institutions and mobile student laboratories. It is essential that students have understood laboratory safety regulations when handling equipment and chemicals.

General Laboratory Techniques

Knowledge of various basic techniques used in a molecular biology laboratory is required and is not described in detail here. Instead, an experienced teacher is necessary to guide the students and ensure laboratory safety. These basic techniques are listed below:

- Preparation of solutions and dilutions; understanding volume and concentration.
- Equipment used in the molecular biology laboratory (Figure 3: accurate pipetting).
- Handling of the micropipette.
- Mixing (vortexing) and centrifugation

Specific Laboratory Techniques

Background: Genotyping of the *FRIGIDA (FRI)* Gene

Genotyping is used in the laboratory to determine which genetic blueprint for a specific gene (or multiple genes) is present in an organism. Each gene exists in slightly different versions (i.e., different DNA base sequences), and these versions or variants are referred to as alleles. As an example of determining alleles of a gene, we examine here a representative fragment of the genomic DNA of the so-called *FRIGIDA* (*FRI*) gene. For this purpose, leaf samples from *Arabidopsis thaliana* plants are first collected (Step 1).

To determine the genotype of the plants according to the workflow for DNA genotyping, the genetic information, namely DNA, must first be extracted from the plants. This step is referred to as DNA extraction (Step 2). During this process, the plant sample is ground and disrupted so that cell walls and cell membranes break down. The released genomic DNA can then be purified and precipitated. Finally, the DNA is dissolved in a buffered solution and stored. Once the DNA is in solution, a specific region of the gene is amplified in a subsequent step. This is achieved using a method called PCR (polymerase chain reaction) (Step 3). This technique ensures that a defined DNA fragment of the *FRI*-gene is copied millions of times. Amplification of this PCR fragment is important in order to obtain sufficient material for further DNA analysis.

The PCR result must then be verified and visualized. For this purpose, the DNA is separated by gel electrophoresis after PCR (Step 4). Because DNA is negatively charged, DNA fragments are separated in a gel matrix within an electric field according to their size. Smaller DNA fragments migrate faster through the gel, whereas larger fragments migrate more slowly. The gel contains dyes that fluoresce when bound to DNA. Under UV light, so-called bands become visible, representing the respective DNA fragments. Based on the size and number of bands, initial conclusions can already be drawn regarding the presence of the target gene and potential variants.

Further and much more detailed information about the sequences is obtained by sequencing the PCR products. For this purpose, the PCR DNA fragments are excised from the gel and purified (Step 5).

Subsequently, the sequences, i.e., the base order A, C, G, and T (the four letters of DNA sequences), of the purified DNA fragments are determined using Sanger sequencing (Step 6). The resulting DNA sequences are analysed, and finally the *FRI* sequences of the different collected plant samples are compared with each other and with the genomic DNA sequence of the reference accession Col-0 (Step 7).

The workflow (Figure 3A in the main text) graphically illustrates the individual experimental steps

Step 1: Collection of Leaf Samples

Materials:

- 1.5 ml plastic reaction tubes
- Tweezers, knife

Leaf material (approximately the size of a 1-cent coin) is transferred into a 1.5 ml plastic reaction tube. One tube is required per plant. The tubes must be clearly labelled, for example with the plant number from the list of plant locations and individuals. The leaf material should be as healthy and green as possible. Alternatively, other plant tissues may also be used. The plant samples can be stored in a refrigerator or freezer.

Step 2: Extraction of Genomic DNA:

Materials:

- 10 ml DNA extraction buffer
- Plastic hand pestle
- Sterile ultrapure water
- Micropipettes and pipette tips (200 µl, 1 ml)
- Tube rack
- Microcentrifuge for 1.5 ml tubes

To obtain genomic DNA (gDNA) from the plant samples, the plant material is first mechanically disrupted and the cells of the fragmented plant tissue are lysed so that the DNA can be released. For this purpose, 150 µl of extraction buffer are pipetted into the reaction tubes containing the leaf samples; the “magic” buffer contains sucrose, salt, and TRIS (tris(hydroxymethyl)aminomethane hydrochloride). The plant sample is ground using a plastic hand pestle until the solution becomes greenish and cloudy. Subsequently, an additional 1350 µl of ultrapure water is added to the sample and gently mixed. The samples are then centrifuged (1 minute at 5000 rpm) until the plant debris has settled as a pellet at the bottom of the tube. The supernatant is used as the template for the PCR reaction.

The sample can be stored frozen at −20 °C; before reuse, it should be thawed and centrifuged again

Step 3: Amplification of *FRI*-Gene Fragments by Polymerase Chain Reaction (PCR)

Materials:

- 2× PCR mix, e.g. RedTaq (Merck)

- Primers, e.g. 100 µM
- Sterile ultrapure water
- PCR reaction tubes
- If necessary, 1.5 ml reaction tube
- Micropipettes and pipette tips for PCR (200 µl, 20 µl)
- Tube rack
- Ice container and ice for cooling
- PCR machine
- Vortex (optional)
- Centrifuge for PCR reaction tubes (optional)

Specific regions of the *FRI*-gene are now amplified billions of times using polymerase chain reaction (PCR). The principle of PCR is described in detail in biology textbooks, and numerous instructional videos are also available (Supplemental File / Text S6). PCR reaction tubes are prepared and labelled.

A PCR reaction mixture is added, containing Taq polymerase or another thermostable DNA polymerase, buffer and salts, nucleotides (ATP, CTP, GTP, TTP), two primers (F/forward/5′ and R/reverse/3′), and water. Detailed information on concentrations and other parameters of the PCR mix is provided in the manufacturer's instructions.

If three or more PCR reactions of the same type are to be prepared, it is recommended to prepare a pooled mixture, the so-called master mix (MMX), in advance in a 1.5 ml reaction tube. This mixture contains all components required for PCR except gDNA. The master mix should be mixed thoroughly and briefly centrifuged if possible. Subsequently, 24 µl of the master mix are dispensed into the PCR reaction tubes. Finally, 1 µl of gDNA is added to each tube. After mixing (optionally using a vortex) and subsequent centrifugation (optional), the reaction tubes are placed into the PCR machine.

Caution: Pipette tips should be discarded after each use to avoid cross-contamination with gDNA!

**Table: Preparation of PCR reactions including two example master mixes**			
Component	for the number of samples (n)		
	1 (n = 1 sample)	n + 3 $ (n = 17 samples)	n + 5 $ (n = 45 samples)
2X PCR Mix*	12.5 µl	250 µl	625 µl
Primer 1**	2.5 µl	50 µl	125 µl
Primer 2**	2.5 µl	50 µl	125 µl
Sterile water	5.5 µl	110 µl	275 µl
gDNA***	2 µl	2 µl	2 µl

Total volume per reaction: 25 µl
* contains buffer, salts, nucleotides, and enzyme<br>** 10 µM<br>*** a water control without gDNA, with an additional 1 µl of ultrapure water, should also be included<br>$ Example setups for master mixes; the first number indicates the number of actual samples, and the second number indicates the number of additionally calculated samples to ensure sufficient volume after distribution for all actual samples; 24 µl of the master mix are pipetted per PCR reaction.

Primer combination used:

PCR *FRI*

G/E site_F 5′ CCATAGACGAATTAGCTGC 3′

16bp-Difference_R 5′ AGTACTCACAAGTCACAAC 3′

PCR product = 903/919 bp (903 bp with the *A. thaliana* Col-0 reference accession)

Step 4: Separation of PCR DNA Fragments by Gel Electrophoresis

Materials:

- Nitrile gloves and lab coat
- TBE electrophoresis buffer, approx. 2 L
- MIDORI Green TBE tablet
- DNA size marker
- Agarose gel electrophoresis apparatus with casting tray and comb
- Glass Erlenmeyer flask
- Graduated cylinder for 25 ml
- Micropipette (200 µl) and pipette tips
- Microwave
- Camera or mobile phone for documentation

Gel electrophoresis is an analytical method used to separate electrically charged DNA fragments from one another. In this case, the PCR fragments are separated from the primers. Using the MIDORI Green DNA staining method, the DNA fragments are made visible. A size standard is used to confirm successful amplification of the PCR fragment and to verify the size of the PCR DNA fragment.

During gel electrophoresis, **nitrile gloves and a lab coat** should always be worn!

Twenty-five milliliters of distilled water (at room temperature) are mixed with one MIDORI Green TBE tablet in a glass flask. This tablet contains the buffer required for the reaction as well as the necessary agarose concentration for a 1% agarose gel. The tablet should dissolve within 3 to 5 minutes. The solution is then

heated, for example using a microwave **(which must not be used for food preparation under any circumstances)**.

Care must be taken to gently swirl the solution occasionally and to prevent boiling over during heating. Once no particles are visible in the solution, it should be allowed to cool for 3–4 minutes and then poured into an electrophoresis casting tray fitted with combs of the appropriate size. Complete solidification of the gel takes approximately 20–30 minutes. After solidification, the combs are removed and the gel, together with the casting tray, is placed into the electrophoresis chamber. The chamber is then filled with electrophoresis buffer (so-called TBE buffer) until the gel is covered by approximately 0.5 cm of buffer and the wells are completely filled.

Next, 25 µl of PCR product are pipetted into the gel wells (**a new plastic pipette tip must be used for each sample**). Care should be taken to ensure that:

1. one gel well remains empty (the DNA size marker will be added later);
2. samples are pipetted very slowly and carefully, as the wells may otherwise overflow, leading to erroneous results;
3. work is not carried out too slowly, as samples may diffuse into the gel over time even without the electric field being applied, which would result in ambiguous final results. It is therefore very important to **prepare a written plan** in advance indicating which gel well will be filled with which sample.

Finally, 3 µl of the DNA size marker (1 kb plus) are added to the remaining empty well. The DNA size marker is a mixture of DNA fragments of known but different lengths. It serves as a control, since an incorrect migration of the marker at the end of electrophoresis indicates that something has gone wrong.

After the DNA ladder and all samples have been loaded into the gel wells, the gel is placed into the electrophoresis chamber, the lid is closed, and the chamber is switched on and run until a clear separation of the dye front is visible through the lid. This should take approximately 35 minutes. To document the results, a photograph can be taken using any camera; for optimal images, a black, foldable cover should be placed on the chamber so that the photo can be taken through an opening at the top.

A clear single DNA band of approximately the expected size should be visible for a successful PCR reaction.

PCR Program Entered into the Electrophoresis System

1. Initial denaturation: 2 minutes, 96 °C
2. 35 cycles of the following steps:
    - Denaturation: 30 seconds, 96 °C
    - Primer annealing: 35 seconds, 55 °C
    - Extension: 45 seconds, 72 °C
3. Final extension: 60 seconds, 72 °C

Step 5: Purification of PCR Fragments

Materials:

- Gel purification kit
- 1.5 ml reaction tubes
- Micropipette (200 µl) and pipette tips
- Precision balance (optional)
- Centrifuge for 1.5 ml reaction tubes
- Heating block or water bath

DNA bands are cut directly from the gel using an appropriate cutting tool (e.g., a scalpel). Only clearly visible bands should be excised, and any smear above or below the fluorescent bands should be excluded. The gel pieces, cut as closely as possible around the bands, are placed individually into **empty and labelled 1.5 ml reaction tubes**.

The mass of the excised gel piece can be determined after subtracting the empty tube weight (optional) or estimated. Gel purification is then performed according to the manufacturer's instructions of the purification kit. Typically, the gel piece is dissolved in a buffer, the DNA fragments are bound to a column, washed, and finally eluted with 10 µl of elution buffer. This results in a final concentration of approximately 3 ng of PCR DNA fragments per µl.

Step 6: DNA Sequencing by Sanger Method

Materials:

- 1.5 ml reaction tubes
- Micropipette (200 µl) and pipette tips
- Primers
- Ultrapure water
- Labels or barcodes (to be requested from a DNA sequencing service provider for Sanger DNA sequencing of individual samples)

In Sanger DNA sequencing, the base sequence of a DNA strand is determined using a sequence-specific primer. Nowadays, this reaction is typically performed by specialized companies as a service. The principle of Sanger DNA sequencing is described in detail in textbooks, educational websites, and instructional videos (Supplemental File / Text S6). For each PCR DNA fragment, two sequencing reactions are prepared in order to determine the sequences from both ends of the fragment, one using the forward (F) primer and one using the reverse (R) primer. For this purpose, two reaction tubes are prepared and labelled.

According to the specifications of the sequencing service provider, for example, 10 pmol of the F or R primer (from a 10 µM stock solution), up to 200 ng of PCR product, and ultrapure water are combined in a volume defined by the sequencing company. The reaction tubes are then labelled, for example with barcodes or labels

provided by the sequencing service and sent for Sanger sequencing. Subsequently, the results are made available electronically, including the corresponding barcode numbers

Step 7: Sequence Analysis of *FRI*-Specific DNA Fragments

After receiving the sequencing data, the forward and reverse sequences are further processed to generate analysable DNA sequences. Low-quality sequence regions at the ends of the reads are removed, and a consensus sequence in FASTA format is generated for each sample. Processing and assembly of the sequence data are performed using the freely available Benchling platform (Benchling [Biology Software]. (2025). Retrieved from https://benchling.com.), which enables visualization, editing, and comparison of DNA sequences.

For comparative analysis, the reference sequence of the *FRIGIDA* (*FRI*) gene from the *Arabidopsis thaliana* reference accession Col-0 is obtained from The Arabidopsis Information Resource (TAIR, www.arabidopsis.org, AT4G00650). The consensus sequences generated for the individual plant samples are then aligned against the Col-0 reference sequence using the program AliView (Larsson et al., 2014). Multiple sequence alignment is used to identify sequence differences among the analysed samples which may be indicating polymorphisms.

To support interpretation and presentation of the results, a simplified graphical representation of allele differences is generated based on the alignments. This approach allows for a clear visualization of genetic variation and is particularly suitable for use in an educational (school) context.

Text S6. Examples of illustrating texts and videos useful for teaching the basic genetic concepts and techniques used in this school project as well as free statistical resources.

Examples of English texts which demonstrate genetic concepts and laboratory techniques

Anonymous (2020). The DNA book. Penguin Random House.

Baker, C. (1997). Your genes, your choices: Exploring the issues raised by genetic research. American Association for the Advancement of Science. https://www.aaas.org/sites/default/files/yourgenes.pdf

Barwell, J. & Lakhani, N. (2024). What is DNA? Wayland.

Gonick, L. (1991). The cartoon guide to genetics. William Morrow Paperbacks.

Green, M. R. & Sambrook, J. (2012). Molecular cloning: A laboratory manual. Cold Spring Harbor Laboratory Press.

Häfner, C. & Scheier, M. (2024). The code of life: All about genes, DNA, genetic engineering, and why you are the way you are. Flying Eye Books.

Johnson, R. L. (2007). Amazing DNA. Millbrook Press.

Kratz, R. F. & Spock, L. (2023). Genetics for dummies. Fourth edition. Wiley.

Mooney, C. (2014). Genetics: Breaking the code of your DNA. Nomad Press.

Skwarecki, B. (2018). Genetics 101: From chromosomes and the double helix to cloning and DNA tests, everything you need to know about genes. Adams Media.

Webb, W. (2024). A slacker's guide to genetics: A beginner's guide to genetics. SL Editions.

For further suggestions for appropriate children's books, see, e.g.:

PBS Books (2024). The gene children's book list. https://www.pbsbooks.org/the-gene-childrens-book-list/

For adult-level textbooks, see, e.g.:

Bio Explorer (2024). Top 12 best genetics textbooks. https://www.bioexplorer.net/best-genetics-textbooks.html/

Book Authority (2024). 20 best genetics books of all time. https://bookauthority.org/books/best-genetics-books

Examples of German texts which demonstrate genetic concepts and laboratory techniques

Boonen, S. (2024). Billie und seine Gene: Von Fruchtfliegen, Sommersprossen und dem Bauplan des Lebens. Mintvision.

Da Silva, C. C. (2023). Mein wunderbares Ich – Was mich ausmacht und welche Rolle die Gene dabei spielen. cbj.

Eberhard-Metzger, C. (2001). WAS IST WAS, Band 111: Die Gene. Tessloff.

Gilbert, S. & Woollard, A. (2020). Superschlaue Gene. Superstark & superschlau. Dorling Kindersley Verlag.

Häfner, C. & Scheier, M. (2022). Der Code des Lebens: Alles über Gene, DNA, Gentechnik und warum du so bist, wie du bist. 2. Edition. Knesebeck.

Wünschiers, R. (2019). Gentechnik: Gene lesen, schreiben und editieren. Springer Spektrum, Wiesbaden, Germany.

Examples of English videos which demonstrate genetic concepts and laboratory techniques

How to Use a Micropipette https://www.youtube.com/watch?v=Jfqafjt4q6U

How to pipette correctly – a short step-by-step introduction into proper pipetting https://www.youtube.com/watch?v=QGX490kuKjg

Gel Electrophoresis Explained https://www.youtube.com/watch?v=eImaWphK2h4

What is Polymerase Chain Reaction? | PCR Explained https://www.youtube.com/watch?v=hO3mTqrEeq8 (This is one of numerous videos by the channel called '2 Minute Classroom' which cover many of the topics relevant here.)

DNA, Chromosomes, Genes, and Traits: An Intro to Heredity https://www.youtube.com/watch?v=8m6hHRlKwxY (This is one of numerous videos by the channel called 'Amoeba Sisters' which cover many of the topics relevant here.)

PCR (Polymerase Chain Reaction) Explained https://www.youtube.com/watch?v=8GOKaZ8MRyM (This is one of numerous videos by the channel called 'BOGObiology' which cover many of the topics relevant here.)

Next Generation Sequencing - A Step-By-Step Guide to DNA Sequencing. https://www.youtube.com/watch?v=WKAUtJQ69n8 (This is one of numerous videos by the channel called 'ClevaLab' which cover many of the topics relevant here.)

Transcription and Translation: From DNA to Protein https://www.youtube.com/watch?v=bKIpDtJdK8Q (This is one of numerous videos by the channel called 'Professor Dave explains' which cover many of the topics relevant here.)

What is a Chromosome? https://www.youtube.com/watch?v=IePMXxQ-KWY (This is one of numerous videos by the channel called 'Stated Clearly' which cover many of the topics relevant here.)

DNA Sequencing - 3D https://www.youtube.com/watch?v=ONGdehkB8jU (This is one of numerous videos by the channel called 'yourgenome' which cover many of the topics relevant here.)

GCSE Biology - DNA Part 1 - Genes and the Genome #63 https://www.youtube.com/watch?v=wv1TQXBQ6wQ&t=116s

GCSE Biology - What is DNA? (Structure and Function of DNA) #65 https://www.youtube.com/watch?v=T6_wKPAbf2k

How are Proteins Made? - Transcription and Translation Explained #66 https://www.youtube.com/watch?v=ubdoUqmNF98

Chromosomes: structure and function https://www.youtube.com/watch?v=d_-XWjlhLyk

What happens when your DNA is damaged? - Monica Menesini https://www.youtube.com/watch?v=vP8-5Bhd2ag

JoVE (Journal of Visualized Experiments) https://www.youtube.com/@JoVEJournal, https://app.jove.com/education/science-education/basic-biology/general-laboratory-techniques

Examples of German videos which demonstrate genetic concepts and laboratory techniques

Pipettieren (Dr. Andreas Czech) https://lecture2go.uni-hamburg.de/l2go/-/get/v/22516

DNA Polymerase https://studyflix.de/biologie/dna-polymerase-2471

Polymerase Kettenreaktion (PCR) https://studyflix.de/biologie/polymerase-kettenreaktion-pcr-2473

Polymerase-Kettenreaktion (PCR) - Biochemie - Labormethoden - AMBOSS Video https://www.youtube.com/watch?v=OwFMJC7SQ_I

Gelelektrophorese https://studyflix.de/biologie/gelelektrophorese-2584/video

Agarose-Gelelektrophorese (Dr. Patrick Ziegelmüller) https://lecture2go.uni-hamburg.de/l2go/-/get/v/22517

Gelelektrophorese - [Verfahren zur DNA-Analyse] - [Biologie, Gentechnik] https://www.youtube.com/watch?v=xjHIZOBweu4

DNA Sequenzierung https://studyflix.de/biologie/dna-sequenzierung-2485

Sanger Sequenzierung https://studyflix.de/biologie/sanger-sequenzierung-2486

Klassische DNA-Sequenzierung nach Sanger https://www.youtube.com/watch?v=JcfnrJDTPG0

Free statistics textbooks and resources

Basten, G. (2010). Introduction to scientific research projects. **PDF-version**: https://bookboon.com/en/introduction-to-scientific-research-projects-ebook

Bhattacherjee, A. (2012). Social science research: principles, methods, and practices. Textbooks Collection. 3. **PDF-version**: https://digitalcommons.usf.edu/cgi/viewcontent.cgi?article=1002&context=oa_textbooks

Boettinger, J. (2019-2020). Math and statistics guides from UB's Math & Statistics Center. **Online and PDF-version**: https://ubalt.pressbooks.pub/mathstatsguides/

Çetinkaya-Rundel, M. & Hardin, J. (2024). Introduction to modern statistics. Second edition. OpenIntro. **Online-version**: https://openintro-ims.netlify.app/

Cote, L. R., Gordon, R., Randell, C. E., Schmitt, J. & Marvin, H. (2021). Introduction to statistics in the psychological sciences. Open Educational Resources Collection. **Online-version**: https://umsystem.pressbooks.pub/isps/, **PDF-version**: https://irl.umsl.edu/cgi/viewcontent.cgi?article=1024&context=oer

da Silva, J. G. C. (2024). Scientific research: An introduction. First edition. B P International. **PDF-version**: https://stm.bookpi.org/SRAI/article/view/15080/2797, **DOI**: https://doi.org/10.9734/bpi/mono/978-81-976007-6-0

Fein, E. C., Gilmour, J., Machin, T. & Hendry, L. (2021). Statistics for research students. University of Southern Queensland, Toowoomba, Queensland, Australia. **PDF-version**: https://usq.pressbooks.pub/statisticsforresearchstudents/

Ford, E. D. (2000). Scientific method for ecological research. Cambridge University Press, Cambridge, UK. **PDF-version**: http://assets.cambridge.org/97805216/60051/sample/9780521660051wsc00.pdf

Herzog, M. H., Francis, G. & Clarke, A. (2019). Understanding statistics and experimental design: How to not lie with statistics. Springer, Cham, Switzerland. **PDF-version**: https://link.springer.com/book/10.1007/978-3-030-03499-3

Huff, D. (1954). How to lie with statistics. W. W. Norton & Company, New York, New York, USA. **PDF-version**: https://materias.df.uba.ar/l1aa2017c2/files/2017/08/Huff-HowToLieWithStatistics.pdf

Illowsky, B. & Dean, S. (2023). Introductory statistics 2e. OpenStax, Rice University, Houston, Texas, USA. **Online and PDF-version**: https://openstax.org/details/books/introductory-statistics-2e

Kerns, G. J. (2010). Introduction to probability and statistics using R. First edition. **PDF-version**: https://www.atmos.albany.edu/facstaff/timm/ATM315spring14/R/IPSUR.pdf

Krebs, C. J. (1999). Ecological methodology, second edition. Benjamin/Cummings, Menlo Park, California, USA. **PDF-version**: https://de.scribd.com/document/363368222/Ecological-Methodology-Second-Edition-pdf

Krebs, C. J. (2014). Ecological methodology, third edition. **Online-version**: https://www.zoology.ubc.ca/~krebs/books.html

Lane, D. M. (2003). Introduction to statistics. Rice University, Houston, Texas, USA. **PDF-version**: https://onlinestatbook.com/Online_Statistics_Education.pdf

Lepine, M. (2022). College statistics. St. Clair College AA&T, Windsor, Ontario, Canada. **PDF-version**: https://ecampusontario.pressbooks.pub/sccstatistics/

McDonald, J. H. (2008). Handbook of biological statistics. Sparky House Publishing Baltimore, Maryland, USA. **PDF-version**: https://e-ilami.unissa.edu.bn:8443/bitstream/handle/20.500.14275/6724/McDonald,%20J.H.%202014.%20Handbo

ok%20of%20Biological%20Statistics,%203rd%20ed.%20Sparky%20House%20Publishing%20new2Fx8.pdf?sequence=3; https://www.biostathandbook.com/

McDonald, J. H. (2014). Biological statistics. LibreTexts. **PDF-version**: https://stats.libretexts.org/Bookshelves/Applied_Statistics/Biological_Statistics_(McDonald)

Quinn, G. P. & Keough, M. J. (2003). Experimental design and data analysis for biologists. Cambridge University Press, Cambridge, UK. **PDF-version**: https://www2.ib.unicamp.br/profs/fsantos/apostilas/Quinn%20&%20Keough.pdf

Rajasekar, S., Philominathan, P. & Chinnathambi, V. (2006). Research methodology. **PDF-version**: https://www.researchgate.net/publication/2174858_Research_Methodology

Ruxton, G. D. & Stafford, J. (2015). Statistics for school biology experiments and advanced higher projects. SSERC, Dumfermline, UK. **PDF-version**: https://www.stem.org.uk/system/files/elibrary-resources/2017/11/Statistics%20book%20final.pdf

Seefeld, K. & Linder, E. (2007). Statistics using R with biological examples. University of New Hampshire, Durham, New Hampshire, USA. **PDF-version**: https://cran.r-project.org/doc/contrib/Seefeld_StatsRBio.pdf

Seltman, H. J. (2018). Experimental design and analysis. **PDF-version**: https://www.stat.cmu.edu/~hseltman/309/Book/Book.pdf

Thulin, M. (2024). Modern Statistics with R: From wrangling and exploring data to inference and predictive modelling. Second edition. **Online-version**: https://modernstatisticswithr.com/

Townend, J. (2013). Practical statistics for environmental and biological scientists. John Wiley & Sons. **Online-version**: https://books.google.de/books?hl=en&lr=&id=R9Uwc8FlYQcC&oi=fnd&pg=PR9&dq=biology+statistics+for+school+students&ots=YQcJOaxE34&sig=ZIlxeksU1r00vBnacZoPQD243HQ&redir_esc=y#v=onepage&q&f=false

Van De Schoot, R. & Miočević, M. (2020). Small sample size solutions: A guide for applied researchers and practitioners. Routledge, London, UK. **PDF-version**: https://www.taylorfrancis.com/reader/download/52ba38d0-e3c0-4b45-aaf4-91c1c31e8fd3/book/pdf?context=ubx

Walliman, N. (2011). Research methods: the basics. Routledge. **PDF-version**: http://dspace.vnbrims.org:13000/jspui/bitstream/123456789/4667/1/Research%20Methods%20The%20Basics%20-Nicholas%20Walliman.pdf

Wasserman, L. (2004). All of statistics. Springer. **PDF-version**: https://egrcc.github.io/docs/math/all-of-statistics.pdf

Watts, V. (2022). Introduction to statistics: An Excel-based approach. Fanshawe College Pressbooks, London, Ontario, Canada. **PDF-version**: https://ecampusontario.pressbooks.pub/introstats/

Webb, R. (2023). Mostly harmless elementary statistics. **PDF-version**: https://www.infobooks.org/free-pdf-books/math/statistics/

Webb, R. (2023). Mostly harmless statistics. **Online-version**: https://stats.libretexts.org/Bookshelves/Introductory_Statistics/Mostly_Harmless_Statistics_(Webb)

Wessa, P. (2016). Box-Cox Normality Plot - Free Statistics Software (Calculator) (v1.1.12) in Free Statistics Software (v1.2.1). **Online-version**: http://www.wessa.net/rwasp_boxcoxnorm.wasp

Supplement References

Arabidopsis Genome Initiative (2000). Analysis of the genome sequence of the flowering plant *Arabidopsis thaliana*. *Nature* 408, 796-815.

Ballard, H. L., Dixon, C. G., and Harris, E. M. (2017). Youth-focused citizen science: Examining the role of environmental science learning and agency for conservation. *Biol. Conserv.* 208, 65-75.

Barnard, C., Gilbert, F. & Mcgregor, P. (2011). Asking questions in biology. A guide to hypothesis testing, experimental design and presentation in practical work and research projects. Fourth edition. Pearson Education Ltd.

Bryce, C. M., Baliga, V. B., De Nesnera, K. L., Fiack, D., Goetz, K., Tarjan, L. M., et al. (2016). Exploring models in the biology classroom. *American Biology Teacher* 78, 35-42.

Caicedo, A. L., Stinchcombe, J. R., Olsen, K. M., Schmitt, J., and Purugganan, M. D. (2004). Epistatic interaction between *Arabidopsis FRI* and *FLC* flowering time genes generates a latitudinal cline in a life history trait. *Proceedings of the National Academy of Sciences* 101, 15670-15675.

Englefield, B., Starling, M., Wilson, B., Roder, C., and McGreevy, P. (2020). The Australian roadkill reporting project—Applying integrated professional research and citizen science to monitor and mitigate roadkill in Australia. *Animals* 10, 1112.

Flowers, S. K., Beyer, K. M., Pérez, M., and Jeffe, D. B. (2016). Early environmental field research career exploration: An analysis of impacts on precollege apprentices. *CBE—Life Sciences Education* 15(4), ar67. Graf, E. (2016). *99 Tipps: Für Biologie*. Cornelsen, Berlin, Germany.

Graphpad (2025). Linear regression calculator. https://www.graphpad.com/quickcalcs/linear1/

Guy-Gaytán, C., Gouvea, J. S., Griesemer, C., and Passmore, C. (2019). Tensions between learning models and engaging in modeling: Exploring implications for science classrooms. *Science & Education* 28, 843-864.

Hsu, C.-H., and Lin, T.-E. (2023). What people learn from death: Exploring citizen scientists' learning outcomes in Taiwan Roadkill Observation Network from an environmental education perspective. *Environ. Educ. Res.* 1-15.

Johanson, U., West, J., Lister, C., Michaels, S., Amasino, R., and Dean, C. (2000). Molecular analysis of *FRIGIDA*, a major determinant of natural variation in *Arabidopsis* flowering time. *Science* 290, 344-347.

Johnson, J. B., and Omland, K. S. (2004). Model selection in ecology and evolution. *Trends Ecol. Evol.* 19, 101-108.

Klamann, L. J. (2024). Variation of the FRIGIDA gene in urban populations of *Arabidopsis thaliana*: a pilot study for a citizen science project. Bachelor thesis. Heinrich-Heine-Universität Düsseldorf, Germany.

Knopf, M. C., and Bauer, P. (2025). Affordable non-invasive machine-aided phenotyping identifies phenotypic variation to soil stress across the *Arabidopsis thaliana* life cycle. Physiol. Plant. 177, e70427.

Koornneef, M., and Meinke, D. (2010). The development of *Arabidopsis* as a model plant. *Plant Journal* 61, 909-921.

Kover, P. X., and Schaal, B. A. (2002). Genetic variation for disease resistance and tolerance among *Arabidopsis thaliana* accessions. *Proceedings of the National Academy of Sciences* 99, 11270-11274.

Krämer, U. (2015). Planting molecular functions in an ecological context with *Arabidopsis thaliana*. *eLife* 4, e06100.

Lüsse, M., Brockhage, F., Beeken, M., and Pietzner, V. (2022). Citizen science and its potential for science education. *Int. J. Sci. Educ.* 44, 1120-1142.

Mattioli, R., Francioso, A., and Trovato, M. (2022). Proline affects flowering time in *Arabidopsis* by modulating FLC expression: a clue of epigenetic regulation? *Plants* 11, 2348.

Millennium Ecosystem Assessment (2005). *Ecosystems and human well-being: A framework for assessment*. Island Press, Washington, D.C., USA.

Passmore, C., Stewart, J., and Cartier, J. (2009). Model-based inquiry and school science: Creating connections. *School Science and Mathematics* 109, 394-402.

Provart, N. J., Alonso, J., Assmann, S. M., Bergmann, D., Brady, S. M., Brkljacic, J., et al. (2016). 50 years of Arabidopsis research: highlights and future directions. *New Phytologist* 209, 921-944.

Puhakka, R., and Hakokongäs, E. (2024). Adolescents' experiences in nature: Sources of everyday well-being. *J. Leis. Res.* 55, 250-269.

Quillin, K., and Thomas, S. (2015). Drawing-to-learn: a framework for using drawings to promote model-based reasoning in biology. *CBE—Life Sciences Education* 14, es2.

Schmidt, G. (2014). The emergent patterns of climate change. https://www.youtube.com/watch?v=JrJJxn-gCdo

Schmitz, R. J., Schultz, M. D., Urich, M. A., Nery, J. R., Pelizzola, M., Libiger, O., et al. (2013). Patterns of population epigenomic diversity. *Nature* 495, 193-198.

Schuttler, S. G., Sears, R. S., Orendain, I., Khot, R., Rubenstein, D., Rubenstein, N., et al. (2019). Citizen science in schools: Students collect valuable mammal data for science, conservation, and community engagement. *BioScience* 69, 69-79.

Shindo, C., Aranzana, M. J., Lister, C., Baxter, C., Nicholls, C., Nordborg, M., et al. (2005). Role of *FRIGIDA* and *FLOWERING LOCUS C* in determining variation in flowering time of Arabidopsis. *Plant Physiology* 138, 1163-1173.

Sieg, A.-K., and Dreesmann, D. (2021). Promoting pro-environmental BEEhavior in school. Factors leading to eco-friendly student action. *Sustainability* 13, 6598.

Sjöblom, P., and Wolff, L.-A. (2017). “It wouldn't be the same without nature”—The value of nature according to Finnish upper secondary school students. *Int. J. Environ. Educ.* 48, 322-333.

Soanes, K., Cranney, K., Dade, M. C., Edwards, A. M., Palavalli-Nettimi, R., and Doherty, T. S. (2020). How to work with children and animals: A guide for school-based citizen science in wildlife research. *Austral Ecol.* 45, 3-14.

Social Science Statistics (2025a). Spearman's Rho Calculator. https://www.socscistatistics.com/tests/spearman/default2.aspx

Social Science Statistics (2025b). Mann-Whitney U Test Calculator. https://www.socscistatistics.com/tests/mannwhitney/

Svoboda, J., and Passmore, C. (2013). The strategies of modeling in biology education. *Science & Education* 22, 119-142.

Symes, L., Serrell, N., and Ayres, M. (2015). A practical guide for mentoring scientific inquiry. *Bull. Ecol. Soc. Am.* 96, 352-367.

Thieme, M., Brêchet, A., Bourgeois, Y., Keller, B., Bucher, E., and Roulin, A. C. (2022). Experimentally heat-induced transposition increases drought tolerance in *Arabidopsis thaliana*. *New Phytologist* 236, 182-194.

Tomiolo, S., Damgaard, C. F., Gay, L., Ronfort, J., and Ehlers, B. K. (2022). A plant growth model to test for changes in plant–plant interaction over a growing season: the case of kin competition. *Oikos* 2022, e09358. Walther, B. A., Boëte, C., Binot, A., By, Y., Cappelle, J., Carrique-Mas, J. J., et al. (2016). Biodiversity and health: Lessons and recommendations from an interdisciplinary conference to advise Southeast Asian research, society and policy. *Infect. Genetics Evol.* 40, 29-46. 10.1016/j.meegid.2016.02.003

Undercurrents Media (2010). The impossible hamster (and economic growth). https://www.youtube.com/watch?v=bqz3R1NpXzM

van Eijck, M., and Roth, W.-M. (2009). Authentic science experiences as a vehicle to change students' orientations toward science and scientific career choices: Learning from the path followed by Brad. *Cultural Studies of Science Education* 4, 611-638.

Walther, B. A., and Yang, H. (2024). Testing a simple and non-intrusive method for monitoring the breeding season of Barn Swallows in an urban area in Kaohsiung, Taiwan. *TW J. Biodivers.* 26, 53-84.

Watson, J. M., and Moritz, J. B. (1998). The beginning of statistical inference: Comparing two data sets. *Educational Studies in Mathematics* 37, 145-168.
