# Supplementary material for: A Research Project for Secondary Schools on Urban Plant Ecological Genetics
Source: Ecol Evol. 2026 Oct 18;16(10):e74482. doi: 10.1002/ece3.74482 (PMC13678418; doi:10.1002/ece3.74482)
Supplement: Supplementary file 5 [file ECE3-16-e74482-s005.pdf]

Table S1. Important scientific skills which secondary school students need to learn during science lessons, e.g., biology, chemistry, earth and environmental sciences, physics.

Scientific skill	Explanation
Close inspection	A thorough and detailed examination of an object or phenomenon to gather precise information.
Targeted description	The precise and focused articulation of specific characteristics or aspects of an object or phenomenon.
Concentrated observation	The focused and sustained monitoring of a particular subject to gather detailed data over a period of time.
Systematic logging	The organized and consistent recording of observations, measurements, or experimental results, usually in tables, spreadsheets, logbooks, laboratory notes, etc.
Criteria-guided comparison	The evaluation of objects or phenomena based on a set of predefined standards or benchmarks to determine similarities and differences.
Careful interpretation	The thoughtful and meticulous analysis of data and observations to derive meaningful conclusions, e.g., confirmation or rejection of a hypothesis or alternative hypotheses.
Logical, scientific reasoning	The application of structured and rational thinking, based on empirical evidence and established scientific principles, to draw conclusions and make predictions.

Based on reference textbooks on teaching science:

Dawson, V. & Donovan, J. (eds) (2020). The art of teaching science. 3rd edition. Routledge.

Hassard, J. & Dias, M. (2009). The art of teaching: Inquiry and innovation in middle school and high school. Second edition. Routledge, New York, New York, USA.

Krajcik, J. S. & Czerniak, C. M. (2018). Teaching science in elementary and middle school: A project-based learning approach. 5th edition. Routledge, New York, New York, USA.

Lawson, A. E. (2010). Teaching inquiry science in middle and secondary schools. SAGE Publications, Thousand Oaks, California, USA.

Parkinson, J. (2003). Improving secondary science teaching. Routledge, London, UK.
