# Supplementary material for: A Research Project for Secondary Schools on Urban Plant Ecological Genetics
Source: Ecol Evol. 2026 Oct 18;16(10):e74482. doi: 10.1002/ece3.74482 (PMC13678418; doi:10.1002/ece3.74482)
Supplement: Supplementary file 6 [file ECE3-16-e74482-s006.pdf]

Table S3. Nine locations where *A. thaliana* individuals were sampled in Düsseldorf. Sampling took place in April 2022 (Weirauch, 2022) and from 20-26 March (calendar week CW 12) to 1-7 May (calendar week CW 18) 2023 (Klamann, 2024). Column 4 gives the date of sampling during 2022. The numbers in column 5 give the calendar weeks of each visit during 2023 (‘none’ means no individuals were found during any visit).

Location Name	Latitude N	Longitude E	2022 April	CW 2023	Address	Short description (original letter designation)
Eller J1	51.198033	6.839063	13	16-18	Karlsruherstraße 42	urban; small patch of grass with one roadside tree surrounded by road and pedestrian walkway
Vennhausen F2	51.214152	6.840804	13	12-17	Königsbergerstraße 221	urban; roadside of small, paved street going through allotment gardens
Hafen G3	51.215888	6.756049	11	12-17	Am Handelshafen	urban harbour; within cobbled pavement near a house façade with overhanging roof
Carlstadt D4	51.222367	6.768254	11	none	Mannesmannufer 5	urban; next to a tree growing within public walkway made of gravel
Oberkassel I5	51.229147	6.741788	11	13-17	Rheinallee 105	urban; sloped meadow stretching along Rhine River
Stadtmitte E6	51.230796	6.788266	12	none	Malkastenstraße 7	urban; small patch of grass next to the small inner-city street
Ludenberg A7	51.255562	6.864820	6	12-17	Blanckertzstraße 26	suburban: in between pavement stones of emergency access road
Mörsenbroich C8	51.259043	6.807244	12	15-17	Münsterstraße 434	urban; small patch of grass with one roadside tree surrounded by road and pedestrian walkway
Flughafen H9	51.278223	6.774053	12	13-17	near DUS Airport	small park near airport: in shade of trees and between pavement stones around park bench
