# Supplementary material for: A Research Project for Secondary Schools on Urban Plant Ecological Genetics
Source: Ecol Evol. 2026 Oct 18;16(10):e74482. doi: 10.1002/ece3.74482 (PMC13678418; doi:10.1002/ece3.74482)
Supplement: Supplementary file 7 [file ECE3-16-e74482-s007.pdf]

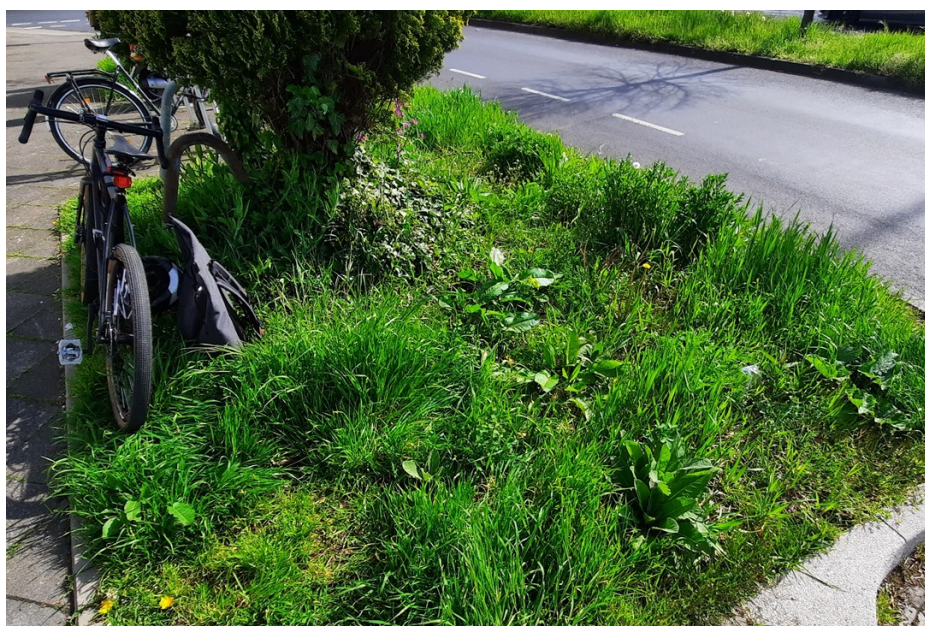

Location 1

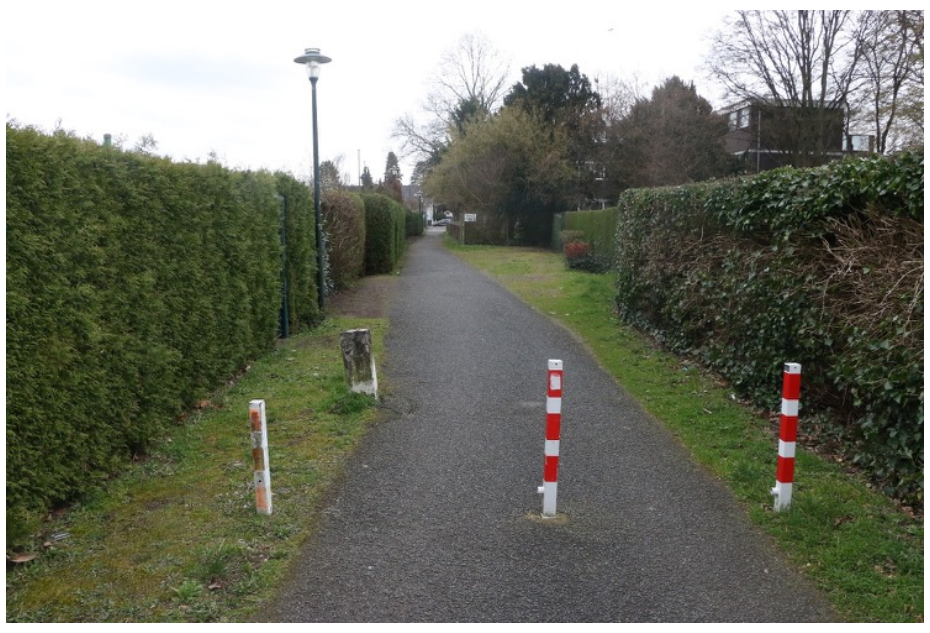

Location 2

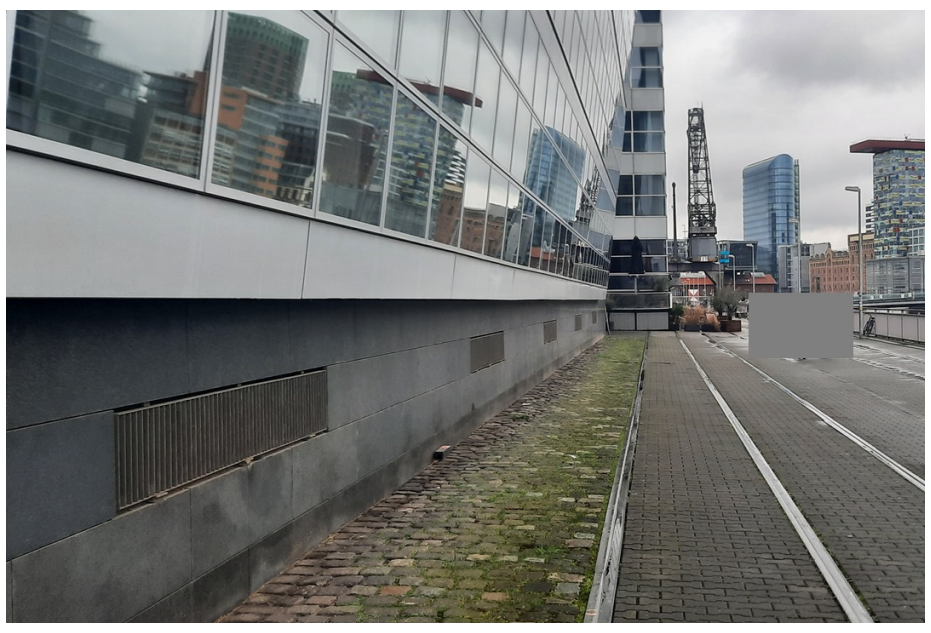

Location 3

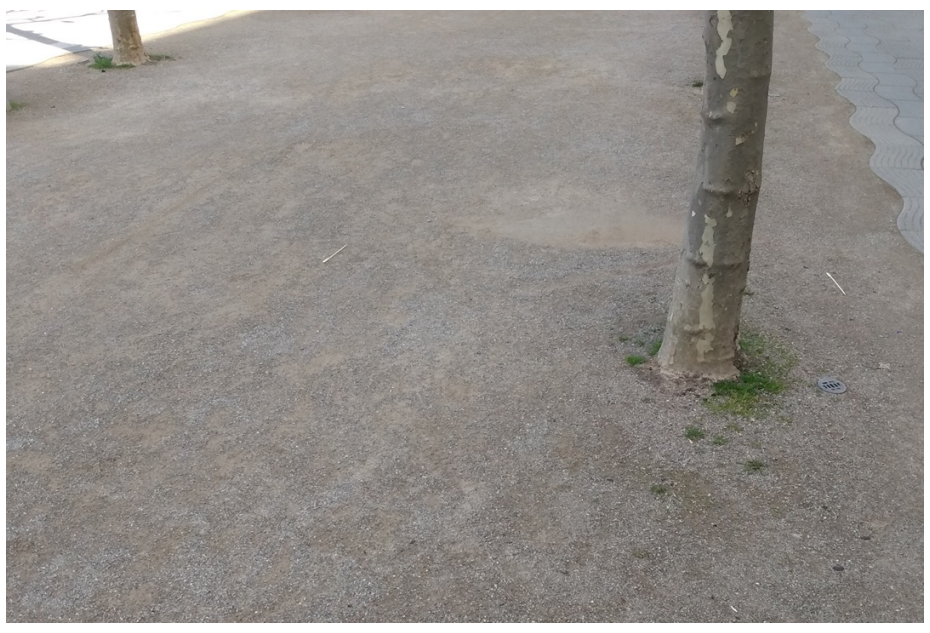

Location 4

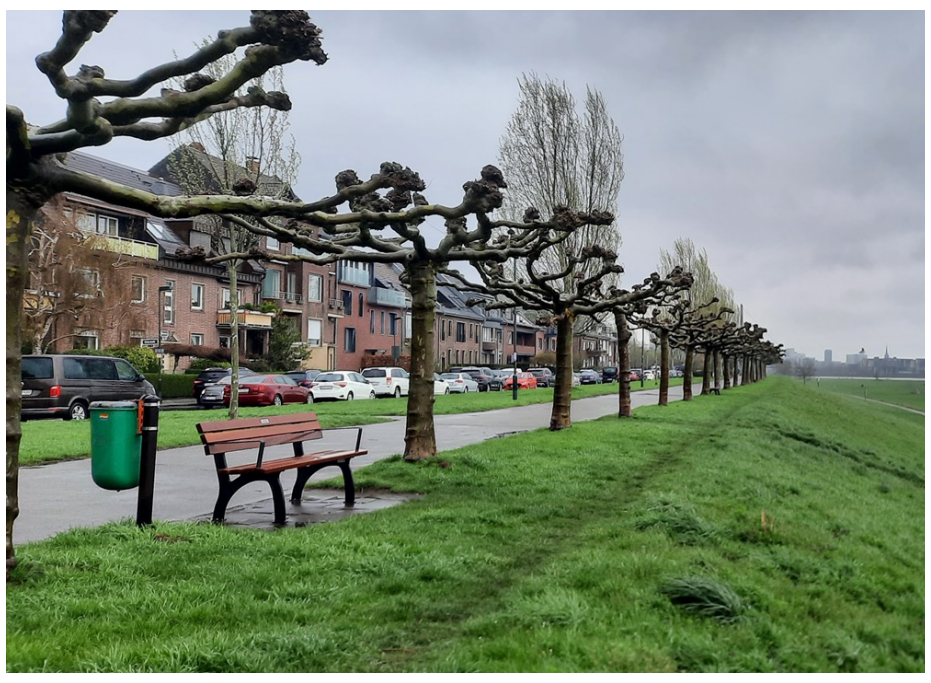

Location 5

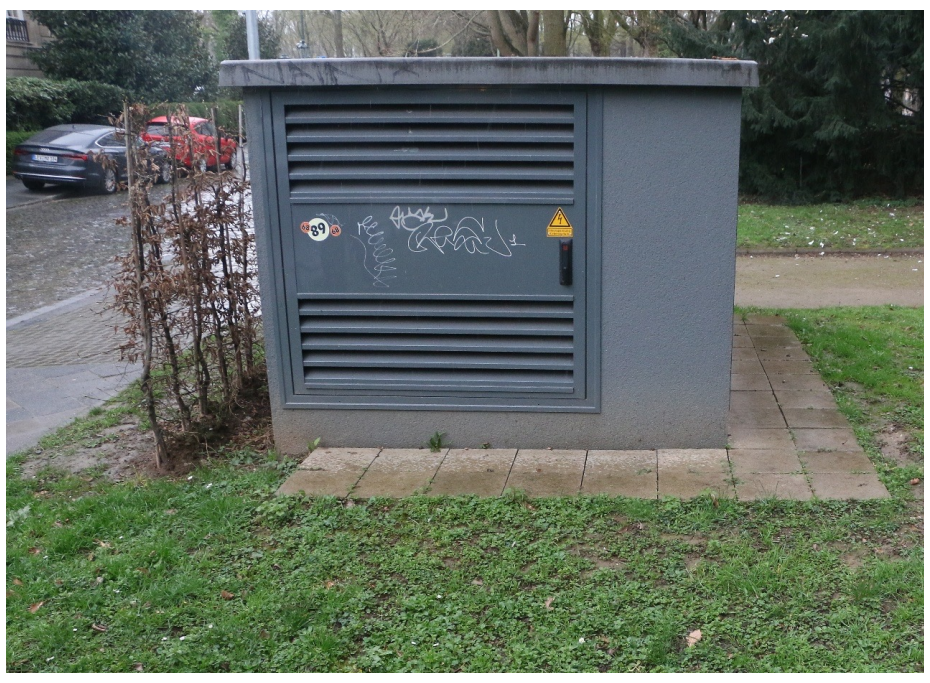

Location 6

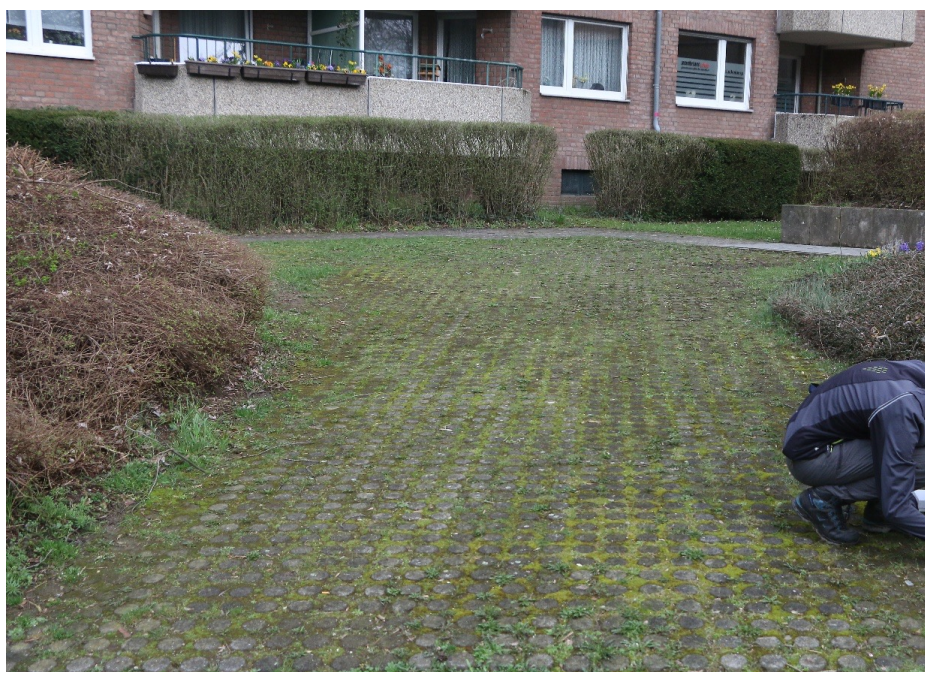

Location 7

Figure S1. Photos of the 9 locations where *A. thaliana* individuals were sampled in Düsseldorf in 2022 and 2023. For additional information on sampling sites see Figure 2A; Table S3 for geographic coordinates and details.

Location 1. *S*mall patch of grass next to roadside tree surrounded by road and pedestrian walkway. In 2022, ≤ 5 *A. thaliana* individuals grew here (photo by L. Klamann).

Location 2. Along both roadsides of a small, paved street going through allotment gardens. In 2022, > 30 *A. thaliana* individuals grew here (photo by B. A. Walther).

Location 3. Cobbled pavement near a house façade with overhanging roof. In 2022, ≤ 5 *A. thaliana* individuals grew here (photo by L. Klamann).

Location 4. Tree bed on public walkway made of gravel. In 2022, 11 to 30 *A. thaliana* individuals grew here, but none in 2023 (photo by S. Weirauch).

Location 5. Sloped meadow stretching along the Rhine River. In 2022, 12 *A. thaliana* individuals grew here (photo by L. Klamann).

Location 6. Small patch of grass next to the small inner-city street. In 2022, 6 to 10 *A. thaliana* individuals grew here, but none in 2023 (photo by B. A. Walther).

Location 7. Pavement stones of this emergency access road. In 2022, 11 to 30 *A. thaliana* individuals grew here (photo by B. A. Walther).
