# Supplementary material for: A Research Project for Secondary Schools on Urban Plant Ecological Genetics
Source: Ecol Evol. 2026 Oct 18;16(10):e74482. doi: 10.1002/ece3.74482 (PMC13678418; doi:10.1002/ece3.74482)
Supplement: Supplementary file 8 [file ECE3-16-e74482-s008.pdf]

Table S4. Simple linear regression analyses of morphological characteristics (or variables) of *A. thaliana* individuals measured at seven locations in Düsseldorf in 2023 (Dataset S1, 2023 data). Each data point is one individual plant measured at one time point for each of its morphological characteristics. All variables were Box-Cox transformed. Negative correlations are given in italic letters, and significant correlations (P < 0.05) are given in bold letters.

Variables	N	Std. Coeff.	R^2	F-value	P-value
Stem length vs.					
Number of branches	**155**	**0.627**	**0.39**	**98.9**	**< 0.0001**
Number of stem leaves	**155**	**0.529**	**0.28**	**59.6**	**< 0.0001**
Number of rosette leaves	***124***	***-0.400***	***0.16***	***23.2***	***< 0.0001***
Number of inflorescences	**155**	**0.570**	**0.33**	**73.8**	**< 0.0001**
Number of siliques	**155**	**0.714**	**0.51**	**159.2**	**< 0.0001**
Rosette diameter	**67**	**0.478**	**0.23**	**19.2**	**< 0.0001**
Silique length	**112**	**0.511**	**0.26**	**38.9**	**< 0.0001**
Number of branches vs					
Number of stem leaves	**155**	**0.572**	**0.33**	**74.4**	**< 0.0001**
Number of rosette leaves	*124*	*-0.098*	*0.01*	*1.2*	*0.28*
Number of inflorescences	**155**	**0.819**	**0.67**	**311.3**	**< 0.0001**
Number of siliques	**155**	**0.431**	**0.19**	**34.9**	**< 0.0001**
Rosette diameter	**67**	**0.584**	**0.34**	**33.7**	**< 0.0001**
Silique length	**112**	**0.344**	**0.12**	**14.7**	**0.0002**
Number of stem leaves vs.					
Number of rosette leaves	124	0.138	0.02	2.4	0.13
Number of inflorescences	**155**	**0.614**	**0.38**	**92.6**	**< 0.0001**
Number of siliques	**155**	**0.166**	**0.03**	**4.3**	**0.04**
Rosette diameter	**67**	**0.591**	**0.35**	**34.9**	**< 0.0001**
Silique length	112	0.159	0.03	2.8	0.09
Number of rosette leaves vs					
Number of inflorescences	124	0.052	0.003	0.3	0.56
Number of siliques	***124***	***-0.689***	***0.48***	***110.3***	***< 0.0001***
Rosette diameter	67	-0.016	0.0003	0.02	0.90

Silique length	***88***	***-0.363***	***0.13***	***13.0***	***0.0005***
Number of inflorescences vs.					
Number of siliques	**155**	**0.334**	**0.11**	**19.2**	**< 0.0001**
Rosette diameter	**67**	**0.546**	**0.30**	**27.6**	**< 0.0001**
Silique length	**112**	**0.249**	**0.06**	**7.3**	**0.008**
Number of siliques vs.					
Rosette diameter	**67**	**0.287**	**0.08**	**5.8**	**0.02**
Silique length	**112**	**0.627**	**0.39**	**71.3**	**< 0.0001**
Rosette diameter vs.					
Silique length	33	0.225	0.05	1.6	0.21
